# Automatic learning mechanisms for flexible human locomotion

**DOI:** 10.1101/2023.09.25.559267

**Published:** 2023-09-25

**Authors:** Cristina Rossi, Kristan A. Leech, Ryan T. Roemmich, Amy J. Bastian

**Affiliations:** a Department of Neuroscience, The Johns Hopkins University School of Medicine, Baltimore, MD, 21205, USA;; b Center for Movement Studies, Kennedy Krieger Institute, Baltimore, MD, 21205, USA;; c Division of Biokinesiology and Physical Therapy, University of Southern California, Los Angeles, CA, 90033, USA;; d Neuroscience Graduate Program, University of Southern California, Los Angeles, CA, 90007, USA;; e Department of Physical Medicine and Rehabilitation, The Johns Hopkins University School of Medicine, Baltimore, MD, 21205, USA

**Keywords:** Biological Sciences, Neuroscience, Adaptation, Motor control, Motor learning, Human locomotion, Perception

## Abstract

Movement flexibility and automaticity are necessary to successfully navigate different environments. When encountering difficult terrains such as a muddy trail, we can change how we step almost immediately so that we can continue walking. This flexibility comes at a cost since we initially must pay deliberate attention to how we are moving. Gradually, after a few minutes on the trail, stepping becomes automatic so that we do not need to think about our movements. Canonical theory indicates that different adaptive motor learning mechanisms confer these essential properties to movement: explicit control confers flexibility, while forward model recalibration confers automaticity. Here we uncover a distinct mechanism of treadmill walking adaptation – an automatic stimulus-response mapping - that confers both properties to movement. The mechanism is flexible as it learns stepping patterns that can be rapidly changed to suit a range of treadmill configurations. It is also automatic as it can operate without deliberate control or explicit awareness by the participants. Our findings reveal a tandem architecture of forward model recalibration and automatic stimulus-response mapping mechanisms for walking, reconciling different findings of motor adaptation and perceptual realignment.

## Introduction

Flexibility and automaticity are essential features of human movement, so much so that we rarely think about the details of how we move. While walking, we do not think about the bend of the ankle or how quickly to swing the leg forward to step. Yet, we easily adjust walking to accommodate many situations – a muddy trail, a grassy slope, or a snowy path. These abilities are often taken for granted until something goes awry – a sprained ankle quickly brings the details of movement execution to awareness and deliberate control is often used to avoid pain or further injury. It is only then that we truly appreciate how much the sensorimotor system is doing without our conscious awareness.

We currently do not understand how different learning mechanisms confer both flexibility and automaticity to human movement. One well-studied process of motor learning is sensorimotor adaptation, which occurs in response to errors and helps adjust motor commands to correct for the perturbations to our movements that are introduced by the changed environmental demands (Bastian, 2008; Huberdeau et al., 2015). Adaptation is traditionally thought to rely on the cerebellar-dependent recalibration of a forward model that associates motor commands with expected sensory consequences (Ito, 1989; Wolpert et al., 2001). For example, to walk on an icy sidewalk, we may need to recalibrate our prediction of how firmly our feet will grip the ground. The process of forward model recalibration is automatic and implicit, as it has been shown to operate without intention or awareness (Huberdeau et al., 2015; Taylor et al., 2014). However, forward model recalibration is not as flexible because it cannot make immediate changes in movement – it can only adjust movement gradually trial-by-trial or step-by-step, a process that can take several minutes (Bastian, 2008; Huberdeau et al., 2015). For this reason, forward model recalibration leads to lasting movement errors (called “aftereffects”) when the environment returns to its original state after adaptation, as the newly acquired sensorimotor recalibration must be *unlearned* over time to restore a normal movement (Bastian, 2008; Martin et al., 1996).

Adaptation can rely on more than forward model recalibration. For example, adaptation of reaching movements consistently involves the use of explicit strategies, where people deliberately change where they are aiming to reach (Taylor et al., 2014; Taylor and Ivry, 2011). It can also involve memory-based caching (Huberdeau et al., 2019; McDougle and Taylor, 2019), where people learn motor responses in association with the respective environmental sensory stimuli, and cache them in memory for future retrieval, as in a lookup table. Finally, adaptation of reaching movements can involve structural learning, where people learn the general relationships or equations that link environmental sensory stimuli and appropriate motor responses, and later use them to compute motor responses anew (Bond and Taylor, 2017; Braun et al., 2009). Aiming strategies, memory-based caching, and structural learning differ from forward model recalibration in that they do not lead to aftereffects (Huberdeau et al., 2015; Taylor et al., 2014; Taylor and Ivry, 2011). This is thought to be because the motor responses are mapped to sensory information about the environment and are therefore flexible – they can be promptly abandoned or changed for different environmental stimuli. Hence, these mechanisms are collectively called “stimulus-response mapping mechanisms”. In reaching, stimulus-response mapping mechanisms do not operate automatically, and rely instead on explicit control (Bond and Taylor, 2017; Huberdeau et al., 2019; Taylor and Ivry, 2011). However, explicit control is poorly suited for automatic, continuous movements like walking (Clark, 2015; Paul et al., 2005) and can even interfere with them (Uiga et al., 2020; Wong et al., 2008). Unlike discrete reaching, individuals do not consciously aim their feet while engaged in continuous walking on surfaces that are typically encountered (e.g. floors, sidewalks) (Long et al., 2016; Roemmich et al., 2016). For these reasons, we speculated that other stimulus-response mapping mechanisms that are automatic and not explicit may contribute to walking adaptation.

Studying the automatic mechanisms of walking adaptation has proven challenging, because it is still unknown how to measure each mechanism in isolation. Here, we propose leveraging the phenomenon known as “perceptual realignment” to dissect the mechanisms underlying walking adaptation. When we adapt, we learn not only a new *motor* calibration, but also a new *perceptual* calibration of the action we are performing (‘t Hart and Henriques, 2016; Haith et al., 2008; Jensen et al., 1998; Vazquez et al., 2015). For example, as people adapt their reaching movements, they also realign their perception of movement direction or force production (‘t Hart and Henriques, 2016; Haith et al., 2008). Similarly, as people adapt their walking movements on a split-belt treadmill (one foot made to move faster than the other), they also realign their perception of leg speed (Jensen et al., 1998; Kristan A. Leech et al., 2018; Vazquez et al., 2015). Despite suggestions that perceptual realignment may also stem from recalibration of a forward model (‘t Hart and Henriques, 2016; Izawa et al., 2012; Rossi et al., 2021a; Synofzik et al., 2008), we do not yet know whether *the same* forward model recalibrates both movement and perception in parallel. It remains unclear whether there exists a direct relationship between perceptual realignment and the portion of motor adaptation achieved specifically by a mechanism of forward model recalibration. Establishing such a relationship would provide compelling evidence that perceptual realignment can be used as a measure of the forward model recalibration mechanism of motor adaptation. In turn, this would allow us to quantify the contribution of other, stimulus-response mapping mechanisms to adaptation.

In this study, we aimed to identify the learning mechanisms involved in split-belt treadmill walking adaptation. In Experiment 1, we uncovered a stimulus-response mapping mechanism that contributes to walking adaptation. We also isolated forward model recalibration and show that it changes movement and perception together. In Experiment 2, we found that the stimulus-response mapping mechanism is automatic and not under deliberate or explicit control; rather, it operates via either memory-based caching or structural learning.

## Results

### Experiment 1

We asked whether walking adaptation involves both forward model recalibration and a stimulus-response mapping mechanism learned in tandem. We hypothesized that we could assess this by carefully studying the motor and perceptual aftereffects that occur after split-belt walking. Note that “Recalibration” and “mapping” are at times used as shorthand for “forward model recalibration” and “stimulus-response mapping”.

#### Motor paradigm and hypotheses

We used established motor measurements to quantify split-belt walking adaptation (Reisman et al., 2005) (Finley et al., 2015). The “Perturbation” measures the effect of the split-belt treadmill on the stepping pattern — the total movement error we would see in the absence of adaptation. “Δ Motor output” measures the extent that individuals compensate for the perturbation by changing their stepping pattern — how much they alter when (time) and where (position) they step on the treadmill with each foot. “Step length asymmetry” measures the remaining movement error — the difference between the perturbation and Δ motor output (see Methods).

**Figure 1.**
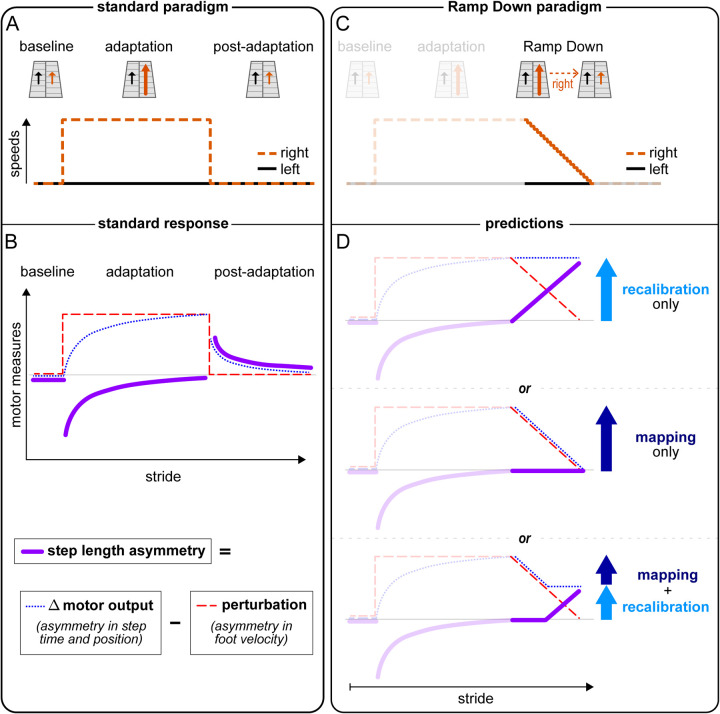
Experiment 1, Hypotheses and Predictions. **(A-B)** Treadmill belt speeds for the standard split-belt paradigm (A) and schematic time course of standard motor measures of walking adaptation (B): step length asymmetry – a measure of error (solid purple), Δ motor output – a measure of compensatory spatial and temporal asymmetries (dotted blue), and perturbation – the effect of the speed asymmetry on the walking pattern (dashed red). In baseline, the belts are tied, and perturbation, Δ motor output, and step length asymmetry are all ~0. In adaptation, the right leg is faster than the left such that the perturbation is positive. The Δ motor output is still ~0 in early adaptation, causing step length asymmetry errors (negative values). By late adaptation the Δ motor output is adapted to match the perturbation, and step length asymmetry returns to ~0. Changes to Δ motor output persist in tied-belts post-adaptation, but the perturbation is ~0, causing step length asymmetry aftereffects (positive error values, smaller magnitude than early adaptation). **(C)** Schematic of our paradigm with the Ramp Down task: after adaptation, the right belt speed is gradually ramped down to match the left. **(D)** Predictions for the Ramp Down motor measures made by three competing hypotheses. **Recalibration only:** changes to Δ motor output by recalibration are fixed, predicting that step length asymmetry aftereffects will emerge immediately as the perturbation decreases. **Mapping only:** changes by mapping are flexible and are predicted to track the perturbation, with no step length asymmetry aftereffect. **Recalibration + mapping:** changes involving both recalibration and mapping predict an intermediate result. At first, changes by mapping would be gradually abandoned to track the perturbation, such that step length asymmetry would remain zero. When only recalibration-related changes are left, the Δ motor output would be fixed and step length asymmetry aftereffects would begin to emerge. Aftereffect magnitude on tied belts would be smaller than the initial adaptation asymmetry.

Figure 1A illustrates the standard paradigm used in studies of split-belt adaptation, with abrupt transitions between tied-belt to split-belt phases, and Figure 1B illustrates the standard motor response. In the “baseline” phase, the perturbation, Δ motor output, and step length asymmetry are zero reflecting equal belt speeds and symmetric walking (red, blue, purple, respectively). In the “adaptation” phase, the belt speeds are different and the perturbation is positive. Initially, this leads to movement errors observed as the negative step length asymmetry. The Δ motor output gradually adapts to compensate for the perturbation, so that the step length asymmetry returns to zero. In “post-adaptation”, the belt speeds are tied and the perturbation is again zero. Individuals exhibit initial movement errors called “aftereffects”: the Δ motor output mismatches the perturbation (it remains elevated) and step length asymmetry is positive. Importantly, these aftereffects are smaller than the total motor learning: the Δ motor output is *less* elevated than in late adaptation, and the step length asymmetry magnitude is *smaller* than in initial adaptation (Kristan A. Leech et al., 2018; Rossi et al., 2021a, 2019; Statton et al., 2018).

Why are aftereffects smaller than the perturbation that was learned? We hypothesize that people may learn to compensate for the perturbation using a combination of forward model recalibration and stimulus-response mapping mechanisms. The key distinction between these mechanisms is that forward model recalibration leads to aftereffects (Bastian, 2008; Martin et al., 1996; Reisman et al., 2010), while stimulus-response mapping mechanisms do not (Huberdeau et al., 2015; Taylor et al., 2014; Taylor and Ivry, 2011) (SI Appendix, Experiment 1 mechanisms characteristics and Fig. S1). Hence, this hypothesis would account for the consistent finding that aftereffects are “partial” even when motor learning is “complete”, because only the portion learnt by recalibration would lead to aftereffects.

Standard paradigms with abrupt transitions from adaptation to post-adaptation phases are insufficient for assessing the hypothesis that people learn through both forward model recalibration and stimulus response mapping. This is because aftereffects on tied-belts may inaccurately appear “partial” due to testing methodologies (e.g., unlearning or forgetting due to momentary stops in the treadmill between different testing conditions) (Malone and Bastian, 2016), leaving open the possibility that people exclusively learn through forward model recalibration.

To obtain a robust measure of the aftereffects, we devised a “Ramp Down” paradigm with a gradual transition from adaptation to post-adaptation. Specifically, the right belt speed was ramped down for ~80 seconds until it matched the left belt speed (Fig. 1C), so that the perturbation returned to zero gradually. We asked whether participants exhibit aftereffects (Δ motor output mismatching perturbation, and positive step length asymmetry) for these intermediate perturbations. No aftereffects for a range of perturbations would be indicative of a stimulus-response mapping mechanism, because only Δ motor outputs learnt by mapping can flexibly change to match a range of perturbations.

We used this paradigm to whether adaptation is achieved by 1) forward model recalibration only, 2) stimulus-response mapping only, or 3) a combination of the two mechanisms. The three competing hypotheses make different predictions about when in the Ramp Down the aftereffects should emerge (Fig. 1D).

If adaptation involves forward model recalibration only, aftereffects should emerge immediately in the Ramp Down. The Δ motor output would remain elevated, because adjustments would take a longer time to change. Hence, Δ motor output (blue) would immediately diverge from the perturbation (red) and step length asymmetry (purple) would immediately grow (Fig. 1D top; also see SI Appendix, Fig. S1B).

If adaptation involves stimulus-response mapping only, there would be no aftereffects during the Ramp Down. Specifically, the Δ motor output would match the perturbation throughout the task, because adjustments to the Δ motor output by mapping are flexible and can be promptly scaled down. In turn, step length asymmetry would remain zero (Fig. 1D middle).

If adaptation involves both recalibration and mapping, aftereffects should emerge at some mid-point during the Ramp Down. The Δ motor output would initially match the perturbation and step length asymmetry would remain zero (Fig. 1D bottom). At some point in the ramp down, Δ motor output would diverge from the perturbation and step length asymmetry would begin to grow. This occurs when adjustments to the Δ motor output by mapping are zero, and adjustments by recalibration remain elevated (Fig. 1D bottom, second ~half of the Ramp Down task).

#### Motor results – step length asymmetry

**Figure 2.**
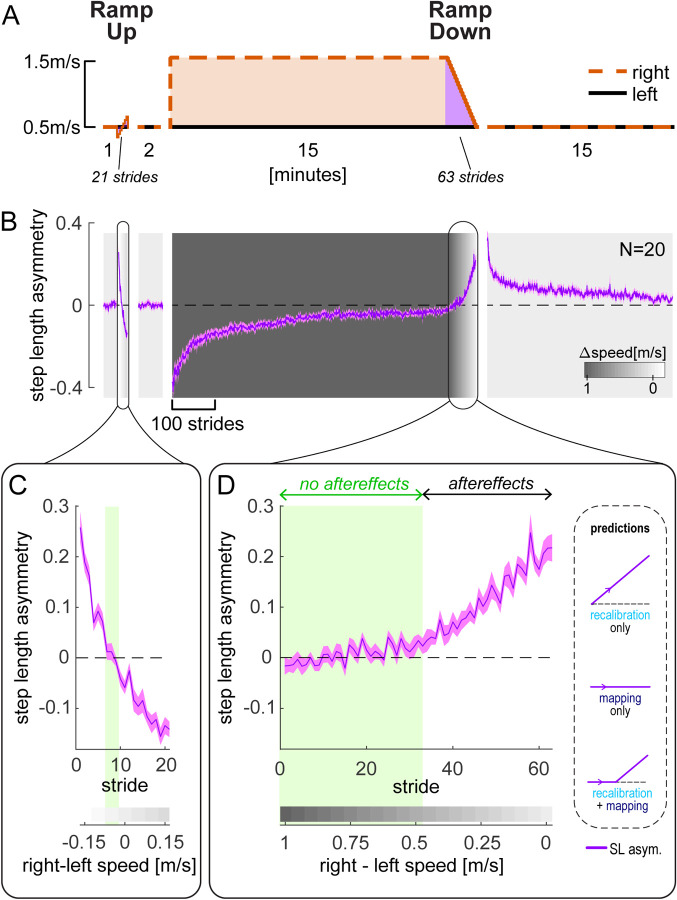
Experiment 1, Step length asymmetry. **(A)** Experimental protocol. The Ramp Down task (purple) is that used for predictions in Fig. 1D. **(B)** Step length asymmetry time course. Background shading darkness increases with belt speed difference (color bar). Phases (except Ramp tasks) are truncated to the participant with fewest strides. **(C-D)** Zoomed-in Ramp Up (C, baseline) and Ramp Down (D, post-adaptation) tasks. Speed differences for which step length asymmetry is not significantly different from zero are indicated by the green shade. Inset depicts predictions made by the competing hypotheses as in Fig. 1D. All curves show group mean ± SE.

The paradigm used for Experiment 1 is shown in Figure 2A, with the time course of step length asymmetry (group mean ± SE) in Figure 2B. The Ramp Down task corresponds to the manipulation illustrated in Figure 1C and is the focus of our analysis for Experiment 1. A similar but shorter Ramp Up task was performed prior to adaptation as a control to see how participants responded to a gradually ramped perturbation at baseline. Participants performed perceptual tests during the Ramp Up and Ramp Down tasks, which will be discussed in a later section (see “*Perceptual results”*). Patterns of step length asymmetry during other portions of the paradigm was consistent with a large body of previous work (e.g., (Kristan A Leech et al., 2018; Kristan A. Leech et al., 2018; Reisman et al., 2005; Roemmich et al., 2016; Rossi et al., 2019; Vazquez et al., 2015)) and will not be discussed further.

Step length asymmetry during the baseline Ramp Up and post-adaptation Ramp Down tasks are displayed in Figure 2C–D. For each speed configuration in the Ramp Down task, we statistically compared step length asymmetry to zero. We evaluated the emergence of aftereffects by looking for when step length asymmetry became significantly positive. We found that step length asymmetry was *not* statistically different from zero during the first half of the Ramp Down task, for right belt speeds ranging from 1m/s to 0.5m/s faster than the left speed (these speed configurations are highlighted in green in Fig. 2D; all CI_LB_ (confidence intervals lower bounds) ≤ −0.001 and CI_UB_ (upper bounds) ≥ 0.009), SI Appendix, Fig. S2 and Table S1). This result indicates that aftereffects do not emerge immediately in the Ramp Down task, which negates the recalibration only prediction (Fig. 2D inset). Step length asymmetry was instead significantly positive for the second half of the task, for right belt speeds ranging from 0.45m/s to 0m/s faster than the left speed (all CI_LB_ > 0.01). These results indicate that aftereffects emerge at a mid-point during the Ramp Down task and align with behavioral predictions from the recalibration + mapping hypothesis (Fig. 2D inset).

As a control, we repeated the same analysis for the baseline Ramp Up task (Fig. 2C). We found that there was only one speed configuration for which step length asymmetry was not statistically different from zero (right speed 0.05m/s slower than left; SL asym. = 0.006 [−0.017, 0.028], mean [CI]). For all other configurations, step length asymmetry was significantly positive (CI_LB_ ≥ 0.048 for right speed 0.1 to 0.35m/s slower than left) or negative (CI_UB_ ≤ −0.019 for right speed 0 to 0.35m/s faster than left). This is in stark contrast to the wide range of speed configurations with near-zero step length asymmetry observed in the Ramp Down period. Hence, the ability to walk symmetrically at different speed configurations is not innate but dependent on the adaptation process, corroborating the hypothesis that the “no aftereffects” region of the Ramp Down reflects the operation of a mapping mechanism.

#### Motor results – perturbation and Δ motor output

**Figure 3.**
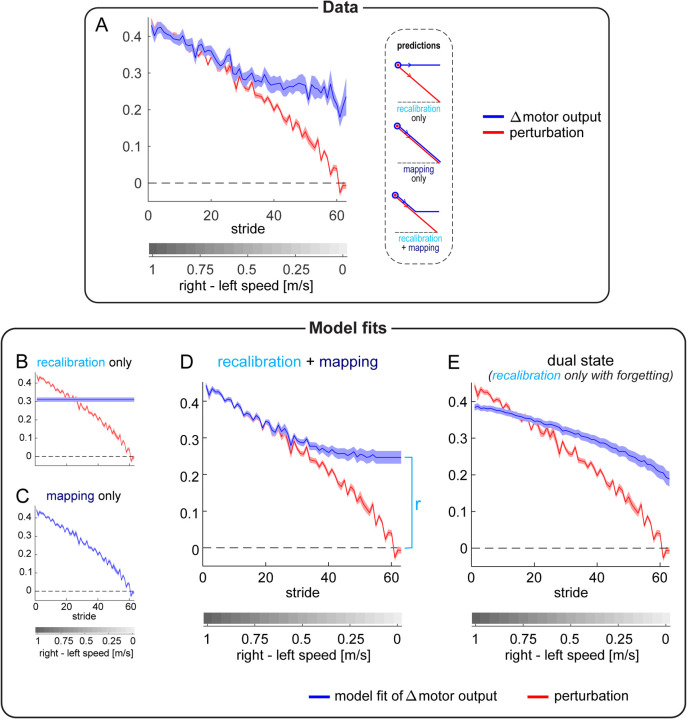
Experiment 1, Perturbation and Δ motor output. **(A)** Perturbation (red) and Δ motor output (blue) data for the Ramp Down task. Inset depicts predictions made by the competing hypotheses as in Fig. 1D. **(B-E)** Perturbation data (red) and model fit for the Δ motor output (blue) for recalibration only, mapping only, recalibration + mapping, and dual state models. The blue bracket in (D) points to the value on the y-axis of the model parameter “r”. All curves show group mean ± SE.

To further test the competing hypotheses, we examined the perturbation and Δ motor output during the Ramp Down (Figure 3A). As the belt speed difference decreased, so did the perturbation component (red). In the first half of the task, the Δ motor output (blue) appeared to match the perturbation – consistent with the lack of step length asymmetry aftereffects. Additionally, in the second half of the task, the Δ motor output appeared larger than the perturbation – consistent with the positive step length asymmetry aftereffects observed in Fig. 2D.

We formally contrasted predictions made by the competing hypotheses by developing mathematical models of the Δ motor output as a function of perturbation. In the simplest framework, the Δ motor output behavior illustrated in Figure 1D can be formalized as follows:

(1)  Forward Model Recalibration:u(p)=r

(2)  Stimulus-Response Mapping:u(p)=p

(3) $$\text{ Recalibration + Mapping}:\;u(p)=\{\begin{array}{c} p,\text{for }p\geq r\\ r,\text{otherwise } \end{array}$$

Where *u* is the modelled Δ motor output, *p* is the perturbation, and *r* is a free parameter representing the portion of the Δ motor output related to recalibration. The recalibration + mapping model describes the scenario where participants modulate the Δ motor output to match the perturbation (*u*(*p*) = *p*) for the first portion of the Ramp Down task. The Δ motor output remains constant during the second portion of the task (*u*(*p*) = *r*) because participants are unable to reduce the Δ motor output to values smaller than “*r* ” (the amount achieved by recalibration). Parameters were estimated by fitting each model to individual participants’ Δ motor output data from the Ramp Down (see Methods).

The fits of the Ramp Down Δ motor output for the recalibration only, mapping only, and recalibration + mapping models are displayed in Figures 3B, C, and D, respectively (mean ± SE of fits across participants; individual fits for recalibration + mapping are shown in SI Appendix, Fig. S3). As expected, only the recalibration + mapping fit captured the matching-then-divergent behavior of Δ motor output in response to the changing perturbation. The BIC statistic revealed that the recalibration + mapping function explained the data better than either the recalibration only or mapping only functions (BIC differences: 36.171 [24.868, 47.901] and 58.698 [43.708, 74.215], mean [CI]).

We additionally considered the dual state model of motor adaptation from Smith et al. (Smith et al., 2006) as an additional framework to represent the recalibration only hypothesis (see Methods and SI Appendix, Fig. S1B). In contrast to the simpler recalibration only model illustrated above, the dual state model has four parameters and can account for potential forgetting or unlearning of the Δ motor output that may occur during the Ramp Down. It can also account for the possibility that adaptation involves two recalibration mechanisms – i.e., two distinct mechanisms that both learn via a process of forward model recalibration and both contribute to aftereffects, but that learn and forget at different rates. We chose this model because it is a well-established model that is widely used to capture the Δ motor output time-course for traditional motor adaptation paradigms (i.e., those consisting of adaptation and post-adaptation phases). Yet, performance of this model for a manipulation like the Ramp Down task performed here has not yet been tested. As such, if the recalibration + mapping model fit the Ramp Down data better than the dual state model, this would provide robust evidence for the presence of a stimulus-response mapping mechanism.

We show the Δ motor output fit by the dual state model in Figure 3E (group mean ± SE; individual fits are shown in SI Appendix, Fig. S4). Similar to its simpler recalibration only model counterpart, the dual state model was not able to capture the matching-then-divergent behavior of Δ motor output. The BIC statistic confirmed that the recalibration + mapping model fitted the data significantly better than the dual state (BIC difference = 8.455 [3.557, 13.597], mean [CI]). In sum, the modeling analysis of the Δ motor output further support the recalibration + mapping hypothesis.

#### Perceptual test and results

The second goal of Experiment 1 was to evaluate the hypothesis that “perceptual realignment” (a phenomenon leading to altered perception following adaptation) results from the operation of the same forward model recalibration mechanism involved in adaptation of the Δ motor output (Rossi et al., 2021a).

Previous work shows that perception realigns following gait adaptation: after adapting to a perturbation where the right treadmill belt is *faster* than the left, return to tied belts results in perception of the opposite asymmetry (i.e., right speed feels *slower* than left) (Jensen et al., 1998; Vazquez et al., 2015). We therefore expected that people would perceive the belt speeds as equal at some point in the Ramp Down and eventually perceive the opposite asymmetry on tied belts. We measured this by asking participants to press a keyboard button at two separate occasions during the Ramp Down task: (1) when the belts first felt equal, and (2) when they no longer felt equal (see Methods).

**Figure 4.**
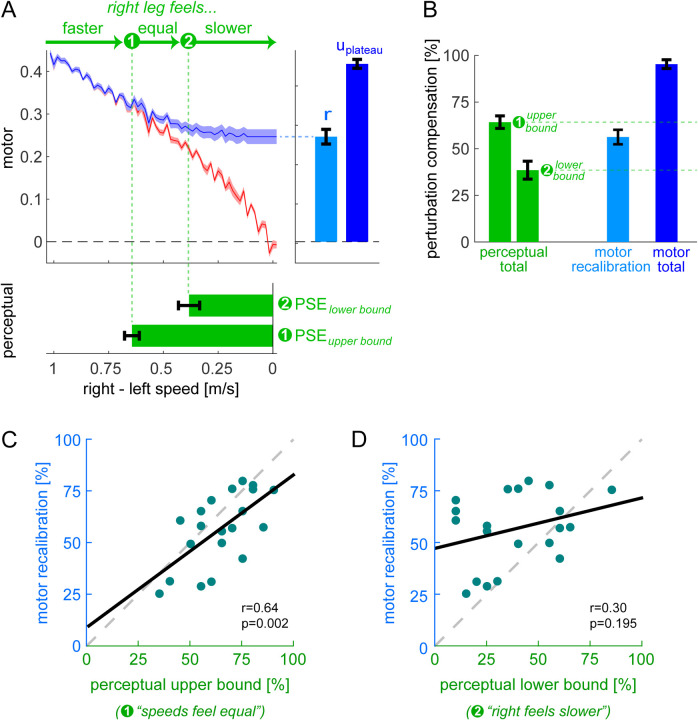
Experiment 1, Perceptual Results. **(A)** Top: perturbation data (red) and recalibration + mapping fit (blue). Bottom: perceptual task button presses (green, group mean ± SE), as a function of belt speed difference. Right: measures of motor recalibration (“*r*”) and total motor adaptation (“ *u*_plateau_ ”). **(B)** Perturbation compensation (normalized perceptual and motor measures of adaptation): *compensation*_perceptual_ bounds (green), *compensation*_motor total_ (dark blue), and *compensation*_motor recalibration_ (light blue). **(C-D)** Individual participants’ *compensation*_motor recalibration_ versus *compensation*_perceptual_ (first or second button press). Solid black: least squares line. Dashed gray: unity line.

Figure 4A depicts the button presses for the Ramp Down perceptual test (top panel, group mean) overlayed onto the Ramp Down motor data (recalibration + mapping fit). Note that the task captures a *range* of belt speed configurations that participants perceive as “equal speeds”, with button presses (1) and (2) corresponding to the upper and lower bounds of this range. This is expected because perception is known to be noisy and may not be sensitive enough to discriminate between belt speed configurations that are too similar.

We quantified perceptual realignment using the established measure of *point of subjective equality* (PSE), defined as the belt speed difference perceived as “equal speeds”. A PSE of zero would indicate no perceptual realignment (accurate perception) and a PSE of 1m/s (i.e., a magnitude equivalent to the difference between the belt speeds during adaptation) would indicate complete perceptual realignment such that the belt speeds feel equal during the adaptation phase.

We measured belt speed difference at the time of each button press. We computed PSE as the range of belt speed difference values between these two measurements:

(4) $$PSE_{\text{upper bound }}={\left[\text{right}\;\text{belt}\;\text{speed}-\text{left}\;\text{belt}\;\text{speed}\right]}_{\text{time of button press }1}$$

(5) $$PSE_{\text{lower bound }}={\left[\text{right}\;\text{belt}\;\text{speed}-\text{left}\;\text{belt}\;\text{speed}\right]}_{\text{time of button press }2}$$

We found that *PSE*_upper bound_ was 0.64±0.03m/s and *PSE*_lower bound_ was 0.39±0.05m/s (Figure 4A, bottom panel, group mean ± SE). This was consistent with previous work (Kristan A. Leech et al., 2018). This perceptual realignment was not present during baseline testing, as illustrated in SI Appendix, Fig. S5.

We aimed to evaluate the hypothesis that perceptual realignment arises from the forward model mechanism of motor adaptation and is unaffected by the stimulus-response mapping mechanism. This hypothesis predicts that the extent of perceptual realignment should be: 1) ~equal to the extent of motor adaptation achieved by recalibration, and 2) less than the total extent of motor adaptation (which also includes mapping). We quantified 1) motor adaptation by recalibration as the fitted parameter “*r*” from the recalibration + mapping model, and 2) total motor adaptation as the Δ motor output at adaptation plateau (Fig. 4A, right panel, see previous section “Motor results – perturbation and Δ motor output). We expressed these motor measures and perceptual realignment as “percent compensation for the perturbation” (i.e., normalized to the perturbation magnitude in the respective units) so that they could be compared:

(6) $$\;\text{compensation}{\;}_{\text{perceptual }}=\frac{PSE}{1\text{m}/\text{s}}$$

(7) $$\;\text{compensation}{\;}_{\text{motor recalibration }}=\frac{r}{p_{\text{plateau }}}$$

(8) $$\text{compensation}{\;}_{\text{motor total }}=\frac{u_{\text{plateau }}}{p_{\text{plateau }}}$$

Where *r* is the fitted parameter from the recalibration + mapping model, *p*_plateau_ is the mean perturbation over the last 30 strides of adaptation, and *u*_plateau_ is the mean Δ motor output over the last 30 strides of adaptation (for Eq. 6, note that 1m/s is the belt speed difference in adaptation).

We show group level compensation measures in Fig. 4B. We found that *compensation*_motor recalibration_ (56±4%, group mean ± SE) fell within the *compensation*_perceptual_ range (39±5% to 64±3%): it was significantly smaller than the upper bound (difference = −8 [−14, −2]%) and significantly larger than the lower bound (difference = 18 [8, 28]%, mean [CI]). This supports our first prediction that perceptual realignment is comparable to the extent of motor adaptation achieved by recalibration. Furthermore, *compensation*_perceptual_ was significantly smaller than *compensation*_motor total_ (95±2%, mean ± SE; difference = 31 [25, 38]% or 57 [48, 66]%, upper or lower perceptual bounds, mean [CI]). This supports our second prediction that perceptual realignment is less than the total extent of motor adaptation.

We show individual *compensation*_motor recalibration_ and *compensation*_perceptual_ measures for each participant in Figure 4C–D. We evaluated Pearson’s correlation coefficients between these measures to test whether there is a direct relationship between perceptual realignment and the motor adaptation achieved by recalibration. We found that *compensation*_motor recalibration_ was significantly correlated with the upper bound of *compensation*_perceptual_ – the value computed using the “speeds feel equal” button press (r=0.64, p=0.002). This supports the hypothesized relationship between motor recalibration and changes to leg speed perception. Instead, *compensation*_motor recalibration_ was not correlated with the lower bound of *compensation*_perceptual_ (r=0.30, p=0.195) – this value is computed using the “right feels slower than left” button press, suggesting the true PSE may lay closer to the first button press. Together, our results support the hypothesis that perceptual realignment can be used as a proxy measure for the extent of motor adaptation achieved by forward model recalibration.

#### Control experiments

We performed six control experiments and reanalyzed previously-published data (Kristan A. Leech et al., 2018) to replicate the findings of Experiment 1 across different paradigm conditions (SI Appendix, Control experiments, Fig. S6–8, and Table S2–6). This provided additional support for the recalibration + mapping hypothesis. We first checked that the motor and perceptual behaviors observed in the Ramp Down could be replicated using a different method of assessment. A “speed match” task was given where participants use a joystick to increase the speed of the right belt (initially stationary) until they feel it matches the left belt (SI Appendix, Fig. S6B, “Ascend”) (Kristan A. Leech et al., 2018; Rossi et al., 2019; Statton et al., 2018; Vazquez et al., 2015). This approximates the second half of the Ramp Down (Fig. 4A, right leg feels equal or slower than left). Consistent with this, we observed step length asymmetry aftereffects early in the speed match task (initial SL asym. = 0.433 [0.271, 0.612], mean [CI]). Asymmetry decreased and was eventually ~0 when the belt speeds felt equal (final SL asym. = 0.002 [−0.040, 0.045]; SI Appendix, Fig. S7B).

We also used a “descend” speed match task where participants *decreased* the speed of the right belt (initially fast as in adaptation) until they felt it matched the left (SI Appendix, Fig. S6B). This approximated the first half of the Ramp Down. As expected, step length asymmetry was close to zero for entire task (initial: −0.017 [−0.069, 0.039], final: 0.043 [−0.0004, 0.090], mean [CI]; SI Appendix, Fig. S7B). The PSE for both speed match groups was comparable to that of Experiment 1 (*compensation*_perceptual_ difference between Ramp Down lower bound and ascend or descend Control = −3 [−16, 10]% or 4 [−12, 18]%, mean [CI]), and smaller than motor adaptation (*compensation*_motor total_ – *compensation*_perceptual_ = 48 [39, 57]% or 56 [42, 70]% for ascend or descend, mean [CI]; SI Appendix, Fig. S7A).

We replicated our findings in additional speed match experiments that varied in adaptation duration (3, 15, or 30 minutes), perturbation magnitude (1m/s or 0.4m/s speed difference) and schedule (abrupt or gradual) (SI Appendix, Control experiments and Fig. S6). Regardless of the paradigm, the step length asymmetry and PSE were consistent with the recalibration + mapping hypothesis (SI Appendix, Fig. S7 and Table S2–3).

### Experiment 2

In Experiment 2, we considered two hypotheses for how stimulus-response mapping may operate, that could both explain the results of Ramp Down task of Experiment 1. First, the mechanism may be a memory-based mapping that caches prior Δ motor outputs in memory, and later retrieves them in response to a matching perturbation (Huberdeau et al., 2019; McDougle and Taylor, 2019; Poggio and Bizzi, 2004; Wolpert et al., 2001). In adaptation, the Δ motor output changes gradually from zero to a value matching the perturbation (see Fig. 1B), and all intermediate values may be stored in memory. Therefore, a memory-based mapping can produce Δ motor outputs that match perturbations *smaller* than the adaptation perturbation (Kristan A Leech et al., 2018) – such as those of the Ramp Down task of Experiment 1. However, a memory-based mapping would not be able to account for perturbations *larger* than the adaptation perturbation: the largest Δ motor output available in memory would still undershoot such perturbations, resulting in negative step length asymmetry.

Alternatively, the mechanism may be a structure-based mapping that learns the algorithm to compute Δ motor outputs from sensory stimuli (Bond and Taylor, 2017; Braun et al., 2009; McDougle and Taylor, 2019; Wolpert et al., 2001). In adaptation, the mechanism may learn the relationship between perturbation and appropriate Δ motor output, and later use it to generate Δ motor outputs anew. Therefore, a structure-based mechanism can also *extrapolate* Δ motor outputs beyond what was experienced in adaptation, accounting for perturbations *larger* than the adaptation perturbation.

**Figure 5.**
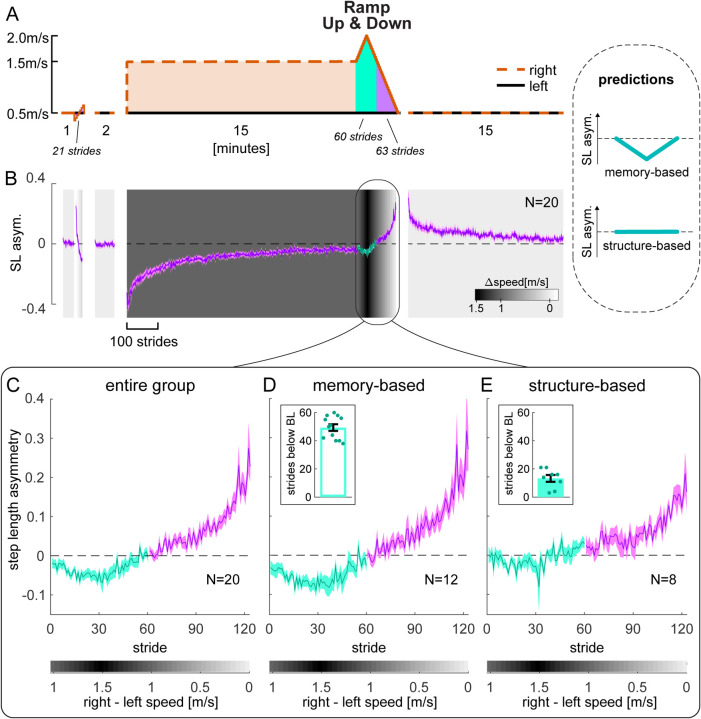
Experiment 2, Step length asymmetry. **(A)** Experimental protocol, equivalent to that of Experiment 1 except for the Ramp Up & Down part shaded in teal, where the right speed was faster than in adaptation (ramped up to 2m/s and back down to 1.5m/s). **Inset:** Predictions for the step length asymmetry during the teal portion of the Ramp Up & Down task, for the memory-based (top) or structure-based (bottom) mapping hypotheses. **(B)** step length asymmetry time course (entire group mean ± SE). Background shade represents belt speed difference. Phases (except Ramp tasks) are truncated to the participant with fewest strides. **(C)** Zoomed-in Ramp Up & Down task (entire group mean ± SE). Step length asymmetry for strides taken at right speeds larger than adaptation is shown in teal. **(D-E)** Separate plots of the step length asymmetry in the Ramp Up & Down task for the subgroups of participants that walked asymmetrically (D, “memory-based”) versus symmetrically (E, “structure-based”) in the teal portion of the task (subgroups mean ± SE). **Insets:** circles represent individual participants’ number of strides, in the teal portion of the task, with step length asymmetry below their own baseline CI. Error bars depict subgroup mean ± SE. Subgroup assignment was performed by clustering on this measure.

Thus, we tested a Ramp Up & Down condition where the speed of the right belt was both gradually increased and then decreased after adaptation (Fig. 5A). The memory-based hypothesis predicts that step length asymmetry will become negative for perturbations *larger* than adaptation, while the structure-based hypothesis predicts it will remain close to zero (Fig. 5A inset, “predictions”). The rest of the paradigm was analogous to that of Experiment 1 except there was no perceptual assessment.

Figure 5B shows step length asymmetry throughout the paradigm, and Figure 5C shows a close-up of the Ramp Up & Down task performance (group mean ± SE). On average, participants’ step length asymmetry patterns did not remain zero for speed differences larger than adaptation (teal). However, we observed that individual participants exhibited markedly different patterns of step length asymmetry during this phase (SI Appendix, Fig. S9). We quantified this observation by evaluating, for each participant, the number of strides in this phase with step length asymmetry below their own baseline CI (SI Appendix, Table S7). We used a density-based analysis to formally assess whether there were separate clusters in our data (see Methods). Indeed, the algorithm detected two separate clusters of participants: for 12 participants, between 38 to 60 strides were asymmetric (out of 60 total strides); for the other 8 participants, only 3 to 21 strides were asymmetric (Fig. 5D–E insets, and SI Appendix Fig. S10; difference in strides between subgroups = 36.083 [29.917, 42.250], mean [CI]). As a control, we performed the same clustering analysis for Experiment 1, and did not find separate clusters for any of our measures of interest (SI Appendix, Experiment 1 clustering analysis, Fig. S10–11, and Table S8–9).

This result indicates that 12 of 20 participants could not account for belt speed differences larger than that of adaptation, suggesting that they used a memory-based mapping mechanism (Fig. 5D). In contrast, 8 of 20 participants could account for these speeds, suggesting that they engaged a structure-based mapping mechanism (Fig. 5E).

We next aimed to exclude the possibility that participants in the structure-based subgroup may simply be faster at adapting to new perturbations than those in the memory-based subgroup, and may be adapting to the new perturbations of the Ramp Up and Down rather than generating Δ motor output using a previously learnt structure. To this end, we evaluated learning rates during adaptation. We found that participants in the two subgroups adapted at similar rates (strides to plateau difference, structure – memory: 135.875 [−53.208, 329.708], mean [CI]; SI Appendix, Fig. S12–13), confirming that different pattern of step length asymmetry in the Ramp Up and Down task reflects different types of mapping in the subgroups.

We considered that the mapping adjustments described here may or may not be *deliberate* (i.e., participants are trying to correct for the perturbation) and done with an *explicit strategy* (i.e., participants can accurately report a relevant strategy that would counter the perturbation) (Long et al., 2016; Roemmich et al., 2016). An example of an explicit strategy in visuomotor reaching adaptation is when participants report that they aimed to offset a visual rotation (Bond and Taylor, 2017; Taylor et al., 2014); conceivable examples in split-belt treadmill adaptation may be reports of “taking steps of similar length”, “stepping further ahead with the right foot” or “standing on the left foot for longer”. We tested whether participants could explicitly report changes to the gait pattern that specifically correct for the split-belt perturbation.

At the end of the experiment, participants were asked to report (in writing) if/how they had changed the way they walked during adaptation (note that this was a later addition to the protocol and was only collected in 16 of the 20 participants). We assessed reports by categorizing them in three steps: 1) did the report mention *any* deliberate changes? 2) were the changes *relevant* to adaptation? (i.e., did the report mention any gait metric contributing to the overall Δ motor output or step length asymmetry adaptation in any amount?) 3) was the response *accurate?,* as participants often reported strategies that they did not actually execute.

**Figure 6.**
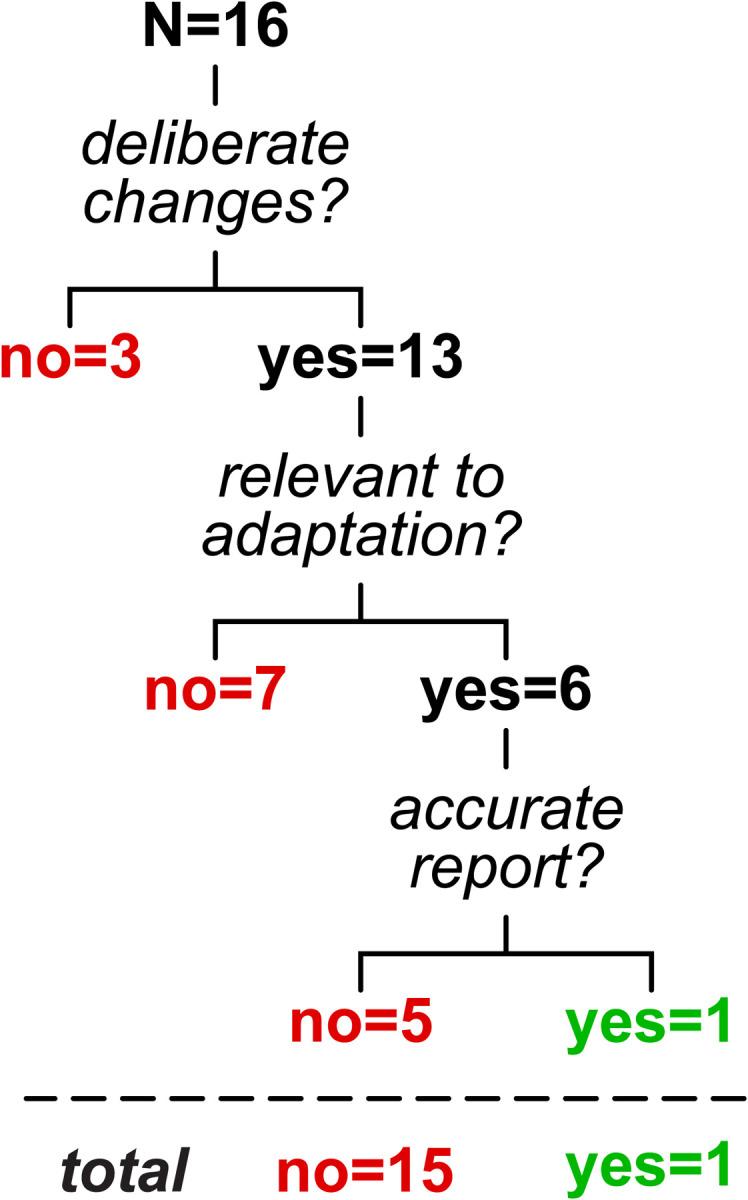
Summary of self-reported deliberate changes to the walking pattern in adaptation. Only one participant accurately described changes to the walking pattern that related to adaptation, while other responses were negative (i.e. no deliberate changes, 3 participants), irrelevant (7 participants), or inaccurate (5 participants).

We summarize results from the questionnaire in Figure 6 (original responses are reported in SI Appendix, Table S10). We found that, while 13 participants reported *deliberate* changes, only 6 people mentioned *relevant* aspects of the walking pattern (in particular, participants mentioned “limping” or temporal coordination). Furthermore, only one of these participants reported an *accurate* gait parameter (“I tried to spend as much time leaning on my left leg as possible”), while the remainder of the relevant responses were inaccurate (e.g., “matched duration of standing on each foot”) or vague (e.g., “I adjusted as if I was limping”). The aspect of gait most frequently reported across participants was stability (also reported as balance, not falling, or controlling sway). This suggests that, while participants may deliberately adjust their body to feel more stable, they do not seem to explicitly strategize how to offset the perturbation. Thus, mapping tends to adjust aspects of the walking pattern that participants are not explicitly aware of controlling, suggesting that this differs from explicit strategies often observed in reaching.

In sum, Experiment 2 suggests that the mapping mechanism involved in split-belt adaptation is memory-based in some people and structure-based in others. Neither mechanism seems to rely on explicit strategies.

## Discussion

**Figure 7.**
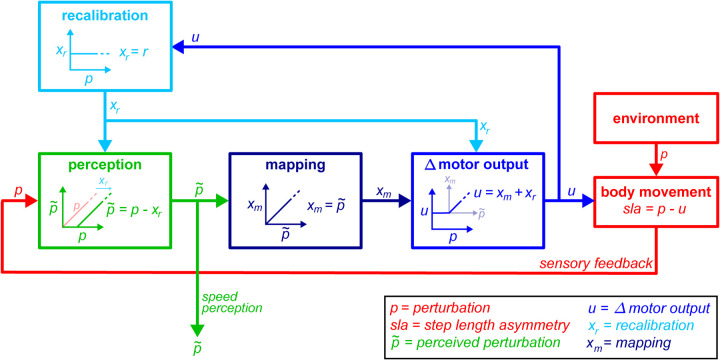
Schematic model of adaptation. Body movement depends on environment perturbations (red) and Δ motor output (blue). The Δ motor output is adjusted by recalibration (light blue) and mapping (dark blue) mechanisms, which perform different operations and are arranged in tandem. We propose the following architecture and flow: **(1. Recalibration)** The recalibration mechanism produces adjustment *x*_*r*_ that is fixed regardless of perturbation size (light blue box, *x*_*r*_ is constant for varying *p*). The *same* recalibration adjustment *x*_*r*_ serves as an input to *both* areas responsible for conscious perception (green box) and Δ motor output (blue box). **(2. Perception)** Conscious perception is computed by cancelling out the recalibration adjustment from the actual sensory feedback (green box, perception of the belt speed difference perturbation $\tilde{p}$ is the difference between the actual speed difference *p* and recalibration *x*_*r*_). The perceived perturbation $\tilde{p}$ serves as an input to the mapping mechanism (dark blue box). **(3. Mapping)** The mapping mechanism produces adjustment *x*_*m*_ that can vary in magnitude to appropriately account for the perceived perturbation $\tilde{p}$ (dark blue box, *x*_*m*_ scales with $\tilde{p}$ and matches its magnitude). **(4. Δ Motor output)** The overall adjustment to Δ motor output is computed by adding the mapping adjustment *x*_*m*_ and recalibration adjustment *x*_*r*_. The corner in the Δ motor output versus perturbation profile arises because mapping is computed based on the perceived perturbation $\tilde{p}$ (not the actual perturbation *p*) and is only learnt for positive $\tilde{p}$ (the experienced direction). When the perturbation is perceived to be opposite than adaptation, even if it is not, mapping is zero and the Δ motor output is constant, reflecting recalibration adjustments only (blue box, when $\tilde{p}<0$ and *p* ≥ 0 the mapping adjustment *x*_*m*_ is zero and *u* = *x*_*r*_).

In this study, we showed that locomotor adaptation involves two learning mechanisms: forward model recalibration and a stimulus-response mapping mechanism. The recalibration mechanism changes movement and relates to the perceptual changes observed in locomotor adaptation; the mapping mechanism is flexible and, once learned, can change our motor output immediately to account for a range of belt speed configurations. Our data suggest it can be memory-based or structure-based. We propose a comprehensive conceptual model where the same output from the forward model (Fig. 7, light blue) may project to both sensory integration and motor control areas (green and blue boxes), where it may be used to recalibrate perception and movement respectively. We also propose that the same *recalibrated perception* (green arrow) serves both as signal for conscious perception and as an input to the mapping mechanism (dark blue box), where it is used to compute Δ motor output adjustments that will be summed to the recalibration-related adjustments (Δ motor output, blue box).

### Forward model recalibration of movement and perception

Our results support the hypothesis that changes in movement perception with adaptation are also mediated by the recalibration of a forward model (‘t Hart and Henriques, 2016; Izawa et al., 2012; Sombric et al., 2019; Synofzik et al., 2008; Yavari et al., 2016) (for a review, see (Rossi et al., 2021a)). In line with a variety of previous studies (e.g., (Haith et al., 2008; Harris, 1963; Jensen et al., 1998; Moidell and Bedell, 1988; Sombric et al., 2019)), we find that people recalibrate their movement perception in adaptation and feel less perturbed than they really are – here, they feel that their right leg moves slower than its true speed (Jensen et al., 1998; Kristan A. Leech et al., 2018; Rossi et al., 2019; Statton et al., 2018; Vazquez et al., 2015). Consistent with the operation of a forward model thought to predict sensory consequences from efference copies (Haruno et al., 2001; Ito, 1989; Wolpert et al., 2001), perceptual changes are expressed during active and not passive movements (Sombric et al., 2019; Vazquez et al., 2015). Perceptual changes with adaptation are consistently affected by damage to (Izawa et al., 2012; Statton et al., 2018; Synofzik et al., 2008) or stimulation of the cerebellum (Yavari et al., 2016), the structure imputed with forward models. We suggest that perceptual changes may reflect cerebellar recalibration (Fig. 7, “recalibration”, light blue) processed downstream by cortical sensory areas (“perception”, green box). Indeed, activity in both cerebellum and parietal lobe is shown to increase with split-belt walking adaptation (Hinton et al., 2019).

We suggest participants may filter out the portion of the belt speed perturbation that is predictively accounted for by the forward model, leading to the perceptual changes observed here (Fig. 7, green box: the forward model recalibration, *x*_*r*_, is cancelled from the speed difference sensory signal, *p*, to produce the perceived speed difference, $\tilde{p}$). The parietal lobe receives input from the cerebellum (Judd et al., 2021; Sultan et al., 2012) and has the capacity to selectively cancel sensory prediction signals (Blakemore et al., 1998; Brooks and Cullen, 2019). This is important for filtering out self-generated sensory stimuli from those externally produced (Blakemore et al., 1998; Brooks and Cullen, 2019; Rondi-Reig et al., 2014), or predictable environmental stimuli from those that are novel (Anderson et al., 2012; Rondi-Reig et al., 2014). While we think this is a parsimonious explanation, cerebellar predictions may also be integrated with and sharpen proprioceptive estimates (Bhanpuri et al., 2013; Weeks et al., 2017), raising the possibility that a more complex integration of predicted and actual sensory signals contribute to perceptual changes.

We demonstrated that perceptual and motor recalibrations are directly related. Motor recalibration is approximately constant when the belt speed difference is changed after adaptation, as captured by the parameter “*r*”. We find “*r*” is correlated with the “belt speeds feel equal” button press, suggesting that output from the same forward model (Fig. 7, *x*_*r*_, light blue) may be used to change both movement (*u*, blue) and perception ($\tilde{p}$, green) during adaptation (SI Appendix, Supplementary Discussion 1). There is evidence to suggest that single regions of the cerebellar cortex (Pisano et al., 2021) and possibly single output cells from the cerebellar nuclei (Judd et al., 2021; Sultan et al., 2012) may project downstream to both motor and sensory cortical areas. Furthermore, the cerebellum has been shown to modulate activity in sensory and motor cortices in a coordinated manner (Lindeman et al., 2020; Popa et al., 2013), and both regions show evidence of sensory cancellation during active movement (Seki and Fetz, 2012). Hence, it is possible that the motor and perceptual recalibrations observed here reflect the differential processing of cerebellar output (Fig. 7, “recalibration”, light blue) by motor and sensory (blue and green, respectively) cortical areas.

### Automatic yet flexible stimulus-response mapping

We identified a stimulus-response mapping mechanism that does not conform with currently known mechanisms of walking adaptation. When an explicit goal is given, adaptation mechanisms deployed in addition to forward model recalibration are consistently found to operate under deliberate control (Codol et al., 2018; Taylor et al., 2014; Taylor and Ivry, 2011) – including memory-based caching (Huberdeau et al., 2019; McDougle and Taylor, 2019) and structural learning mechanisms (Bond and Taylor, 2017; McDougle and Taylor, 2019). Mounting evidence indicates that explicit strategies do not play a strong role in split-belt walking adaptation where an explicit movement goal is not provided to the participants; for example, people do not adapt faster even after watching someone else adapt (Song et al., 2020). Strategic adjustments to the walking pattern can be temporarily elicited by providing additional visual feedback of the legs and an explicit goal of how to step. Yet, these adjustments disappear immediately upon removal of the visual feedback (Roemmich et al., 2016), and have no effect on the underlying adaptive learning process (Long et al., 2016; Malone and Bastian, 2010; Roemmich et al., 2016). Furthermore, performing a secondary cognitive task during walking adaptation does not affect the amount of motor learning (Hinton et al., 2020; Malone and Bastian, 2010; Rossi et al., 2021b; Vervoort et al., 2019). Here, we confirmed that explicit strategies are not systematically used to adapt step length asymmetry and Δ motor output: only one person accurately reported making relevant changes appropriate to walk with symmetry; the rest either did not know what they did, reported changes that did not actually occur or would not lead symmetry (SI Appendix, Supplementary Discussion 2).

Despite not relying on explicit strategies, we find that the mapping mechanism scales down immediately to account for the amount of perturbation not corrected by recalibration. To the best of our knowledge, this is the first study to show that such flexible mechanism plays a role in walking adaptation. Previous work from our group used a manipulation that involved ramping down the speed of the right belt after adaptation (Roemmich and Bastian, 2015), similar to the Ramp Down used here. However, this was done slowly over 10 minutes, so that when participants walked with near-zero step length asymmetry for a portion of the ramp, this was attributed to them having unlearnt the adapted pattern. We here eliminated this confounder by ramping down the right speed quickly – on average, the ramp down took 1 minute and 20 seconds. Importantly, this time scale was short enough that participants’ aftereffect at the end of the ramp down was comparable to that observed in studies without the ramp down (e.g., (Reisman et al., 2005)). This manipulation allowed us to discover a sharp change in Δ motor output behavior half-way through the ramp down that supports the presence of a mapping mechanism. That is, the Δ motor output can rapidly change and match the perturbation but only for speed differences that are ~50–100% the adaptation speed difference, remaining mostly constant for smaller speed differences. Hence, the flexible behavior of the Δ motor output cannot be explained by the unlearning process, and it is instead indicative of the operation of a mapping mechanism.

As such, the mapping mechanism combines the advantages of automaticity and flexibility (Huberdeau et al., 2015; Taylor et al., 2014; Taylor and Ivry, 2011). This is ecologically important for both movement accuracy (Uiga et al., 2020; Wong et al., 2008) and for walking safely in real-world situations, where we walk while talking or doing other tasks, and terrains are uneven (Clark, 2015; Paul et al., 2005; Wong et al., 2008) (SI Appendix, Supplementary Discussion 3).

### Recalibrated perception maps to Δ motor output

The flexible properties of the mapping mechanism indicate that its adjustments to Δ motor output (Fig. 7, *x*_*m*_, dark blue) are computed from the *recalibrated* perception of speed difference $\tilde{p}$. During the Ramp Down task (Figs. 3–4), mapping-related adjustment *complements* recalibration-related adjustment to produce the overall Δ motor output that *tracks* the perturbation. This indicates that mapping-related adjustment is computed as *total* belt speed difference perturbation *minus* recalibration-related adjustment, which corresponds to the *perceived* perturbation $x_{m}\text{=}\tilde{p}=p-x_{r}$. As further evidence, we observe that the sharp “corner” in the Δ motor output data of the Ramp Down occurs when the *perceived,* not actual, perturbation changes sign (Fig. 4, mapping stops complementing recalibration and remains zero when the right leg *feels* slower than the left even though it is *actually* faster). Features of the mapping mechanism are further discussed in SI Appendix, Supplementary Discussion 4.

We propose the following series of computations (Fig. 7). First, the recalibration is used to compute the perceived perturbation (green box). Second, the perceived perturbation is relayed to the mapping mechanism and used to compute the mapping-related motor adjustment (dark blue box). Third, recalibration-related and mapping-related motor adjustments are summed to produce the Δ motor output (blue box). Interconnectivity between sensory and motor cortical areas, and the cerebellum (Judd et al., 2021; Kandel et al., 2013; Sultan et al., 2012), is consistent with the architecture proposed here. In contrast to the fixed adjustments typically attributed to predictive models in the cerebellum, flexible control of the walking pattern has traditionally been associated with cortical motor areas (Drew and Marigold, 2015; Reisman et al., 2010). Cortical involvement appears to go beyond voluntary control (Delval et al., 2020; Petersen et al., 2012) and may specifically contribute to split-belt adaptation (Hinton et al., 2019). More generally, cortical motor areas receive extensive sensory input from primary and association areas of the parietal lobe (Kandel et al., 2013), and use it to guide movement in a variety of sensorimotor mappings tasks (Dassonville et al., 2001; Georgopoulos, 2000). As such, we suggest cortical motor areas are the ideal substrate to receive perceptual signals (such as the perceived perturbation; Fig. 7, $\tilde{p}$, green) and map it to motor adjustments ($x_{m}\text{=}\tilde{p}$, “mapping” dark blue box).

Finally, our framework suggests that mapping-related motor adjustments are combined with forward model recalibration downstream (*u* = *x*_*m*_ + *x*_*r*_, “Δ motor output” blue box). Signals may be combined at the cortical level, as suggested by cortical correlates of adaptation in monkeys that resemble the combined operation of recalibration-like and mapping-like mechanisms (Chase et al., 2012; Sun et al., 2022) (SI Appendix, Supplementary Discussion 5). Alternatively, signals may be combined subcortically, as the cerebellum modulates movement through both cortical and subcortical projections (Kandel et al., 2013), and cerebral damage does not block walking adaptation (Choi et al., 2009; Reisman et al., 2007).

### Mapping operates as memory-based in some people, structure-based in others

Results from Experiment 2 highlight individual differences in the learning mechanisms underlying generalization to unexperienced belt speed differences (usage of the term “generalization” is discussed in SI Appendix, Supplementary Discussion 6). The generalization to novel perturbation sizes observed here is in line with previous suggestions of “meta-learning” in the savings of walking adaptation (i.e., faster relearning when exposed to a different perturbation) (Kristan A Leech et al., 2018; Malone et al., 2011). Generalization to *larger* perturbations after reaching adaptation was shown to be incomplete (Abeele and Bock, 2001; Lazar and Van Laer, 1968), and it was unclear whether the Δ motor output had been *extrapolated* beyond what had been experienced, or rather was just the same as in adaptation. Surprisingly, we found 40%–60% divide in our participants regarding the capacity to extrapolate Δ motor output to account for larger perturbations.

We suggest that structural learning may underly the ability to extrapolate the Δ motor output and walk symmetrically for belt speed differences larger than adaptation, as seen in 8 of 20 participants. Indeed, generalization in reaching adaptation may rely on a process of structural learning (Bond and Taylor, 2017; Braun et al., 2009) (note however that this was tied to explicit aiming, (Bond and Taylor, 2017)). Our participants may have learned the scaling relationship between belt speed perturbation and Δ motor output needed to walk symmetrically and use this scaling to produce new Δ motor outputs matching larger perturbations. In contrast, memory-based theories of mapping (Dassonville et al., 2001; Poggio and Bizzi, 2004; Van Vugt and Ostry, 2018; Wolpert et al., 2001) may explain why 12 participants walked asymmetrically for perturbations larger than adaptation, despite generalizing to smaller perturbations. Here, Δ motor outputs may be stored in memory during adaptation in association with the amount of perturbation they correct, and then retrieved during the ramp tasks. People transition through a range of Δ motor outputs in adaptation (see gradual transition from baseline to adapted output in Fig. 1B), so storage of intermediate outputs may reflect generalization to smaller perturbations (while larger Δ motor outputs are not experienced). SI Appendix, Supplementary Discussion 7, discusses potential *interpolation* computations.

In sum, consistent with findings that reaching adaptation strategies can be memory or structure-based (McDougle and Taylor, 2019), we show that walking adaptation can involve either memory-based or structure-based mappings.

### Conclusions and future directions

We here characterized two distinct learning mechanisms involved in walking adaptation, both of which are not under explicit control: recalibration - which leads to motor and perceptual aftereffects, and mapping – which only changes movement, does not contribute to aftereffects, and shows meta-learning to different perturbation sizes. Future work should explore whether the mapping mechanism described here could be of clinical significance: given the combined flexibility and automaticity, mapping has the potential to ameliorate known problems such as transitioning between walking environments (Sombric et al., 2017) and dual-tasking (Clark, 2015). Furthermore, our findings that different people adapt using different learning mechanisms (i.e., memory or structure-based mapping) highlights the importance of assessing individual characteristics of adaptation for both understanding of the neural mechanisms as well as translation to rehabilitation. Finally, as detailed in SI Appendix, Supplementary Discussions 8–10, the framework proposed here has important implications for the development of computational models of adaptation, and may help reconcile different findings related to savings, energetics, and sources of errors.

## Materials and Methods

### Participants

We recruited one-hundred adults (66 females, 23.6 ± 3.9 years old - mean ± SD) for this study, and we reanalyzed data from ten additional adults collected in a previous study (Kristan A. Leech et al., 2018) (9 female, 21.3 ± 2.9 years old). The protocol was approved by the Johns Hopkins Institutional Review Board and participants provided written informed consent. Participants had no known neurological or musculoskeletal disorders, were naïve to split-belt walking, and participated in only one of the nine experiments.

### Data collection

Participants walked on a split-belt treadmill (Woodway, Waukesha, WI, USA) and belt speeds were controlled using a custom Vizard program (WorldViz). Kinematic data were collected using infrared-emitting markers (Optotrak, Northern Digital, Waterloo, ON, Canada) at 100Hz (SI Appendix, Supplementary Methods).

### Ramp tasks

In the Ramp Up, Ramp Down, and Ramp Up and Down tasks of Experiments 1 and 2, we changed the speed of the right belt by 0.05m/s every 3 strides during right leg swing. The Ramp Up consisted of 7 increasing speeds from 0.35m/s to 0.65m/s. The Ramp Down consisted of 21 decreasing speeds from 1.5m/s to 0.5m/s. The Ramp Up and Down consisted of 41 total speeds: 11 increasing speeds from 1.5m/s to 2m/s, followed by 30 decreasing speeds from 1.95m/s to 0.5m/s. The left belt speed was constant at 0.5 m/s for all ramp tasks. The treadmill was not stopped before the ramp tasks (people transitioned directly from the preceding walking blocks into the ramp) but was briefly stopped after each ramp task. We occluded vision and sound of the belt speeds using a cloth drape and headphones playing white noise.

In Experiment 1, a keyboard was placed on the treadmill handrail and participants were asked to press a button the first time they perceived the right belt to be (1) as fast as the left, and (2) faster/slower than the left (for the Ramp Up and Ramp Down, respectively).

### Questionnaire

At the end of Experiment 2, participants answered this question on a computer: *Did you deliberately change how you walked to account for how fast the belts were moving? If so, describe how. Note: deliberately means that you thought about and decided to move that way. The question refers to the entire central ~20min walking block*. To ensure participants remembered what phase they were asked about, before adaptation we told participants that the following block will be called “central ~20min walking block”.

### Motor measures

We defined a stride as the period between two consecutive left heel strikes (*LHS*_1_ to *LHS*_2_), and computed the motor measures for each stride as in previous work (Finley et al., 2015; Sombric et al., 2017):

(9) $$\;\text{step}\;\text{length}\;\text{asymmetry}\;=\frac{1}{\;\text{stride}\;\text{length}\;}*\Delta \;\text{step}\;\text{length}\;$$

(10) $$\;\text{perturbation}\;=\frac{1}{\text{stride}\;\text{length}}*\bar{\;\text{step}\;\text{time}}*\Delta \;\text{step}\;\text{velocity}$$

(11) $$\Delta \text{motor}\;\text{output}=\frac{1}{\text{stride}\;\text{length}}*(\Delta \text{step}\;\text{position}-\bar{\text{step}\;\text{velocity}}*\Delta \text{step}\;\text{time})$$

The terms in the equations above are defined as follows:

(12) Δsteplength=Rsteplength−Lsteplength

(13) stridelength=Rsteplength+Lsteplength

Where right (*R*) and left (*L*) step lengths are the anterior-posterior distance between the ankle markers of the two legs at right heel strike (*RHS*) and left heel strike (*LHS*_2_) respectively.

(14) $$\Delta \;\text{step}\;\text{position}=\left(R\;\text{step}\;\text{position}{\;}_{RHS}-L\;\text{step}\;\text{positio}n_{LHS{\;}_{1}}\right)\;-\left(\;L\;\text{step}\;\text{positio}n_{LHS_{2}}-R\;\text{step}\;\text{position}{\;}_{RHS}\right)$$

Where step position is the anterior-posterior position of the ankle marker, relative to average of the two hip markers, at heel strike of the same leg.

(15) Δsteptime=Rsteptime−Lsteptime

(16) $$\bar{\text{step}\;\text{time}}=\frac{R\;\text{step}\;\text{time}+L\;\text{step}\;\text{time}}{2}$$

Where left and right step times are the times from *LHS*_1_ to *RHS*, and from *RHS* to *LHS*_2_ respectively.

(17) Δstepvelocity=Rstepvelocity−Lstepvelocity

(18) $$\bar{\text{step}\;\text{velocity}}=\frac{R\;\text{step}\;\text{velocity}+L\;\text{step}\;\text{velocity}}{2}$$

Where step velocity is the average anterior-posterior velocity of the ankle marker relative to average of the two hip markers, over the duration of the step (left: *LHS*_1_ to *RHS*, right: *RHS* to *LHS*_2_).

In Experiment 2, we also computed the measure of “strides to plateau” using individual step length asymmetry data from the adaptation phase only. We first smoothed the data with a 5-point moving average filter. We then calculated the number of strides till five consecutive strides fell within the mean ± 1SD of the last 30 strides (Malone and Bastian, 2010; Rossi et al., 2019).

### Clustering analysis

We tested to see if there were separate clusters of participants in our dataset. For Experiment 1, the measure used for clustering was the number of strides in the Ramp Down with step length asymmetry above the baseline CI. For Experiment 2, it was the number of strides in the first portion of the Ramp Up and Down (first 60 strides) with step length asymmetry below the baseline CI. For both experiments, “baseline CI” was computed separately for each participant as the 95% CI of the mean of step length asymmetry data in the second baseline tied-belt block (after the Ramp Up; see Statistical Analysis).

We used the MATLAB “dbscan” density-based clustering algorithm (Ester et al., 1996) and automated the choice of parameters adapting previously published procedures (Naik Gaonkar and Sawant, 2013; Rahmah and Sitanggang, 2016) (SI Appendix, Supplementary Methods and pseudocode). The resulting algorithm does not require any user input; it automatically assigns participants to clusters (whose number is not predefined), or labels them as outliers, based solely on each participant’s measure of interest.

### Data fitting

In Experiment 1, we fitted the recalibration only (Eq. 1), mapping only (Eq. 2), recalibration + mapping (Eq. 3) models to individual participants’ Δ motor output data in the Ramp Down. A reasonable upper limit for the parameter *r* would be the magnitude of the perturbation in adaptation, but this motor measure differs across participants, which would lead to inconsistencies in the data fitting analysis. We therefore reformulated the recalibration and recalibration + mapping models to use a normalized parameter *r*_norm_:

(19) $$r=r_{\text{norm }}*p_{\text{plateau }}$$

(20) $$\text{ Forward}\;\text{Model}\;\text{Recalibration}:\;u(p)=r_{\text{norm }}*p_{\text{plateau }}$$

(21) $$\text{Recalibration+mapping}:\;u(p)=\{\begin{array}{ll} p, & \text{ for }p\geq r_{\text{norm}}*p_{\text{plateau}}\\ r_{\text{norm}}*p_{\text{plateau}}, & \text{otherwise } \end{array}$$

Where *p*_plateau_ is mean perturbation over the last 30 strides of adaptation, computed separately for each participant, *p* is the perturbation in the Ramp Down, and *u* is the modelled Δ motor output. We fitted the normalized parameter *r*_norm_ using initial parameter value = 0.5 and bounds = [0, 1] for all participants. We then used Eq. 19 to compute the unnormalized value *r*.

We additionally fitted the dual state model defined as in Smith et al. (Smith et al., 2006):

(22) $$\Delta \text{motor}\;\text{output}:\;u(i)=u_{f}(i)+u_{s}(i)$$

(23) $$\text{fast state}:\;u_{f}\text{(}i\text{+1)=}A_{f}\text{*}u_{f}\text{(}i\text{)+}B_{f}\text{*(}p\text{(}i\text{)-}u\text{(}i\text{))}$$

(24) $$\text{slow state}:\;u_{s}\text{(}i\text{+1)=}A_{s}\text{ * }u_{s}\text{(i)+}B_{s}\text{*(}p\text{(}i\text{)-}u\text{(}i\text{))}$$

Where *p* is the perturbation, *i* is the stride in the task (0 to 62), *u* is the fitted Δ motor output, *u*_*f*_ and *u*_*s*_ are the contribution of the fast and slow states to changing the Δ motor output, and *A*_*f*_, *A*_*s*_, *B*_*f*_ , *B*_*s*_ are free parameters representing forgetting and learning rates, with 0<*A*_*f*_ <*A*_*s*_ <1 and 0<*B*_*f*_>*B*_*s*_<1. We set the initial values as *A*_*f*_=0.92, *A*_*s*_=0.99, *B*_*f*_=0.1, and *B*_*s*_=0.01 (as in (Roemmich et al., 2016)).

We used the *fmincon* MATLAB function to find the parameters of each model that minimize the residual sum of squares between data and model fits. We tightened constraint and optimality tolerances to 10^−20 (as in (Roemmich et al., 2016)). For the dual state model, we fitted the model to both adaptation and Ramp Down data (this was necessary as the initial value of the fast and slow states in the Ramp Down was otherwise unknown), but computed the residual sum of squares solely on the Ramp Down (ensuring fair model comparison).

### Statistical analysis

Statistical analyses were performed in MATLAB with significance level α = 0.05 corrected for multiple comparisons using the False Discovery Rate procedure (FDR) (Benjamini and Yekutieli, 2005) (corrected significance level $\alpha_{\text{corr }}=\frac{\alpha R}{m}$, with m = number of total tests for that comparison group, reported below, and R = number of significant tests). We used a bootstrap procedure (10,000 samples drawn with replacement) (DiCiccio and Efron, 1996; Efron and Tibshirani, 1994) to generate confidence intervals (CI) for the following measures:

- Group mean step length asymmetry for each speed in the Ramp Up (m=7) and Ramp Down (m=21) tasks of Experiment 1 (pre-averaged within-participant over the 3 strides taken at that speed).
- Group mean value for the BIC difference between the recalibration + mapping model fit, and each of the other model fits (dual state, recalibration only, mapping only) of Experiment 1 (m=3).
- Group mean value for the difference between *compensation*_motor total_ or *compensation*_motor recalibration_, minus each *compensation*_perceptual_ (Experiment 1, m=4).
- Difference of the means between Structure and Memory subgroups (Experiment 2) for the measures of strides to plateau (m=1), and number of asymmetric strides in Ramp Up and Down (teal portion; m=1)
- Individual means for baseline step length asymmetry (2-min block) for each participant in Experiment 1 and 2 (all m=1).

We report FDR-corrected CIs and consider statistical tests significant if the CI does not overlap zero (Cumming and Finch, 2005; DiCiccio and Efron, 1996; Efron and Tibshirani, 1994).

## Supplementary Material

Supplement 1

## Data Availability

Note to reviewers: the dataset cited below is currently “private for peer review”, and can be accessed by the alternative “reviewer URL” provided by the Dryad database: https://datadryad.org/stash/share/Qa0pD7rMFT7kVgDeP-8PMYKBaCNuy4yx1VGIQlpVgvM All data and code used for the study have been deposited in: Rossi, Cristina (2023), Dataset for automatic learning mechanisms for flexible human locomotion, Dryad, Dataset, https://doi.org/10.5061/dryad.18931zd27.

## Abbreviations

CI: confidence interval
CI_LB_: lower bound of CI
CI_UB_: upper bound of CI
PSE: point of subjective equality
norm: normalized
SD: standard deviation
SE: standard error
