# Automatic learning mechanisms for flexible human locomotion

**DOI:** 10.1101/2023.09.25.559267

**Published:** 2024-07-24

**Authors:** Cristina Rossi, Kristan A. Leech, Ryan T. Roemmich, Amy J. Bastian

## Abstract

Movement flexibility and automaticity are necessary to successfully navigate different environments. When encountering difficult terrains such as a muddy trail, we can change how we step almost immediately so that we can continue walking. This flexibility comes at a cost since we initially must pay deliberate attention to how we are moving. Gradually, after a few minutes on the trail, stepping becomes automatic so that we do not need to think about our movements. Canonical theory indicates that different adaptive motor learning mechanisms confer these essential properties to movement: explicit control confers rapid flexibility, while forward model recalibration confers automaticity. Here we uncover a distinct mechanism of treadmill walking adaptation – an automatic stimulus-response mapping – that confers both properties to movement. The mechanism is flexible as it learns stepping patterns that can be rapidly changed to suit a range of treadmill configurations. It is also automatic as it can operate without deliberate control or explicit awareness by the participants. Our findings reveal a tandem architecture of forward model recalibration and automatic stimulus-response mapping mechanisms for walking, reconciling different findings of motor adaptation and perceptual realignment.

## Abbreviations

CI, confidence interval; $CI_{LB}$, lower bound of CI; $CI_{UB}$, upper bound of CI; PSE, point of subjective equality; norm, normalized; SD, standard deviation; SE, standard error

## Main Text

## Introduction

Flexibility and automaticity are essential features of human movement, so much so that we rarely think about the details of how we move. While walking, we do not think about the bend of the ankle or how quickly to swing the leg forward to step. Yet, we easily adjust walking to accommodate many situations – a muddy trail, a grassy slope, or a snowy path. These abilities are often taken for granted until something goes awry – a sprained ankle quickly brings the details of movement execution to awareness, and deliberate control is often used to avoid pain or further injury. It is only then that we truly appreciate how much the sensorimotor system is doing without our conscious awareness.

We currently do not understand how different motor learning mechanisms confer both flexibility and automaticity to human movement. One well-studied process of motor learning is sensorimotor adaptation, which occurs in response to errors between the expected and actual sensory consequences of our movements. Accordingly, this motor learning mechanism helps adjust motor commands to correct for perturbations to our movements caused by altered environmental demands (Bastian, 2008; Huberdeau et al., 2015).

Adaptation is traditionally thought to rely on the cerebellum-dependent recalibration of a forward model that associates motor commands with expected sensory consequences (Ito, 1989; Wolpert et al., 2001). For example, to walk on an icy sidewalk, we may need to recalibrate our prediction of how firmly our feet will grip the ground. The process of forward model recalibration is automatic and implicit, as it has been shown to operate without intention or awareness (Huberdeau et al., 2015; Taylor et al., 2014). However, forward model recalibration does not confer rapid flexibility because it cannot make immediate changes in movement – it can only adjust movement gradually trial-by-trial or step-by-step, a process that can take several minutes (Bastian, 2008; Huberdeau et al., 2015). The newly acquired sensorimotor recalibration must be unlearned over time to restore a normal movement when the environment returns to its original state after adaptation (Bastian, 2008; Martin et al., 1996a). For this reason, forward model recalibration leads to lasting movement errors (called “aftereffects”).

Sensorimotor adaptation can rely on more than forward model recalibration. We know that adaptation of goal-directed reaching movements can involve rapid flexible learning mechanisms sometimes called "stimulus-response mapping mechanisms". Different types of stimulus-response mapping mechanisms have been characterized in previous studies: First, explicit strategies – where people deliberately change where they are aiming to reach (Taylor et al., 2014; Taylor and Ivry, 2011). Second, memory-based caching – where people learn motor responses in association with the respective environmental sensory stimuli, and cache them in memory for future retrieval (like a lookup-table) (Huberdeau et al., 2019; McDougle and Taylor, 2019). Third, structural learning – where people learn general relationships between environmental sensory stimuli and motor responses and use them to produce novel responses (like when we learn to map the movement of a computer mouse to that of the cursor) (Bond and Taylor, 2017; Braun et al., 2009). Stimulus-response mapping mechanisms differ from forward model recalibration in that they confer rapid flexibility – novel responses can be promptly abandoned or changed for different environmental stimuli – and therefore do not lead to aftereffects (Huberdeau et al., 2015; Taylor et al., 2014; Taylor and Ivry, 2011).

It is unclear if stimulus-response mapping mechanisms are involved in adapting movement types that are continuous like walking versus discrete movements like reaching. The goal of this study was to understand whether walking adaptation involves any stimulus-response mapping mechanism and, if so, how it operates and how it can be dissected from forward model recalibration. We focused on adaptation of walking movements on a split-belt treadmill: it is well established that when people walk with one foot faster than the other, they adapt the timing and location of their step to restore symmetry (Box 1).

In Experiment 1A we tested the presence of stimulus-response mapping during gait adaptation by evaluating whether people develop the ability to modify their walking pattern immediately for different split-belt magnitudes. Preliminary evidence suggests that people may be able to switch between at least two different walking patterns more rapidly than we would expect from forward model recalibration alone (Box 2) (Leech et al., 2018a). Based on this evidence, we hypothesized that walking adaptation involves stimulus-response mapping in addition to forward model recalibration.

As our findings corroborated this hypothesis, in Experiment 1B we aimed to develop a measure to dissect individual contributions of the two mechanisms to adaptation. We based this on the well-known phenomenon of "perceptual realignment", where perception of the belt speed difference diminishes over time during adaptation (Box 2) (Jensen et al., 1998; Leech et al.,

2018a; Rossi et al., 2019; Vazquez et al., 2015). Previous studies show that perceptual realignment is only partial – the belt speeds feel similar but not completely equal at the end of adaptation, despite complete motor adaptation (Leech et al., 2018a; Vazquez et al., 2015). Studies also suggest that perceptual realignment may stem from forward model recalibration processes ('t Hart and Henriques, 2016; Izawa et al., 2012; Rossi et al., 2021a; Synofzik et al., 2008). Based on these studies, we hypothesized that the extent of perceptual realignment corresponds to the contribution of forward model recalibration to motor adaptation, with the remainder attributed to stimulus-response mapping.

Finally, in Experiment 2 we began to explore characteristics of stimulus-response mapping. We asked whether the stimulus-response mapping mechanism is automatic, or instead under deliberate or explicit control. Stimulus-response mapping mechanisms for reaching require explicit control (Bond and Taylor, 2017; Huberdeau et al., 2019; Taylor and Ivry, 2011), but explicit control is poorly suited for automatic, continuous movements like walking (Clark, 2015; Paul et al., 2005; Uiga et al., 2020), and can even lead to falls (Wong et al., 2008). Unlike reaching, adaptation of walking is unaffected by explicit goals or instructions given to the participants on where to aim their feet (Long et al., 2016; Roemmich et al., 2016). Hence, we hypothesized that participants would not be able to describe how they changed walking, in contrast to what has been previously reported in reaching. We also asked if participants could use the mapping mechanism to produce novel motor outputs akin to what has been interpreted as structural learning in reaching (Bond and Taylor, 2017; Braun et al., 2009).

## Results

### Experiment 1

*Motor paradigm and hypotheses*

We asked whether walking adaptation involves both forward model recalibration and stimulus-response mapping mechanisms learned in tandem ("recalibration + mapping hypothesis"). We contrasted this with the alternative hypothesis that walking adaptation may involve only forward model recalibration mechanisms ("recalibration only hypothesis").

To test this, we devised the "Ramp Down" paradigm depicted in Figure 1A. After adaptation, we gradually decreased the speed of the right belt every 3 strides (see Methods). This enabled us to obtain reliable measurements of aftereffects across 21 predetermined speed configurations, spanning from the full split-belt perturbation to tied-belts. We measure aftereffects as the magnitude of step length asymmetry.

As depicted in Figure 1B, the recalibration only hypothesis predicts that aftereffects will be present for all speed configurations in the Ramp Down. This is because the Δ motor output (blue) can only change gradually, through unlearning or forgetting of the forward model recalibration (SI Appendix, Fig. S1). This change is too slow to track the perturbation (red), which is ramped down to zero rapidly over ~80 seconds. Therefore, step length asymmetry (purple) becomes positive immediately in the Ramp Down.

In contrast, as depicted in Figure 1C, the recalibration + mapping hypothesis predicts that aftereffects will be present for some, but not all speed configurations in the Ramp Down. Specifically, there would be no aftereffects (~zero step length asymmetry) in the first portion of the Ramp Down, because the Δ motor output (blue) changes rapidly and matches the perturbation (red). This reflects the flexibility of the stimulus-response mapping (SI Appendix, Fig. S1). Aftereffects would emerge in the second portion of the task (positive step length asymmetry), reflecting forward model recalibration.

We include forgetting effects for the recalibration only hypothesis to account for previous findings from Leech et al. (Leech et al., 2018a) (Box 2). The inset of Figure 1 (right column) illustrates how both hypotheses may account for near-zero step length asymmetry at the end of the speed match task. The Δ motor output decreases to match the perturbation either because the mapping mechanism flexibly scales down (recalibration + mapping hypothesis) or, alternatively, because of forgetting/unlearning (recalibration only hypothesis).

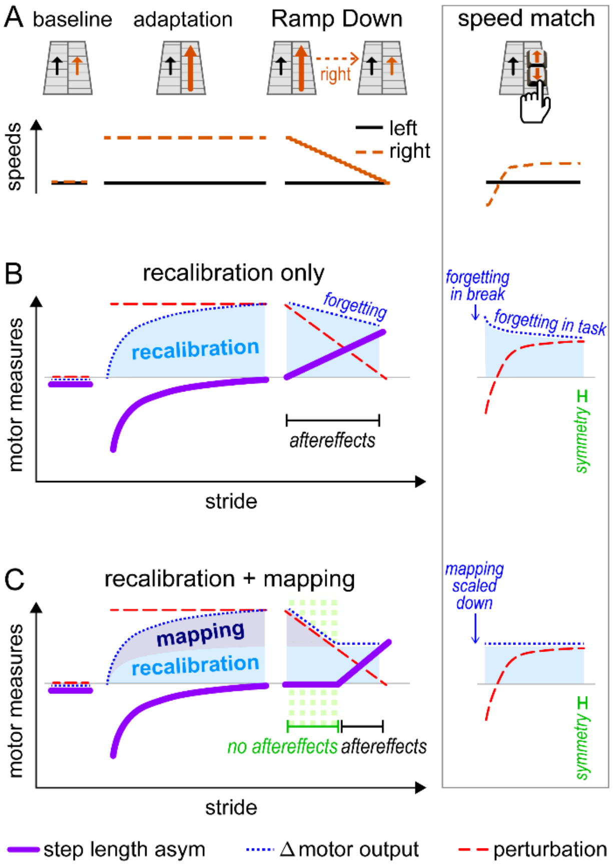

**Figure 1. Experiment 1, Hypotheses and Predictions. (A)** Conceptual schematic of our paradigm with the Ramp Down task: after adaptation, the right belt speed is gradually ramped down to match the left. **(B-C)** Predictions for the Ramp Down motor measures made by two competing hypotheses. **(B) Recalibration only:** recalibration can only change movement gradually. The Δ motor output (dotted blue line) changes slowly and does not track the rapidly decreasing perturbation (dashed red line), so that step length asymmetry aftereffects emerge immediately (solid purple line, magnitude is positive). **(C) Recalibration + mapping:** mapping can change movement immediately. In the first part of the task (highlighted in green), the mapping contribution to Δ motor output (dark blue shade) is scaled down immediately as the perturbation decreases. Hence, the Δ motor output (dotted blue line) changes rapidly and tracks the perturbation (dashed red line), so that there are no step length asymmetry aftereffects (solid purple line, magnitude is ~zero). In the second part of the task, the mapping contribution to Δ motor output is zero, and the recalibration contribution to Δ motor output (light blue shade) does not change significantly. Hence, the Δ motor output (dotted blue line) does not track the perturbation (red dashed line), and step length asymmetry aftereffects emerge (solid purple line, magnitude is positive). **Right column inset:** conceptual explanation of how both hypotheses may account for the speed match results from Leech at al. (Leech et al., 2018a). In the first post-adaptation speed match task, participants increase the speed of the right belt from zero to a value that is smaller than adaptation but larger than the left belt (top panel). The perturbation increases until a value that is positive but smaller than adaptation (dashed red line, middle and bottom panels). Leech et al. observed symmetric step lengths at the end of the task, indicating that the Δ motor output (dotted blue line) is smaller than it was in adaptation and matches the perturbation. The decrease in Δ motor output can be explained by the recalibration only hypothesis as forgetting/unlearning (middle panel), or by the recalibration + mapping hypothesis as flexible scaling of the mapping contribution (bottom panel).

*Motor results – step length asymmetry*

Figure 2A shows the paradigm used for Experiment 1, and the time course of step length asymmetry (group mean ± SE). The Ramp Down task corresponds to the manipulation illustrated in Figure 1 and is the focus of our analysis for Experiment 1. A similar ramp task was performed prior to adaptation as a control to see how participants responded to a gradually ramped perturbation at baseline (see Methods, "Ramp tasks"). Participants performed perceptual tests during the ramp tasks, which will be discussed in a later section (see "Perceptual results"). Patterns of step length asymmetry during other portions of the paradigm were consistent with a large body of previous work (e.g., Leech et al., 2018a, 2018b; Reisman et al., 2005; Roemmich et al., 2016; Rossi et al., 2019; Vazquez et al., 2015) and will not be discussed further.

Step length asymmetry during the baseline ramp and post-adaptation Ramp Down tasks are displayed in Figure 2B. For each speed configuration in the Ramp Down task, we statistically compared step length asymmetry to zero. We evaluated the emergence of aftereffects by examining when step length asymmetry became significantly positive. We found that step length asymmetry was *not* statistically different from zero during the first half of the Ramp Down task, for right belt speeds ranging from 1m/s to 0.5m/s faster than the left speed (these speed configurations are highlighted in green in Fig. 2B right; all $CI_{LB}$ [confidence intervals lower bounds] ≤ -0.001 and $CI_{UB}$ [upper bounds] ≥ 0.009, SI Appendix, Fig. S2 and Table S1). This result indicates that aftereffects do not emerge immediately in the Ramp Down task, a finding at odds with the recalibration only prediction (Fig. 2B 'predictions' inset, top). Step length asymmetry was instead significantly positive for the second half of the task, for right belt speeds ranging from 0.45m/s to 0m/s faster than the left speed (all $CI_{LB} > 0.01$). These results indicate that aftereffects emerge at a mid-point during the Ramp Down task and align with behavioral predictions from the recalibration + mapping hypothesis (Fig. 2B 'predictions' inset, bottom).

As a control, we repeated the same analysis for the baseline ramp task (Fig. 2B, left). We found that there was only one speed configuration for which step length asymmetry was not statistically different from zero (right speed 0.05m/s slower than left; SL asym. = 0.006 [-0.017, 0.028], mean [CI]). For all other configurations, step length asymmetry was significantly positive ($CI_{LB} \geq 0.048$ for right speed 0.1 to 0.35m/s slower than left) or negative ($CI_{UB} \leq -0.019$ for right speed 0 to 0.35m/s faster than left). This is in stark contrast to the wide range of speed configurations with near-zero step length asymmetry observed in the Ramp Down period. Hence, the ability to walk symmetrically at different speed configurations is not innate but dependent on the adaptation process, corroborating the hypothesis that the "no aftereffects" region of the Ramp Down reflects the operation of a mapping mechanism.

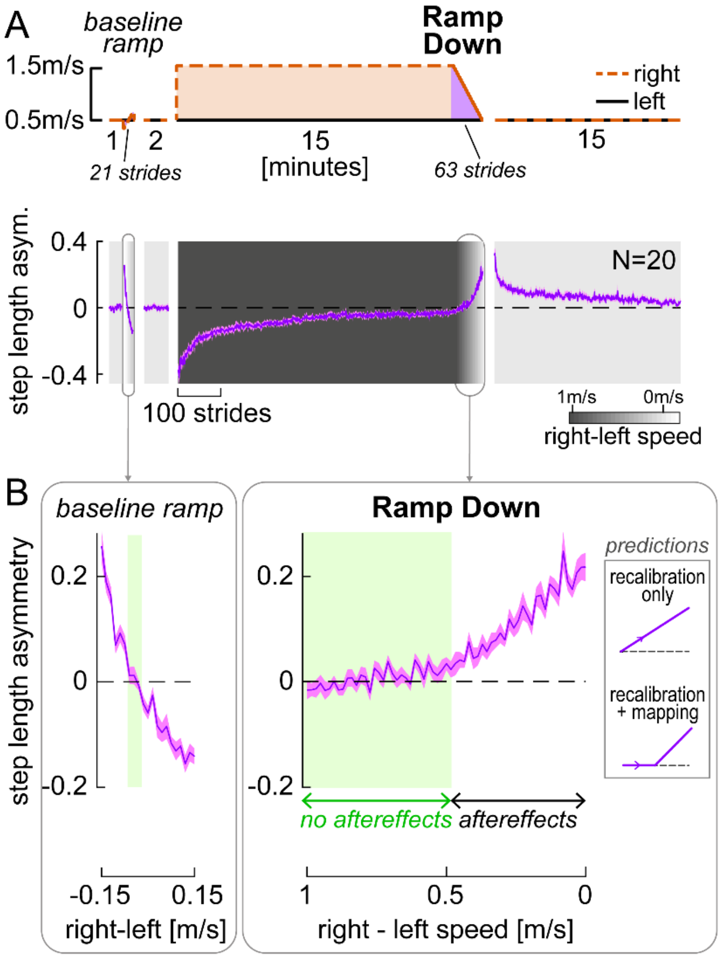

**Figure 2. Experiment 1, Step length asymmetry. (A)** Top: Experimental protocol. The Ramp Down task (purple) is used to test the predictions illustrated in Fig. 1. Bottom: Step length asymmetry time course. Background shading darkness increases with belt speed difference (color bar). Phases (except Ramp tasks) are truncated to the participant with fewest strides. **(B)** Zoomed-in baseline ramp and post-adaptation Ramp Down tasks. Speed differences for which step length asymmetry is not significantly different from zero are indicated by the green shade. Inset depicts predictions made by the competing hypotheses as in Fig. 1. All curves show group mean ± SE.

*Motor results – perturbation and Δ motor output*

To further test the competing hypotheses, we examined the perturbation and Δ motor output during the Ramp Down (Figure 3A). As the belt speed difference decreased, so did the perturbation component (red). In the first half of the task, the Δ motor output (blue) appeared to match the perturbation – consistent with the lack of step length asymmetry aftereffects (green shaded portion, matching Fig. 2B). Additionally, in the second half of the task, the Δ motor output appeared larger than the perturbation – consistent with the positive step length asymmetry aftereffects observed in Fig. 2B.

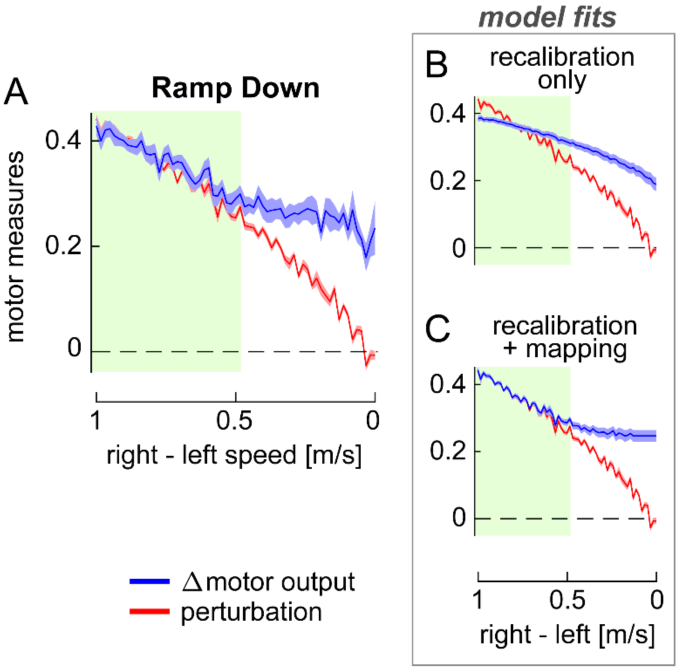

**Figure 3. Experiment 1, Perturbation and Δ motor output. (A)** Perturbation (red) and Δ motor output (blue) data for the Ramp Down task. **(B-C)** Perturbation data (red) and model fit for the Δ motor output (blue) for recalibration only and recalibration + mapping models. All curves show group mean ± SE. Green shade corresponds to speeds with symmetric step lengths as in Fig. 2.

We formally contrasted predictions made by the competing hypotheses by developing mathematical models of the Δ motor output as a function of perturbation (Fig. 3B-C). In the simplest framework, the Δ motor output behavior for the recalibration and mapping mechanisms can be formalized as follows:

Forward Model Recalibration: $$u(p) = r \quad (1)$$

Stimulus-Response Mapping: $$u(p) = p \quad (2)$$

Recalibration + Mapping: $$u(p) = \begin{cases} p & , \quad \text{for } p \geq r \\ r & , \quad \text{otherwise} \end{cases} \quad (3)$$

Where $u$ is the modelled Δ motor output, $p$ is the perturbation, and $r$ is a free parameter representing the portion of the Δ motor output related to recalibration. We used equation (3) as our model for the recalibration + mapping hypothesis. This describes the scenario where participants modulate the Δ motor output to match the perturbation ($u(p) = p$) for the first portion of the Ramp Down task. The Δ motor output remains constant during the second portion of the task ($u(p) = r$) because participants are unable to reduce the Δ motor output to values smaller than “$r$” (the amount achieved by recalibration). Parameters were estimated by fitting the model to individual participants’ Δ motor output data from the Ramp Down (see Methods).

The fitted Δ motor output is displayed in Figure 3C (mean ± SE of fits across participants; individual fits are shown in SI Appendix, Fig. S3). As expected, the recalibration + mapping fit captured the matching-then-divergent behavior of Δ motor output in response to the changing perturbation.

We used the dual state model of motor adaptation from Smith et al. (Smith et al., 2006) as our model for the recalibration only hypothesis (see Methods). The model has four parameters and can account for potential forgetting or unlearning of the Δ motor output that may occur during the Ramp Down. It can also account for the possibility that adaptation involves two recalibration mechanisms – i.e., two distinct ‘fast’ and ‘slow’ mechanisms that both learn via a process of forward model recalibration, and both contribute to aftereffects, but that learn and forget at different rates. We chose this model because it is a well-established model that is widely used to capture the Δ motor output time-course for traditional motor adaptation paradigms (i.e., those consisting of adaptation and post-adaptation phases). Yet, performance of this model for a manipulation like the Ramp Down task performed here has not yet been tested. As such, if the recalibration + mapping model fit the Ramp Down data better than the dual state model, this would provide robust evidence for the presence of a stimulus-response mapping mechanism.

We show the Δ motor output fit by the recalibration only (dual state) model in Figure 3B (group mean ± SE; individual fits are shown in SI Appendix, Fig. S4). In contrast to the recalibration + mapping model, the dual state model was not able to capture the matching-then-divergent behavior of Δ motor output. The BIC statistic confirmed that the recalibration + mapping model fitted the data significantly better than the dual state (BIC difference = 8.422 [3.532, 13.573], mean [CI]).

In a supplementary analysis, we considered two additional prominent models for motor adaptation: optimal feedback control (Izawa and Shadmehr, 2011; Shadmehr and Krakauer, 2008; Todorov, 2004) and memory of errors (Herzfeld et al., 2014). Similar to the dual state, these models could not capture the matching-then-divergent behavior of Δ motor output and fitted the Ramp Down data significantly worse than the recalibration + mapping (see SI Appendix, "Experiment 1 additional models" and Fig. S5; memory of errors minus recalibration+mapping BIC difference = 11.610 [6.220, 17.558], optimal feedback control minus recalibration+mapping BIC difference = 19.272 [14.378, 24.399], mean [CI]). In sum, the modeling analysis of the Δ motor output further supports the recalibration + mapping hypothesis.

*Perceptual test and results*

The second goal of Experiment 1 was to evaluate the hypothesis that "perceptual realignment" (a phenomenon leading to altered perception following adaptation) results from the operation of the same forward model recalibration mechanism involved in adaptation of the Δ motor output (Rossi et al., 2021a).

Previous work shows that perception realigns following gait adaptation: after adapting to a perturbation where the right treadmill belt is faster than the left, return to tied belts results in perception of the opposite asymmetry (i.e., right speed feels slower than left) (Jensen et al., 1998;

Vazquez et al., 2015). We therefore expected that people would perceive the belt speeds as equal at some point in the Ramp Down and eventually perceive the opposite asymmetry on tied belts. We measured this by asking participants to press a keyboard button at two separate occasions during the Ramp Down task: (1) when the belts first felt equal, and (2) when they no longer felt equal (see Methods).

Figure 4A depicts the button presses for the Ramp Down perceptual test (top panel, group mean) overlayed onto the Ramp Down motor data (recalibration + mapping fit). Note that the task captures a *range* of belt speed configurations that participants perceive as "equal speeds", with button presses (1) and (2) corresponding to the upper and lower bounds of this range. This is expected because perception is known to be noisy and may not be sensitive enough to discriminate between belt speed configurations that are too similar.

We quantified perceptual realignment using the established measure of *point of subjective equality* (PSE), defined as the belt speed difference perceived as "equal speeds". A PSE of zero would indicate no perceptual realignment (accurate perception) and a PSE of 1m/s (i.e., a magnitude equivalent to the difference between the belt speeds during adaptation) would indicate complete perceptual realignment such that the belt speeds feel equal during the adaptation phase.

We measured belt speed difference at the time of each button press. We computed PSE as the range of belt speed difference values between these two measurements:

$$PSE_{\text{upper bound}} = [\, right\ belt\ speed - left\ belt\ speed\, ]_{\text{time of button press 1}} \qquad (4)$$

$$PSE_{\text{lower bound}} = [\, right\ belt\ speed - left\ belt\ speed\, ]_{\text{time of button press 2}} \qquad (5)$$

We found that $PSE_{\text{upper bound}}$ was 0.64±0.03m/s and $PSE_{\text{lower bound}}$ was 0.39±0.05m/s (Figure 4A, bottom panel, group mean ± SE). This was consistent with previous work (Leech et al., 2018a).This perceptual realignment was not present during baseline testing, as illustrated in SI Appendix, Fig. S6.

**Figure 4. Experiment 1, Perceptual Results. (A)** Top: perturbation data (red) and recalibration + mapping fit (blue); this is the same as Fig. 3C. Bottom: perceptual task button presses (green, group mean ± SE), as a function of belt speed difference. Right: measures of motor recalibration ("$r$") and total motor adaptation ("$u_{\text{plateau}}$"). **(B)** Perturbation compensation (normalized perceptual and motor measures of adaptation): $compensation_{\text{perceptual}}$ bounds (green), $compensation_{\text{motor total}}$ (dark blue), and $compensation_{\text{motor recalibration}}$ (light blue). **(C-D)** Individual participants' $compensation_{\text{motor recalibration}}$ versus $compensation_{\text{perceptual}}$ (first or second button press). Solid black: least squares line. Dashed gray: unity line.

We aimed to evaluate the hypothesis that perceptual realignment arises from the forward model mechanism of motor adaptation and is unaffected by the stimulus-response mapping mechanism. This hypothesis predicts that the extent of perceptual realignment should be: 1) approximately equal to the extent of motor adaptation achieved by recalibration, and 2) less than the total extent of motor adaptation (which also includes mapping). We quantified 1) motor adaptation by recalibration as the fitted parameter "*r*" from the recalibration + mapping model, and 2) total motor adaptation as the Δ motor output at adaptation plateau (Fig. 4A, right panel, see previous section "*Motor results – perturbation and Δ motor output*). We expressed these motor measures and perceptual realignment as "percent compensation for the perturbation" (i.e., normalized to the perturbation magnitude in the respective units) so that they could be compared:

$$compensation_{\text{perceptual}} = \frac{PSE}{1\,\text{m/s}} \quad (6)$$

$$compensation_{\text{motor recalibration}} = \frac{r}{p_{\text{plateau}}} \quad (7)$$

$$compensation_{\text{motor total}} = \frac{u_{\text{plateau}}}{p_{\text{plateau}}} \quad (8)$$

Where $r$ is the fitted parameter from the recalibration + mapping model, $p_{\text{plateau}}$ is the mean perturbation over the last 30 strides of adaptation, and $u_{\text{plateau}}$ is the mean Δ motor output over the last 30 strides of adaptation (for Eq. 6, note that 1m/s is the belt speed difference in adaptation).

We show group level compensation measures in Fig. 4B. We found that $compensation_{\text{motor recalibration}}$ (56±4%, group mean ± SE) fell within the $compensation_{\text{perceptual}}$ range (39±5% to 64±3%): it was significantly smaller than the upper bound (difference = -8 [-14, -2]%) and significantly larger than the lower bound (difference = 18 [8, 28]%, mean [CI]). This supports our first prediction that perceptual realignment is comparable to the extent of motor adaptation achieved by recalibration. Furthermore, $compensation_{\text{perceptual}}$ was significantly smaller than $compensation_{\text{motor total}}$ (95±2%, mean ± SE; difference = 31 [25, 38]% or 57 [48, 66]%, upper or lower perceptual bounds, mean [CI]). This supports our second prediction that perceptual realignment is less than the total extent of motor adaptation.

We show individual $compensation_{\text{motor recalibration}}$ and $compensation_{\text{perceptual}}$ measures for each participant in Figure 4C-D. We evaluated Pearson's correlation coefficients between these measures to test whether there is a direct relationship between perceptual realignment and the motor adaptation achieved by recalibration. We found that $compensation_{\text{motor recalibration}}$ was significantly correlated with the upper bound of $compensation_{\text{perceptual}}$ – the value computed using the "speeds feel equal" button press (r=0.64, p=0.002). This supports the hypothesized relationship between motor recalibration and changes to leg speed perception. Instead, $compensation_{\text{motor recalibration}}$ was not correlated with the lower bound of $compensation_{\text{perceptual}}$ (r=0.30, p=0.195) – this value is computed using the "right feels slower than left" button press, suggesting the true PSE may lay closer to the first button press. Together, our results support the hypothesis that perceptual realignment can be used as a proxy measure for the extent of motor adaptation achieved by forward model recalibration.

*Control experiments*

We performed six control experiments and reanalyzed previously-published data (Leech et al., 2018a) to replicate the findings of Experiment 1 across different paradigm conditions (SI Appendix, "Control experiments", Fig. S7-9, and Table S2-6). This provided additional support for the recalibration + mapping hypothesis. We first checked that the motor and perceptual behaviors observed in the Ramp Down could be replicated using a different method of assessment. A "speed match" task was given where participants use a joystick to increase the speed of the right belt (initially stationary) until they feel it matches the left belt (SI Appendix, Fig. S7B, "Ascend") (Leech et al., 2018a; Rossi et al., 2019; Statton et al., 2018; Vazquez et al., 2015). This approximates the second half of the Ramp Down (Fig. 4A, right leg feels equal or slower than left). Consistent with this, we observed step length asymmetry aftereffects early in the speed match task (initial SL asym. = 0.433 [0.271, 0.612], mean [CI]). Asymmetry decreased and was eventually near-zero when the belt speeds felt equal (final SL asym. = 0.002 [-0.040, 0.045]; SI Appendix, Fig. S8B).

We also used a "descend" speed match task where participants *decreased* the speed of the right belt (initially fast as in adaptation) until they felt it matched the left (SI Appendix, Fig. S7B). This approximated the first half of the Ramp Down. As expected, step length asymmetry was close to zero for entire task (initial: -0.017 [-0.069, 0.039], final: 0.043 [-0.0004, 0.090], mean [CI]; SI Appendix, Fig. S8B). The PSE for both speed match groups was comparable to that of Experiment 1 ($compensation_{\mathrm{perceptual}}$ difference between Ramp Down lower bound and ascend or descend Control = -3 [-16, 10]% or 4 [-12, 18]%, mean [CI]), and smaller than motor adaptation ($compensation_{\mathrm{motor\ total}} - compensation_{\mathrm{perceptual}}$ = 48 [39, 57]% or 56 [42, 70]% for ascend or descend, mean [CI]; SI Appendix, Fig. S8A).

We replicated our findings in additional speed match experiments that varied in adaptation duration (3, 15, or 30 minutes), perturbation magnitude (1m/s or 0.4m/s speed difference) and schedule (abrupt or gradual) (SI Appendix, "Control experiments" and Fig. S7). Regardless of the paradigm, the step length asymmetry and PSE were consistent with the recalibration + mapping hypothesis (SI Appendix, Fig. S8 and Table S2-3).

## Experiment 2

Experiment 1 demonstrated the presence of a stimulus-response mapping mechanism that can produce Δ motor outputs that match a range of perturbations *smaller* than the adaptation perturbation (in the Ramp Down task). In Experiment 2, we asked whether it can also produce Δ motor outputs that match perturbations *larger* than the adaptation perturbation.

This sheds light on whether the stimulus-response mapping of walking adaptation operates akin to memory-based or structure-based mechanisms observed in reaching adaptation. Failure to account for larger perturbations would suggest that it is memory-based. That is, the Δ motor outputs produced in adaptation may be cached in memory and later retrieved (Huberdeau et al., 2019; McDougle and Taylor, 2019; Poggio and Bizzi, 2004; Wolpert et al., 2001). These Δ motor outputs span a range of magnitudes that are smaller than the adaptation perturbation (see Box 1), so that they could match smaller but not larger perturbations.

Conversely, success in accounting for larger perturbations would suggest that the stimulus-response mapping may be structure-based. That is, it may learn the general relationship between perturbation and appropriate Δ motor output in adaptation and later use it to generate Δ motor outputs anew (Bond and Taylor, 2017; Braun et al., 2009; McDougle and Taylor, 2019; Wolpert et al., 2001). Hence, it would be able to generate Δ motor outputs to match either smaller or larger perturbations.

Thus, we tested a "Ramp Up & Down" condition where the speed of the right belt was both gradually increased and then decreased after adaptation (Fig. 5A). The memory-based hypothesis predicts that step length asymmetry will become negative for perturbations *larger* than adaptation, while the structure-based hypothesis predicts it will remain close to zero (Fig. 5B inset, "predictions"). The rest of the paradigm was analogous to that of Experiment 1 except there was no perceptual assessment. Figure 5B shows step length asymmetry throughout the paradigm, and Figure 5C shows a close-up of the Ramp Up & Down task performance (group mean ± SE). The magenta portion of the task corresponds to the Ramp Down of Experiment 1, but the step length asymmetry differs because of the exposure to larger speed differences in the teal portion (a supplementary analysis confirmed that this is consistent with the recalibration + mapping hypothesis; see SI Appendix, "Ramp Down comparison between Experiments 1 and 2", Fig. S10-11 and Table S7).

On average, participants' step length asymmetry patterns did not remain zero for speed differences larger than adaptation (Fig. 5C, teal). However, we observed that individual participants exhibited markedly different patterns of step length asymmetry during this phase (SI Appendix, Fig. S12). We quantified this observation by evaluating, for each participant, the number of strides in this phase with step length asymmetry below their own baseline CI (SI Appendix, Table S8). We used a density-based analysis to formally assess whether there were separate clusters in our data (see Methods). Indeed, the algorithm detected two separate clusters of participants: for 12 participants, between 38 to 60 strides were asymmetric (out of 60 total strides); for the other 8 participants, only 3 to 21 strides were asymmetric (Fig. 5D-E insets, and SI Appendix, Fig. S13; difference in strides between subgroups = 36.083 [29.917, 42.250], mean [CI]). As a control, we performed the same clustering analysis for Experiment 1, and did not find separate clusters for any of our measures of interest (SI Appendix, "Experiment 1 clustering analysis", Fig. S13-14, and Table S9-10).

This result indicates that 12 of 20 participants could not account for belt speed differences larger than that of adaptation, suggesting that they used a memory-based mapping mechanism (Fig. 5D). In contrast, 8 of 20 participants could account for these speeds, suggesting that they engaged a structure-based mapping mechanism (Fig. 5E).

**Figure 5. Experiment 2, Step length asymmetry. (A)** Experimental protocol, equivalent to that of Experiment 1 except for the Ramp Up & Down part shaded in teal, where the right speed was faster than in adaptation (ramped up to 2m/s and back down to 1.5m/s). **(B)** Step length asymmetry time course (entire group mean ± SE). Background shade represents belt speed difference. Phases (except ramp tasks) are truncated to the participant with fewest strides. **Inset:** Predictions for the step length asymmetry during the teal portion of the Ramp Up & Down task, for the memory-based (top) or structure-based (bottom) mapping hypotheses. **(C)** Zoomed-in Ramp Up & Down task (entire group mean ± SE). Step length asymmetry for strides taken at right speeds larger than adaptation is shown in teal. **(D-E)** Separate plots of the step length asymmetry in the Ramp Up & Down task for the subgroups of participants that walked asymmetrically (D, “memory-based”) versus symmetrically (E, “structure-based”) in the teal portion of the task (subgroups mean ± SE). **Insets:** circles represent individual participants’ number of strides, in the teal portion of the task, with step length asymmetry below their own baseline CI. Error bars depict subgroup mean ± SE. Subgroup assignment was performed by clustering on this measure.

We next aimed to exclude the possibility that participants in the structure-based subgroup may simply be faster at adapting to new perturbations than those in the memory-based subgroup and may be adapting to the new perturbations of the Ramp Up & Down rather than generating Δ motor output using a previously learnt structure. To this end, we evaluated learning rates during adaptation. We found that participants in the two subgroups adapted at similar rates (strides to plateau difference, structure – memory: 135.875 [-53.208, 329.708], mean [CI]; SI Appendix, Fig. S15-16), confirming that the different pattern of step length asymmetry in the Ramp Up & Down task reflects different types of mapping in the subgroups.

We considered that the mapping adjustments described here may or may not be *deliberate* (i.e., participants are trying to correct for the perturbation) or done with an *explicit strategy* (i.e., participants can accurately report a relevant strategy that would counter the perturbation) (Long et al., 2016; Roemmich et al., 2016). An example of an explicit strategy in visuomotor reaching adaptation is when participants report that they aimed to offset a visual rotation (Bond and Taylor, 2017; Taylor et al., 2014); conceivable examples in split-belt treadmill adaptation may be reports of “taking steps of similar length”, “stepping further ahead with the right foot” or “standing on the left foot for longer”. We tested whether participants could explicitly report changes to the gait pattern that specifically correct for the split-belt perturbation.

At the end of the experiment, participants were asked to report (in writing) if/how they had changed the way they walked during adaptation (note that this was a later addition to the protocol and was only collected in 16 of the 20 participants). We assessed reports by categorizing them in three steps: 1) did the report mention *any* deliberate changes? 2) were the changes *relevant* to adaptation? (i.e., did the report mention any gait metric contributing to the overall Δ motor output or step length asymmetry adaptation in any amount?) 3) was the response *accurate?,* as participants often reported strategies that they did not actually execute.

We summarize results from the questionnaire in Figure 6 (original responses are reported in SI Appendix, Table S11). We found that, while 13 participants reported *deliberate* changes, only 6 people mentioned *relevant* aspects of the walking pattern (in particular, participants mentioned “limping” or temporal coordination). Furthermore, only one of these participants reported an *accurate* gait parameter (“I tried to spend as much time leaning on my left leg as possible”), while the remainder of the relevant responses were inaccurate (e.g., “matched duration of standing on each foot”) or vague (e.g., “I adjusted as if I was limping”). The aspect of gait most frequently reported across participants was stability (also reported as balance, not falling, or controlling sway). This suggests that, while participants may deliberately adjust their body to feel more stable, they do not seem to explicitly strategize how to offset the perturbation. Thus, mapping tends to adjust aspects of the walking pattern that participants are not explicitly aware of controlling, suggesting that this differs from explicit strategies often observed in reaching.

In sum, in Experiment 2 we found that 1) participants are largely unable to explicitly describe adaptations to their walking pattern, and 2) some but not all participants can extrapolate their walking pattern to account for larger perturbations. This sheds light on how the stimulus-response mapping mechanism of walking adaptation may align with established mechanisms: it may differ from explicit strategies and resemble memory-based caching in some people and structural learning in others.

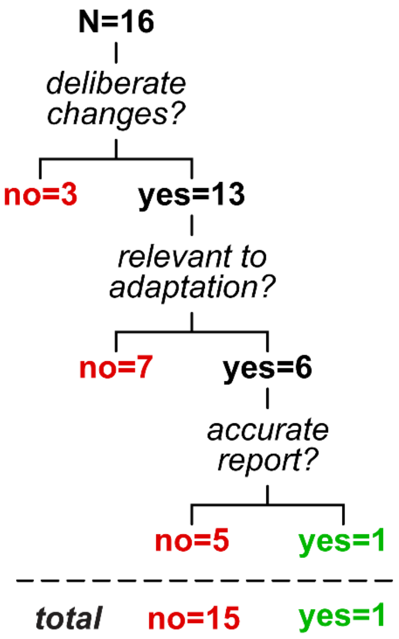

**Figure 6.** Summary of self-reported deliberate changes to the walking pattern in adaptation. Only one participant accurately described changes to the walking pattern that related to adaptation, while other responses were negative (i.e. no deliberate changes, 3 participants), irrelevant (7 participants), or inaccurate (5 participants).

## Discussion

In this study, we showed that locomotor adaptation involves two learning mechanisms: forward model recalibration and a stimulus-response mapping mechanism. The recalibration mechanism changes movement gradually and relates to the perceptual changes observed in locomotor adaptation. The mapping mechanism is flexible and, once learned, can change movement immediately to account for a range of belt speed configurations. Our data suggest that this mapping operates independently of explicit strategies, and that it can be memory-based or structure-based.

*Forward model recalibration of movement and perception*

In line with various adaptation studies, we showed that people recalibrated their perception in a way that reduced how perturbed they felt (Haith et al., 2008; Harris, 1963; Jensen et al., 1998; Moidell and Bedell, 1988; Sombric et al., 2019) – i.e., their perception of the leg speed difference diminishes (Jensen et al., 1998; Leech et al., 2018a; Rossi et al., 2019; Statton et al., 2018; Vazquez et al., 2015). The extent of the perceptual change correlated with the extent of the motor change that was achieved through forward model recalibration. Although previous research suggested that perceptual changes may be mediated by recalibration processes, the specific link to motor adaptation remained unclear ('t Hart and Henriques, 2016; Izawa et al., 2012; Sombric et al., 2019; Synofzik et al., 2008; Yavari et al., 2016; for a review, see Rossi et al., 2021a). Our findings demonstrate a direct relationship between perceptual and motor recalibrations, offering a novel approach to dissect forward model recalibration from stimulus-response mapping contributions to adaptation (SI Appendix, Supplementary Discussion 1).

Based on our findings, we suggest that forward model recalibration counters the perturbation in the motor and perceptual domains simultaneously – it adapts the walking pattern while realigning perception of leg speed. In support of this idea, perceptual realignment resembles a well-studied "sensory cancellation" phenomenon – where forward model predictions are used to filter redundant sensory information (Anderson et al., 2012; Blakemore et al., 1998). Forward model predictions may also be integrated with and sharpen proprioceptive estimates (Bhanpuri et al., 2013; Weeks et al., 2017), and may mediate a more complex integration of predicted and actual sensory signals contributing to perceptual changes. Consistent with the operation of a forward model thought to predict sensory consequences from efferent copies of motor commands (Haruno et al., 2001; Ito, 1989; Wolpert et al., 2001), perceptual changes are expressed during active and not passive movements (Sombric et al., 2019; Vazquez et al., 2015).

The cerebellum is thought to house forward models (Tanaka et al., 2020; Wolpert et al., 2001), and plays a role in motor adaptation (Bastian, 2011; Martin et al., 1996b; Morton and Bastian, 2006), perceptual realignment (Izawa et al., 2012; Statton et al., 2018; Synofzik et al., 2008; Yavari et al., 2016), as well as coordinating motor and sensory cortical activities (Lindeman et al., 2020; Popa et al., 2013). Hence, motor and perceptual changes with adaptation may reflect recalibration of forward models in the cerebellum processed downstream by motor and sensory cortices. Neural substrates are further discussed in SI Appendix, Supplementary Discussion 2.

*Flexible yet automatic stimulus-response mapping*

To the best of our knowledge, this is the first study to show that a stimulus-response mapping mechanism plays a role in walking adaptation. Unlike previous work employing a slow (10-minute) post-adaptation perturbation ramp down (Roemmich and Bastian, 2015), our “Ramp Down” task was quick (averaging 1 minute and 20 seconds) so that participants did not undergo washout (the aftereffect magnitude after the Ramp Down was comparable to that measured post-adaptation in prior work) (Reisman et al., 2005). This ruled out the possibility that changes in walking patterns were due to washout, reaffirming the presence of a flexible mapping mechanism that complemented the adjustments made by recalibration to produce a walking pattern well-suited to the current treadmill configuration (SI Appendix, Supplementary Discussion 3).

Our results also suggest that this mapping mechanism may operate independently of explicit control. In previous work where an explicit goal is given, adaptation mechanisms deployed in addition to forward model recalibration are consistently found to operate under deliberate control (Codol et al., 2018; Taylor et al., 2014; Taylor and Ivry, 2011) – including memory-based caching (Huberdeau et al., 2019; McDougle and Taylor, 2019) and structural learning mechanisms (Bond and Taylor, 2017; McDougle and Taylor, 2019). Mounting evidence indicates that explicit strategies do not play a strong role in split-belt walking adaptation where an explicit movement goal is not provided to the participants; for example, people do not adapt faster even after watching someone else adapt (Song et al., 2020). Strategic adjustments to the walking pattern can be temporarily elicited by providing additional visual feedback of the legs and an explicit goal of how to step. Yet, these adjustments disappear immediately upon removal of the visual feedback (Roemmich et al., 2016), and have no effect on the underlying adaptive learning process (Long et al., 2016; Malone and Bastian, 2010; Roemmich et al., 2016). Furthermore, performing a secondary cognitive task during walking adaptation does not affect the amount of motor learning (Hinton et al., 2020; Malone and Bastian, 2010; Rossi et al., 2021b; Vervoort et al., 2019). Here, we show that explicit strategies are not systematically used to adapt step length asymmetry and Δ motor output: the participants in our study either did not know what they did, reported changes that did not actually occur or would not lead symmetry. Only one person reported "leaning" on the left (slow) leg for as much time as possible, which is a relevant but incomplete description for how to walk with symmetry (SI Appendix, Supplementary Discussion 4).

Recalibration-adjusted perceptual information on the treadmill configuration may be relayed via sensory cortical areas and cerebellum to motor cortical areas, where it may be mapped to motor adjustments. This is supported by the interconnectivity between these substrates (Judd et al., 2021; Kandel et al., 2013; Sultan et al., 2012), and by the cortical contribution to the flexible control of walking (Drew and Marigold, 2015; Reisman et al., 2010) beyond voluntary control (Delval et al., 2020; Petersen et al., 2012). Mapping may also involve spinal control – which is rapid and automatic (Takakusaki, 2013) and can play a role in split-belt walking (Ogawa et al., 2014; Vasudevan et al., 2011) – but likely through connection with supraspinal structures due to the extensive training needed for plasticity within the spinal cord (Iturralde and Torres-Oviedo, 2019; Thompson et al., 2009) (SI Appendix, Supplementary Discussion 5).

In sum, the mapping mechanism combines the advantages of automaticity and flexibility (Huberdeau et al., 2015; Taylor et al., 2014; Taylor and Ivry, 2011). This is ecologically important for both movement accuracy (Uiga et al., 2020; Wong et al., 2008) and for walking safely in real-world situations, where we walk while talking or doing other tasks, and terrains are uneven (Clark, 2015; Paul et al., 2005; Wong et al., 2008) (SI Appendix, Supplementary Discussion 6).

*Mapping operates as memory-based in some people, structure-based in others*

Results from Experiment 2 highlight individual differences in the learning mechanisms underlying generalization to unexperienced belt speed differences (usage of the term "generalization" is discussed in SI Appendix, Supplementary Discussion 7). The generalization to novel perturbation sizes observed here is in line with previous suggestions of “meta-learning” in the savings of walking adaptation (i.e., faster relearning when exposed to a different perturbation) (Leech et al., 2018b; Malone et al., 2011). Generalization to *larger* perturbations after reaching adaptation was shown to be incomplete (Abeele and Bock, 2001; Lazar and Van Laer, 1968), and it was unclear whether the movement patterns had been *extrapolated* beyond what had been experienced, or rather was just the same as in adaptation. Surprisingly, we found 40%-60% divide in our participants regarding the capacity to extrapolate walking patterns to account for larger perturbations.

We suggest that structural learning may underlie the ability to extrapolate walking patterns and walk symmetrically for belt speed differences larger than adaptation, as seen in 8 of 20 participants. Indeed, generalization in reaching adaptation may rely on a process of structural learning (Bond and Taylor, 2017; Braun et al., 2009) (note, however, that this was tied to explicit aiming; Bond and Taylor, 2017). Our participants may have learned the scaling relationship between belt speed perturbation and the walking pattern needed to walk symmetrically and use this scaling to produce new walking patterns matching larger perturbations. In contrast, memory-based theories of mapping (Dassonville et al., 2001; Poggio and Bizzi, 2004; Van Vugt and Ostry, 2018; Wolpert et al., 2001) may explain why 12 participants walked asymmetrically for perturbations larger than adaptation, despite generalizing to smaller perturbations. Here, the walking patterns may be stored in memory during adaptation in association with the amount of perturbation they correct, and then retrieved during the ramp tasks. People transition through a range of walking patterns in adaptation (see gradual transition from baseline to adapted Δ motor outputs in Box 1 and Fig. 1), so storage of intermediate outputs may reflect generalization to smaller perturbations (while larger Δ motor outputs are not experienced). SI Appendix, Supplementary Discussion 8, discusses potential *interpolation* computations.

### *Conceptual model*

We propose a comprehensive conceptual model of walking adaptation that captures key behavioral properties uncovered in our study. We build upon standard models (Shadmehr and Krakauer, 2008; Smith et al., 2006) and introduce a mechanism and architecture for adaptive learning that accounts for the flexibility of the walking pattern and the relationship between motor and perceptual changes. This conceptual model is illustrated in Figure 7.

The perturbation $p$ represents the effect of the belt speed difference on movement or perception. Note that motor and perceptual quantities are represented in the same relative units (relative to the perturbation).

The light blue "recalibration" box represents the operation of the forward model recalibration mechanism: this mechanism is not flexible, so that it produces the same output $x_r$ for any perturbation $p$. The recalibration $x_r$ may project to both sensory integration and motor control areas (green and blue boxes), where it may be used to recalibrate perception and movement.

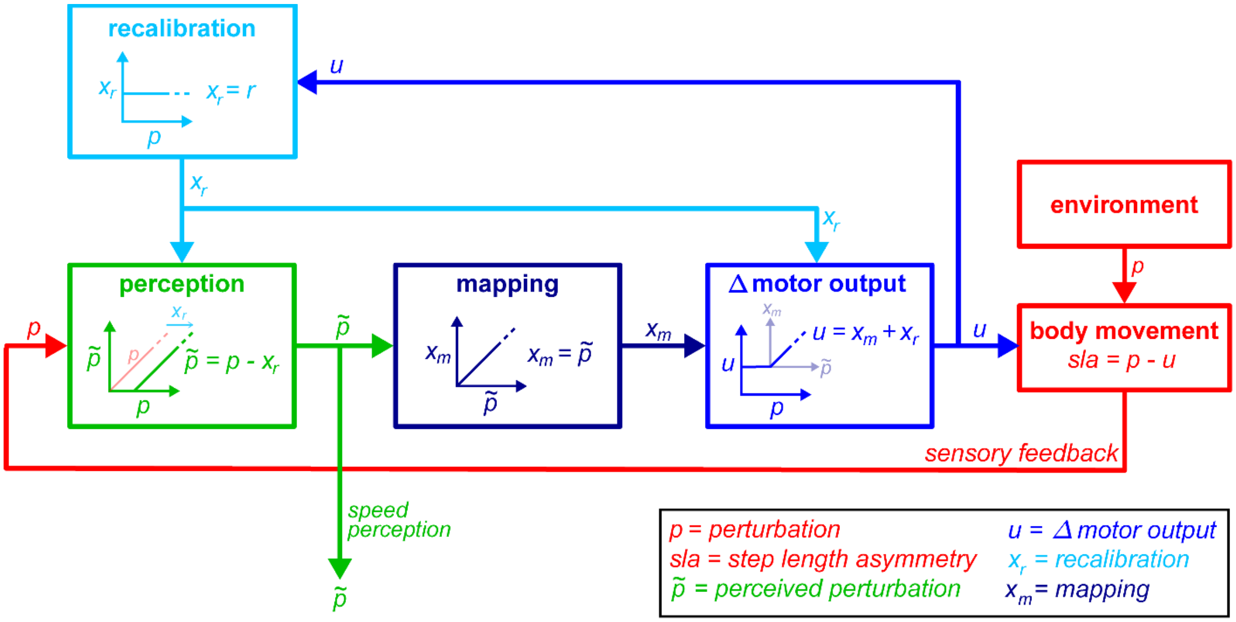

**Figure 7. Schematic model of adaptation.** Body movement depends on environment perturbations (red) and Δ motor output (blue). The Δ motor output is adjusted by recalibration (light blue) and mapping (dark blue) mechanisms, which perform different operations and are arranged in tandem. We propose the following architecture and flow: **(1. Recalibration)** The recalibration mechanism produces adjustment $x_r$ that is fixed regardless of perturbation size (light blue box, $x_r$ is constant for varying $p$). The *same* recalibration adjustment $x_r$ serves as an input to *both* areas responsible for conscious perception (green box) and Δ motor output (blue box). **(2. Perception)** Conscious perception is computed by cancelling out the recalibration adjustment from the actual sensory feedback (green box, perception of the belt speed difference perturbation $\tilde{p}$ is the difference between the actual speed difference $p$ and recalibration $x_r$). The perceived perturbation $\tilde{p}$ serves as an input to the mapping mechanism (dark blue box). **(3. Mapping)** The mapping mechanism produces adjustment $x_m$ that can vary in magnitude to appropriately account for the perceived perturbation $\tilde{p}$ (dark blue box, $x_m$ scales with $\tilde{p}$ and matches its magnitude). **(4. Δ Motor output)** The overall adjustment to Δ motor output is computed by adding the mapping adjustment $x_m$ and recalibration adjustment $x_r$. The corner in the Δ motor output versus perturbation profile arises because mapping is computed based on the perceived perturbation $\tilde{p}$ (not the actual perturbation $p$) and is only learnt for positive $\tilde{p}$ (the experienced direction). When the perturbation is perceived to be opposite than adaptation, even if it is not, mapping is zero and the Δ motor output is constant, reflecting recalibration adjustments only (blue box, when $\tilde{p} < 0$ and $p \geq 0$ the mapping adjustment $x_m$ is zero and $u = x_r$).

The green "perception" box represents the process of perceptual realignment. It receives sensory information regarding the perturbation, $p$, and the forward model recalibration output, $x_r$. It cancels out the portion of the perturbation predicted by the forward model, so that it is output is the perceived perturbation $\tilde{p} = p - x_r$. The perceived perturbation $\tilde{p}$ may serves both as signal for conscious speed perception (that captured by perceptual tests), and as an input to the stimulus-response mapping mechanism (dark blue box).

The dark blue "mapping" box represents the operation of the stimulus-response mapping mechanism. It receives the perceived perturbation signal $\tilde{p}$ and transforms into a motor adjustment appropriate to counter the perceived perturbation, $x_m = \tilde{p}$.

The blue "Δ motor output" box represent the computation of the Δ motor output. It receives the recalibration $x_r$ and mapping $x_m$ adjustments as inputs, and sums them to compute the Δ motor output $u = x_m + x_r$.

We propose the following series of computations. First, the recalibration is used to compute the perceived perturbation (green box). Second, the perceived perturbation is relayed to the mapping mechanism and used to compute the mapping-related motor adjustment (dark blue box). Third, recalibration-related and mapping-related motor adjustments are summed to produce the Δ motor output (blue box).

In conclusion, our model proposes three key features of the mapping mechanism that distinguish it from standard adaptation models. First, the mapping produces near-immediate changes to motor output in response to varying perturbations – contrasting the gradual changes proposed by state-space models (Herzfeld et al., 2014; Roemmich et al., 2016; Smith et al., 2006). Second, it accesses the perceived perturbation signal which integrates external sensory information with internal forward model state – contrasting the unimodal input proposed by optimal control (Shadmehr and Krakauer, 2008; Todorov, 2005). Third, the mapping operates automatically and is learnt with adaptation – contrasting the readily implementable explicit strategies modeled for reaching (Taylor and Ivry, 2011). These features enable automatic motor adjustments that complement recalibration to match varying perturbations, allowing our model to account for the observed symmetric walking across different treadmill speed configurations (SI Appendix, Supplementary Discussion 9).

*Limitations*

We aim to propose a framework for motor adaptation that is both parsimonious and based on a simple model. However, this simplicity inevitably comes with certain limitations. First, our model is descriptive and focused on capturing the novel features of the data revealed by the Ramp Down task, rather than the entire motor adaptation time series. Additionally, there may be alternative interpretations to our results, and a few mechanisms may underly the stimulus-response mapping observed here. As described in SI Appendix, Supplementary Discussion 10, there some evidence to suggest that walking adaptation involves optimizing motor commands to minimize energy costs (Finley et al., 2013; Sánchez et al., 2021, 2019, 2017). Therefore, our results may be explained by extending current optimal control models to account for our finding that motor commands are computed using internal state estimates and sensory feedback from the environment in tandem. Future work is needed to develop a generative model capable of capturing the features of adaptation presented here, and further characterize the nature of the stimulus-response mapping mechanism of automatic adaptation.

*Conclusions and future directions*

We here characterized two distinct learning mechanisms involved in walking adaptation, both of which are not under explicit control: recalibration – which leads to motor and perceptual aftereffects, and mapping – which only changes movement, does not contribute to aftereffects, and shows meta-learning to different perturbation sizes. Future work should explore whether the mapping mechanism described here could be of clinical significance: given the combined flexibility and automaticity, mapping has the potential to ameliorate known problems such as transitioning between walking environments (Sombric et al., 2017) and dual-tasking (Clark, 2015). Furthermore, our findings that different people adapt using different learning mechanisms (i.e., memory or structure-based mapping) highlights the importance of assessing individual characteristics of adaptation for both understanding of the neural mechanisms as well as translation to rehabilitation. Finally, as detailed in SI Appendix, Supplementary Discussions 9-11, the framework proposed here has important implications for the development of computational models of adaptation, and may help reconcile different findings related to savings, energetics, and sources of errors.

## Methods

### Participants

We recruited one-hundred adults (66 females, 23.6 ± 3.9 years old, mean ± SD) for this study, and we reanalyzed data from ten additional adults collected in a previous study (Leech et al., 2018a) (9 female, 21.3 ± 2.9 years old). The protocol was approved by the Johns Hopkins Institutional Review Board and participants provided written informed consent. Participants had no known neurological or musculoskeletal disorders, were naïve to split-belt walking, and participated in only one of the nine experiments.

### Data collection

Participants walked on a split-belt treadmill (Woodway, Waukesha, WI, USA) and belt speeds were controlled using a custom Vizard program (WorldViz). Kinematic data were collected using infrared-emitting markers (Optotrak, Northern Digital, Waterloo, ON, Canada) at 100Hz (SI Appendix, Supplementary Methods).

### Ramp tasks

For all ramp tasks of Experiments 1 and 2, the speed of the right belt was changed by 0.05m/s every 3 strides (defined below) during right leg swing. The baseline ramp of both experiments consisted of 7 increasing right speeds from 0.35m/s to 0.65m/s. The post-adaptation Ramp Down of Experiment 1 consisted of 21 decreasing right speeds from 1.5m/s to 0.5m/s. The post-adaptation Ramp Up & Down of Experiment 2 consisted of 41 total right speeds: 11 increasing speeds from 1.5m/s to 2m/s, followed by 30 decreasing speeds from 1.95m/s to 0.5m/s. The left belt speed was constant at 0.5 m/s for all ramp tasks. The treadmill was not stopped before the ramp tasks (people transitioned directly from the preceding walking blocks into the ramp) but was briefly stopped after each ramp task. We decided to not pause the treadmill between adaptation and the post-adaptation ramp tasks, and to make the tasks relatively quick – the Ramp Down was ~80 seconds on average – to limit unwanted forgetting and/or unlearning. We occluded vision and sound of the belt speeds using a cloth drape and headphones playing white noise.

In Experiment 1, a keyboard was placed on the treadmill handrail. In the post-adaptation Ramp Down task, participants were asked to press a button the first time they perceived the right belt to be (1) as fast as the left, and (2) faster than the left. In the baseline ramp, they press to report the right belt feeling (1) as fast as the left, and (2) slower than the left. This was explained prior to the experiment and the above prompts were displayed during the tasks on a television screen placed in front of the treadmill.

## Questionnaire

At the end of Experiment 2, participants answered this question on a computer: *Did you deliberately change how you walked to account for how fast the belts were moving? If so, describe how. Note: deliberately means that you thought about and decided to move that way. The question refers to the entire central ~20min walking block.* To ensure participants remembered what phase they were asked about, before adaptation we told participants that the following block will be called “central ~20min walking block”.

## Motor measures

We defined a stride as the period between two consecutive left heel strikes ($LHS_1$ to $LHS_2$), and computed the motor measures for each stride as in previous work (Finley et al., 2015; Sombric et al., 2017):

$$step\ length\ asymmetry = \frac{1}{stride\ length} * \Delta step\ length \quad (9)$$

$$perturbation = \frac{1}{stride\ length} * \overline{step\ time} * \Delta step\ velocity \quad (10)$$

$$\Delta\ motor\ output = \frac{1}{stride\ length} * \left(\Delta step\ position - \overline{step\ velocity} * \Delta step\ time\right) \quad (11)$$

The terms in the equations above are defined as follows:

$$\Delta step\ length = R\ step\ length - L\ step\ length \tag{12}$$

$$stride\ length = R\ step\ length + L\ step\ length \tag{13}$$

Where right ($R$) and left ($L$) step lengths are the anterior-posterior distance between the ankle markers of the two legs at right heel strike ($RHS$) and left heel strike ($LHS_2$) respectively.

$$\Delta step\ position = \left(R\ step\ position_{RHS} - L\ step\ position_{LHS_1}\right) - \left(L\ step\ position_{LHS_2} - R\ step\ position_{RHS}\right) \tag{14}$$

Where step position is the anterior-posterior position of the ankle marker, relative to average of the two hip markers, at heel strike of the same leg.

$$\Delta step\ time = R\ step\ time - L\ step\ time \tag{15}$$

$$\overline{step\ time} = \frac{R\ step\ time + L\ step\ time}{2} \tag{16}$$

Where left and right step times are the times from $LHS_1$ to $RHS$, and from $RHS$ to $LHS_2$ respectively.

$$\Delta step\ velocity = R\ step\ velocity - L\ step\ velocity \tag{17}$$

$$\overline{step\ velocity} = \frac{R\ step\ velocity + L\ step\ velocity}{2} \tag{18}$$

Where step velocity is the average anterior-posterior velocity of the ankle marker relative to average of the two hip markers, over the duration of the step (left: $LHS_1$ to $RHS$, right: $RHS$ to $LHS_2$).

In Experiment 2, we also computed the measure of "strides to plateau" using individual step length asymmetry data from the adaptation phase only. We first smoothed the data with a 5-point moving average filter. We then calculated the number of strides till five consecutive strides fell within the "plateau range", defined as the mean ± 1SD of the last 30 strides of adaptation (Malone and Bastian, 2010; Rossi et al., 2019).

### Clustering analysis

We tested to see if there were separate clusters of participants in our dataset. For Experiment 1, the measure used for clustering was the number of strides in the Ramp Down with step length asymmetry above the baseline CI. For Experiment 2, it was the number of strides in the first portion of the Ramp Up & Down (first 60 strides, teal in Fig. 5) with step length asymmetry below the baseline CI. For both experiments, "baseline CI" was computed separately for each participant as the 95% CI of the mean of step length asymmetry data in the second baseline tied-belt block (after the baseline ramp task; see Statistical Analysis).

We used the MATLAB "dbscan" density-based clustering algorithm (Ester et al., 1996) and automated the choice of parameters adapting previously published procedures (Naik Gaonkar and Sawant, 2013; Rahmah and Sitanggang, 2016) (SI Appendix, Supplementary Methods and pseudocode). The resulting algorithm does not require any user input; it automatically assigns participants to clusters (whose number is not predefined), or labels them as outliers, based solely on each participant's measure of interest.

### Data fitting

In Experiment 1, we fitted different models to individual participants' Δ motor output data in the Ramp Down. We developed and fitted our own recalibration + mapping model, presented in the Results section in Eq. 3. A reasonable upper limit for the parameter $r$ would be the magnitude of the perturbation in adaptation, but this motor measure differs across participants, which would lead to inconsistencies in the data fitting analysis. We therefore reformulated the recalibration and recalibration + mapping models to use a normalized parameter $r_{\text{norm}}$:

$$r = r_{\text{norm}} * p_{\text{plateau}} \tag{19}$$

Forward Model Recalibration: $u(p) = r_{\text{norm}} * p_{\text{plateau}}$ (20)

Recalibration + mapping: $u(p) = \begin{cases} p & , \text{ for } p \geq r_{\text{norm}} * p_{\text{plateau}} \\ r_{\text{norm}} * p_{\text{plateau}} , & \text{otherwise} \end{cases}$ (21)

Where $p_{\text{plateau}}$ is mean perturbation over the last 30 strides of adaptation, computed separately for each participant, $p$ is the perturbation in the Ramp Down, and $u$ is the modelled Δ motor output. We fitted the normalized parameter $r_{\text{norm}}$ using initial parameter value = 0.5 and bounds = [0, 1] for all participants. We then used Eq. 19 to compute the unnormalized value $r$.

For the recalibration only hypothesis, we fitted the dual state model defined as in Smith et al. (Smith et al., 2006):

net internal estimate: $x(k) = x_f(k) + x_s(k)$ (22)

fast state: $x_f(k+1) = A_f * x_f(k) + B_f * \big(p(k) - x(k)\big)$ (23)

slow state: $x_s(k+1) = A_s * x_s(k) + B_s * \big(p(k) - x(k)\big)$ (24)

Δ motor output: $u(k) = x(k)$ (25)

Where $k$ is the stride number, $p$ is the perturbation, $x$ is the net internal estimate of the perturbation, $x_f$ and $x_s$ are the fast and slow states contributing to the estimate. Participants generate Δ motor output $u$ that equals the internal estimate of the perturbation $x$ to minimize the predicted step length asymmetry. The model has four free parameters: $A_f$, $A_s$ are retention factors controlling forgetting rate, and $B_f$, $B_s$ are error sensitivities controlling learning rate. We fitted the model parameters using initial values $A_f$=0.92, $A_s$=0.99, $B_f$=0.1, and $B_s$=0.01 (as in

Roemmich et al., 2016), and constraints 0<$A_f$<$A_s$<1 and 0<$B_f$>$B_s$<1 (as defined by the model, Smith et al., 2006).

We also fitted two additional models that are prominent in literature: optimal feedback control (Izawa and Shadmehr, 2011; Shadmehr and Krakauer, 2008; Todorov, 2004) and memory of errors (Herzfeld et al., 2014). The details for these models are provided in SI Appendix, "Experiment 1 additional models".

We fitted each model to individual participants' Δ motor output data in the Ramp Down. Specifically, we found parameter values that minimize the residual sum of squares between the Ramp Down Δ motor output data and the modelled $u$. Dual state, optimal control, and memory of errors models involve hidden states that depend on the history of perturbations and are not expected to be zero at the start of the Ramp Down. To account for this, we simulated these models over the entire paradigm, but computed the residual sum of squares solely on the Ramp Down to ensure fair model comparison with the recalibration + mapping model. We used the *fmincon* MATLAB function with constraint and optimality tolerances tightened to 10^-20 (as in Roemmich et al., 2016).

Statistical analysis

Statistical tests were performed in MATLAB with significance level α=0.05 (two-sided). For Experiment 1, we performed within-group statistical analyses to compare the following measures to zero. For each participant and for each speed in the ramp tasks, we first averaged step length asymmetry over the 3 strides taken at that speed to obtain the measures listed in a) and b):

a) Step length asymmetry for each of the 7 speeds in the baseline ramp task (m=7)

b) Step length asymmetry for each of the 21 speeds in the Ramp Down task (m=21)

c) Difference in BIC between our model (recalibration + mapping) and each of the alternative models (dual state, optimal feedback control, memory of errors) (m=3)

d) Difference between $compensation_{\text{motor total}}$ or $compensation_{\text{motor recalibration}}$, and the upper and lower bounds of $compensation_{\text{perceptual}}$ (m=4)

Each item on the list represents a "family of related tests" (measures considered together for multiple comparisons), where "m" is the number of tests in the family. We computed bootstrap distributions of each measure by generating 10,000 bootstrapped samples of 20 participants (resampled with replacement from Experiment 1) and averaging the measure over participants in each sample (Efron and Tibshirani, 1994). We then computed confidence intervals (CI) corrected for multiple comparisons using the False Discovery Rate procedure (FDR) (Benjamini and Yekutieli, 2005). That is, for each family of related tests, we adjusted the significance level to $\alpha_{corr}=\alpha*\frac{R}{m}=0.05*\frac{R}{m}$, where "m" is the total number of tests and "R" is number of tests deemed significant for $\alpha = 0.05$. A test is significant if the CI does not overlap zero, indicating that the measure is significantly different from zero (Cumming and Finch, 2005; Efron and Tibshirani, 1994).

For both experiments, we computed within-participant CIs for the mean baseline step length asymmetry. For each participant, we generated 10,000 bootstrapped samples of N strides, resampled with replacement from 2-min baseline block following the ramp task, where N is the number of strides in this phase. We averaged step length asymmetry over strides in each sample and computed the 95% CI.

For Experiment 2, we performed between-group statistical analyses to assess whether the following measures differed between the memory-based and structure-based subgroups:

a) Number of strides to plateau (m=1)

b) Number of strides in teal portion of the Ramp Up & Down (first 60 strides) with step length asymmetry below the within-participant baseline CI (as described above) (m=1)

We generated 10,000 bootstrapped samples, each comprising 20 participants: 12 from the memory-based subgroup and 8 from the structure-based subgroup (resampled with replacement). For each sample "b", we averaged the measure of interest over participants resampled from each subgroup to obtain $\mu_{memory}(b)$ and $\mu_{structure}(b)$, and evaluated the difference of the means between the subgroups: $\Delta\mu(b)= \mu_{memory}(b)- \mu_{structure}(b)$. We computed the CI for this difference, correcting for multiple comparisons using FDR as explained for Experiment 1. Note that we report FDR-corrected CIs in the Results sections.

## Data Availability

*Note to reviewers: the dataset cited below is currently "private for peer review", and can be accessed by the alternative "reviewer URL" provided by the Dryad database:*

*https://datadryad.org/stash/share/Qa0pD7rMFT7kVgDeP-8PMYKBaCNuy4yx1VGIQlpVgvM*

All data and code used for the study have been deposited in: Rossi, Cristina (2023), Dataset for automatic learning mechanisms for flexible human locomotion, Dryad, Dataset, https://doi.org/10.5061/dryad.18931zd27.

## Acknowledgments

Supported by NIH grant 5 R37 NS090610 to AJB, American Heart Association predoctoral fellowship 20PRE35180131 to CR, and NIH grant K01 AG073467 to KAL. We thank Daniel Wolpert, Adrian Haith, Jonathan Tsay, and Amanda Therrien for helpful discussions and comments on data analysis.

**Author Contributions:** Study design & implementation: C.R., K.A.L., R.T.R., A.J.B.; Data acquisition & analysis: C.R., K.A.L.; Interpretation of results: C.R., K.A.L., R.T.R., A.J.B.; Writing & revision: C.R., K.A.L., R.T.R., A.J.B.

**Competing Interest Statement:** The authors declare no competing interests.

**Classification:** Biological Sciences: Neuroscience.

**Keywords:** Adaptation. Motor control. Motor learning. Human locomotion. Perception.

## Boxes

*Box 1. Split-belt walking adaptation paradigm and motor responses.*

There are three established motor measurements that are used to quantify split-belt walking adaptation (Finley et al., 2015; Reisman et al., 2005): “Perturbation” measures the effect of the split-belt treadmill on the stepping pattern — the total movement error we would see in the absence of adaptation. “Δ motor output” measures the extent that individuals compensate for the perturbation by changing their stepping pattern — how much they alter when (time) and where (position) they step on the treadmill with each foot. “Step length asymmetry” measures the remaining movement error — the difference between Δ motor output and perturbation (see Methods).

Box 1-Figure 1A illustrates the standard paradigm used in studies of split-belt adaptation, with abrupt transitions between tied-belt to split-belt phases, and Box 1-Figure 1B illustrates the standard motor response. In the “baseline” phase, the perturbation (red), Δ motor output (blue), and step length asymmetry (purple) are zero, reflecting equal belt speeds and symmetric walking. In the “adaptation” phase, the belt speeds are different and the perturbation is positive. Initially, this leads to movement errors observed as the negative step length asymmetry. The Δ motor output gradually adapts to compensate for the perturbation, so that the step length asymmetry returns to zero. In “post-adaptation”, the belt speeds are tied and the perturbation is again zero. Individuals exhibit initial movement errors called “aftereffects”: the Δ motor output mismatches the perturbation (it remains elevated) and step length asymmetry is positive (Leech et al., 2018a; Rossi et al., 2021a, 2019; Statton et al., 2018).

**Box 1 - Figure 1. Standard paradigm and measures. (A)** Treadmill belt speeds for the standard split-belt paradigm. **(B)** Schematic time course of standard motor measures of walking adaptation: step length asymmetry – a measure of error (solid purple), Δ motor output – a measure of compensatory spatial and temporal asymmetries (dotted blue), and perturbation – the effect of the speed asymmetry on the walking pattern (dashed red). In baseline, the belts are tied, and perturbation, Δ motor output, and step length asymmetry are all ~0. In adaptation, the right leg is faster than the left such that the perturbation is positive. The Δ motor output is still ~0 in early adaptation, causing step length asymmetry errors (negative purple line). By late adaptation the Δ motor output is adapted to match the perturbation, and step length asymmetry returns to ~0. Changes to Δ motor output persist in tied-belts post-adaptation, but the perturbation is ~0, causing step length asymmetry aftereffects (positive purple line).

*Box 2. Relevant methodologies and results from prior work on perceptual recalibration during locomotor learning.*

Box 2-Figure 1 illustrates selected portions of the “speed match” paradigm manipulation used in prior work and the respective step length asymmetry data (Leech et al., 2018a). The paradigm and data shown in Box 2-Figure 1A are consistent with that explained in Box 1 (with the addition of a brief tied-belt catch trial in adaptation). Note that participants walk with near-zero step length asymmetry by the end of adaptation and exhibit large aftereffects post-adaptation.

The study investigated “perceptual realignment”, where perception of speed becomes biased in adaptation, partially compensating for the speed difference so that it feels smaller. They measured perception of speed with “speed match” tasks: participants control the speed of the right belt and try to match it to that of the left belt (as described later in our Control experiments).

Left and right panels Box 2-Figure 1B depict the tasks performed before and after adaptation – top row shows belt speeds and bottom row shows step length asymmetry. Before adaptation, participants can accurately match the speeds and walk symmetrically at this near-tied-belt configuration (right and left speeds are ~equal and step length asymmetry is ~zero at the end of the “before adapt” task). After adaptation, participants overshoot the speed of the right belt and select a speed configuration that is biased towards that experienced in adaptation, perceiving this as “equal speeds” (the right speed is intermediate between the adaptation right speed and the target left speed at the end of the “after adapt” task). The key result is that participants walk with near-zero step length asymmetry at this configuration (“after adapt”, bottom row).

This suggests that participants may have learned to walk symmetrically at two distinct speed configurations: the adaptation configuration, and the configuration that they perceive as “equal speeds”. As illustrated in a later section (Results: Experiment 1 – Motor paradigm and hypotheses), this flexible behavior may be indicative of stimulus-response mapping mechanisms.

**Box 2 - Figure 1. Relevant results from Leech et al., 2018. (A)** Treadmill belt speeds and step length asymmetry time course, similar to that described in Box 1 – Figure 1. Vertical dashed gray lines indicate iterations of the speed match task, where participants adjust the speed of the right belt with a keypad to match it to the left. **(B)** Time courses of the belt speeds (top; orange = right, black = left) and step length asymmetry (bottom) in selected iterations of the speed match task. Left, "before adapt": last baseline task. Right, "after adapt": first post-adaptation task. Dotted horizontal lines depict the right speed (orange, top) and step length asymmetry magnitude (purple, bottom) at adaptation plateau (average over the last 30 strides). **(C)** Belt speed difference (top) and step length asymmetry (bottom) magnitudes at the end of the tasks shown in B. All curves show group mean ± SE, and all data is collected in the Leech at al. study (Leech et al., 2018a).

## Figure Legends

**Figure 4. Experiment 1, Perceptual Results. (A)** Top: perturbation data (red) and recalibration + mapping fit (blue); this is the same as Fig. 3C. Bottom: perceptual task button presses (green, group mean ± SE), as a function of belt speed difference. Right: measures of motor recalibration ("$r$") and total motor adaptation ("$u_{\text{plateau}}$"). **(B)** Perturbation compensation (normalized perceptual and motor measures of adaptation): $compensation_{\text{perceptual}}$ bounds (green), $compensation_{\text{motor total}}$ (dark blue), and $compensation_{\text{motor recalibration}}$ (light blue). **(C-D)** Individual participants' $compensation_{\text{motor recalibration}}$ versus $compensation_{\text{perceptual}}$ (first or second button press). Solid black: least squares line. Dashed gray: unity line.

**Figure 5. Experiment 2, Step length asymmetry. (A)** Experimental protocol, equivalent to that of Experiment 1 except for the Ramp Up & Down part shaded in teal, where the right speed was faster than in adaptation (ramped up to 2m/s and back down to 1.5m/s). **(B)** Step length asymmetry time course (entire group mean ± SE). Background shade represents belt speed difference. Phases (except ramp tasks) are truncated to the participant with fewest strides. **Inset:** Predictions for the step length asymmetry during the teal portion of the Ramp Up & Down task, for the memory-based (top) or structure-based (bottom) mapping hypotheses. **(C)** Zoomed-in Ramp Up & Down task (entire group mean ± SE). Step length asymmetry for strides taken at right speeds larger than adaptation is shown in teal. **(D-E)** Separate plots of the step length asymmetry in the Ramp Up & Down task for the subgroups of participants that walked asymmetrically (D, "memory-based") versus symmetrically (E, "structure-based") in the teal portion of the task (subgroups mean ± SE). **Insets:** circles represent individual participants' number of strides, in the teal portion of the task, with step length asymmetry below their own baseline CI. Error bars depict subgroup mean ± SE. Subgroup assignment was performed by clustering on this measure.

**Box 1 - Figure 1. Standard paradigm and measures. (A)** Treadmill belt speeds for the standard split-belt paradigm. **(B)** Schematic time course of standard motor measures of walking adaptation: step length asymmetry – a measure of error (solid purple), Δ motor output – a measure of compensatory spatial and temporal asymmetries (dotted blue), and perturbation – the effect of the speed asymmetry on the walking pattern (dashed red). In baseline, the belts are tied, and perturbation, Δ motor output, and step length asymmetry are all ~0. In adaptation, the right leg is faster than the left such that the perturbation is positive. The Δ motor output is still ~0 in early adaptation, causing step length asymmetry errors (negative purple line). By late adaptation the Δ motor output is adapted to match the perturbation, and step length asymmetry returns to ~0. Changes to Δ motor output persist in tied-belts post-adaptation, but the perturbation is ~0, causing step length asymmetry aftereffects (positive purple line).

**Box 2 - Figure 1. Relevant results from Leech et al., 2018. (A)** Treadmill belt speeds and step length asymmetry time course, similar to that described in Box 1 – Figure 1. Vertical dashed gray lines indicate iterations of the speed match task, where participants adjust the speed of the right belt with a keypad to match it to the left. **(B)** Time courses of the belt speeds (top; orange = right, black = left) and step length asymmetry (bottom) in selected iterations of the speed match task. Left, “before adapt”: last baseline task. Right, “after adapt”: first post-adaptation task. Dotted horizontal lines depict the right speed (orange, top) and step length asymmetry magnitude (purple, bottom) at adaptation plateau (average over the last 30 strides). **(C)** Belt speed difference (top) and step length asymmetry (bottom) magnitudes at the end of the tasks shown in B. All curves show group mean ± SE, and all data is collected in the Leech at al. study (Leech et al., 2018a).

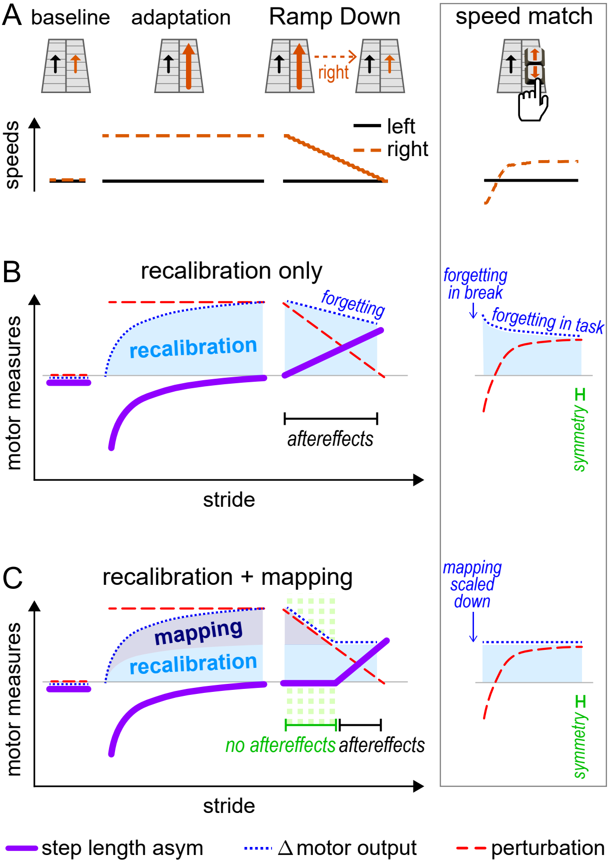

A
baseline
adaptation
Ramp Down
right
speed match
speeds
left
right
B
recalibration only
recalibration
forgetting
aftereffects
forgetting in break
forgetting in task
symmetry
motor measures
stride
C
recalibration + mapping
mapping
recalibration
no aftereffects
aftereffects
mapping scaled down
symmetry
motor measures
stride
step length asym
Δ motor output
perturbation

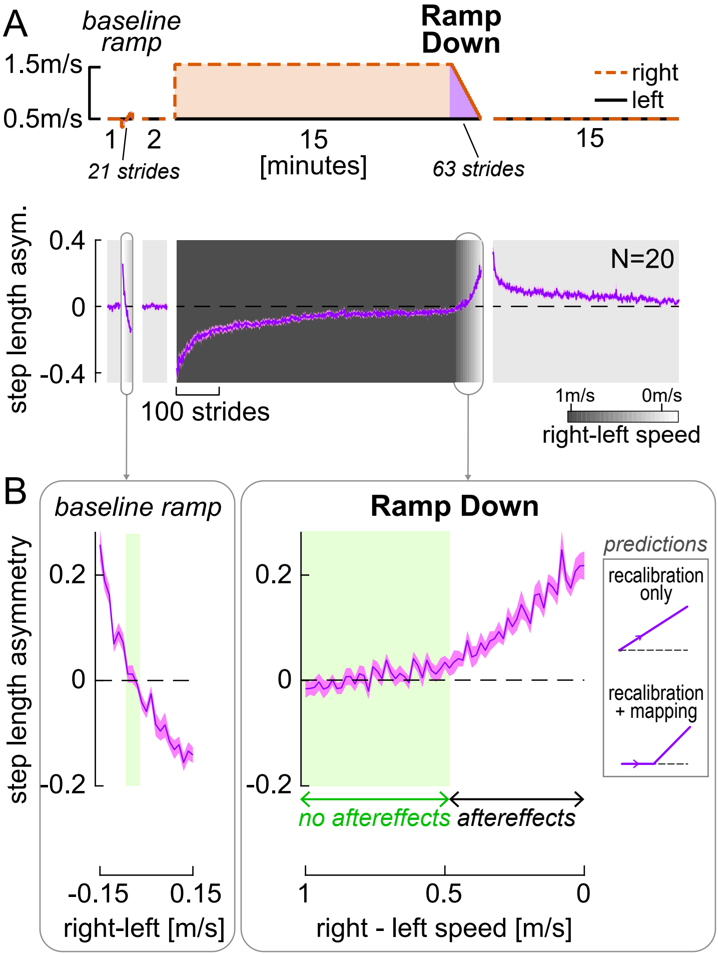

A
baseline ramp
Ramp Down
1.5m/s
0.5m/s
right
left
1
2
15
[minutes]
15
21 strides
63 strides
step length asym.
0.4
0
-0.4
N=20
100 strides
1m/s
0m/s
right-left speed
B
baseline ramp
step length asymmetry
0.2
0
-0.2
-0.15
0.15
right-left [m/s]
Ramp Down
0.2
0
-0.2
no aftereffects
aftereffects
1
0.5
0
right - left speed [m/s]
predictions
recalibration only

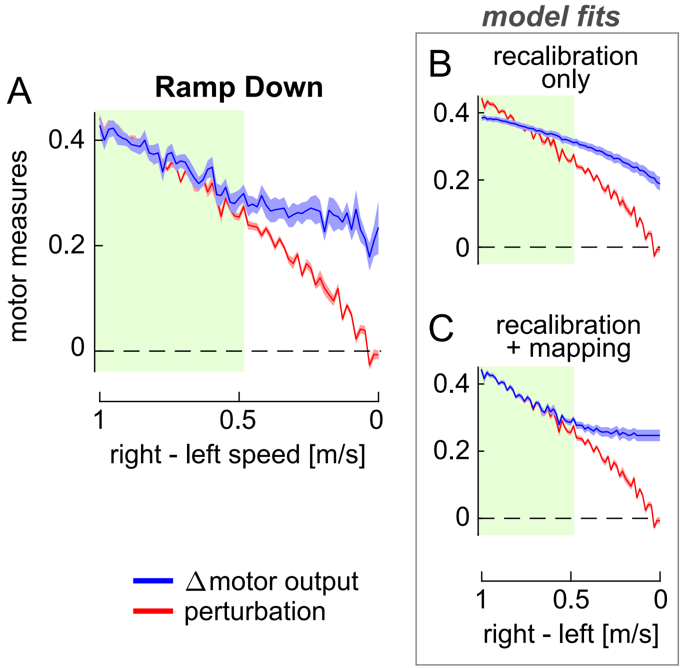

recalibration + mapping model fits
A
Ramp Down
motor measures
0.4
0.2
0
1
0.5
0
right - left speed [m/s]
B
recalibration only
0.4
0.2
0
C
recalibration + mapping
0.4
0.2
0
1
0.5
0
right - left [m/s]
Δ motor output
perturbation

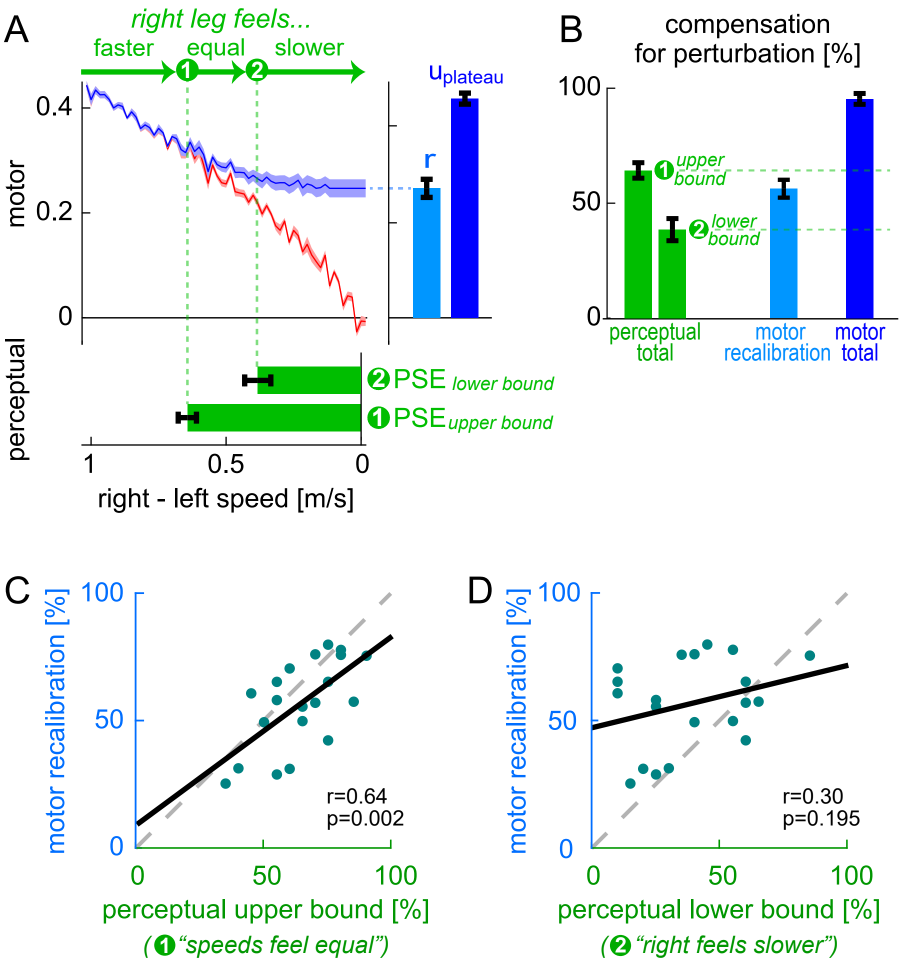

A
right leg feels...
faster
equal
slower
motor
0.4
0.2
0
perceptual
$u_{plateau}$
r
2 PSE lower bound
1 PSE upper bound
1
0.5
0
right - left speed [m/s]
B
compensation
for perturbation [%]
100
50
0
1 upper bound
2 lower bound
perceptual total
motor recalibration
motor total
C
motor recalibration [%]
perceptual upper bound [%]
(1 "speeds feel equal")
r=0.64
p=0.002
D
motor recalibration [%]
perceptual lower bound [%]
(2 "right feels slower")
r=0.30
p=0.195

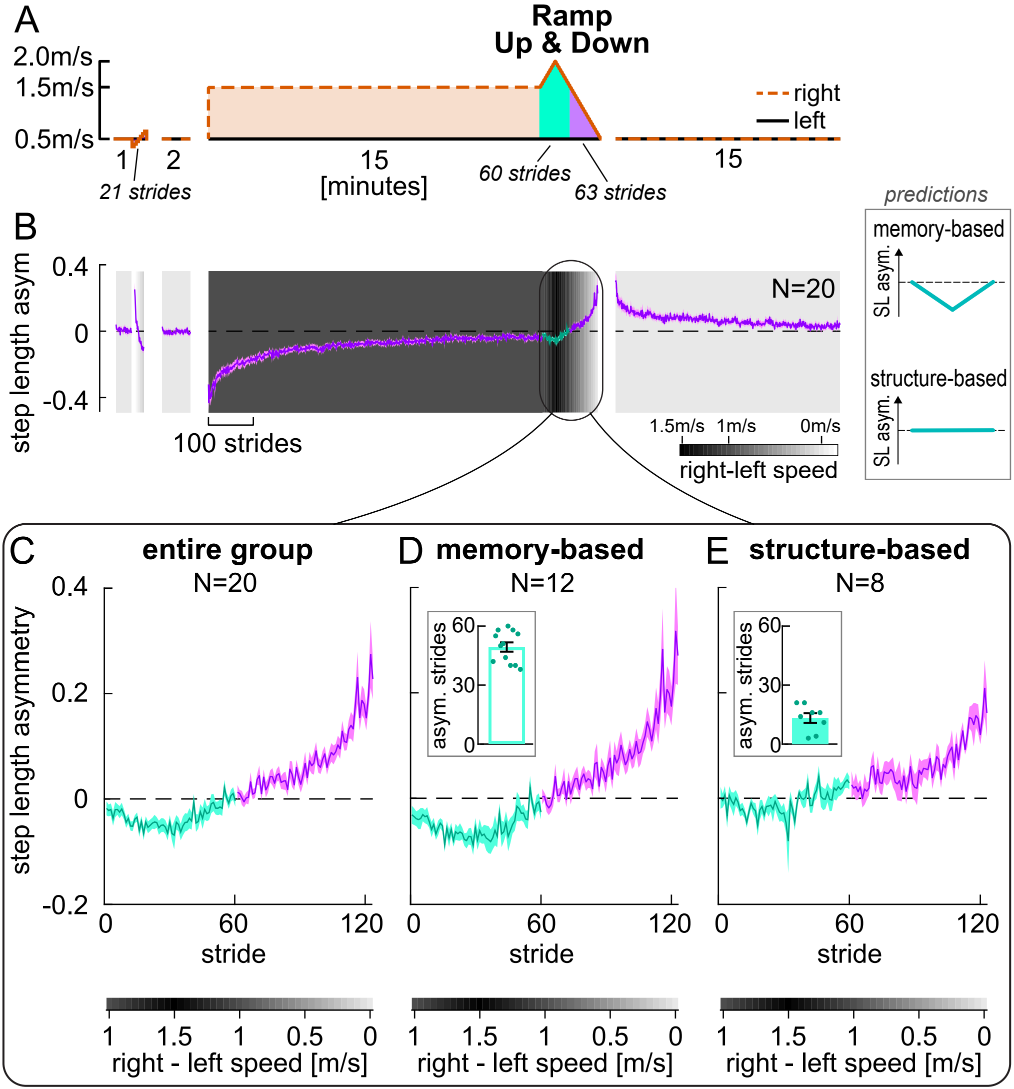

A
Ramp
Up & Down
2.0m/s
1.5m/s
0.5m/s
right
left
1
2
15
[minutes]
15
21 strides
60 strides
63 strides
B
step length asym
0.4
0
-0.4
N=20
100 strides
1.5m/s
1m/s
0m/s
right-left speed
predictions
memory-based
SL asym.
structure-based
SL asym.
C
entire group
N=20
step length asymmetry
0.4
0.2
0
-0.2
0
60
120
stride
1
1.5
1
0.5
0
right - left speed [m/s]
D
memory-based
N=12
asym. strides
60
30
0
0
60
120
stride
1
1.5
1
0.5
0
right - left speed [m/s]
E
structure-based
N=8
asym. strides
60
30
0
0
60
120
stride
1
1.5
1
0.5
0
right - left speed [m/s]

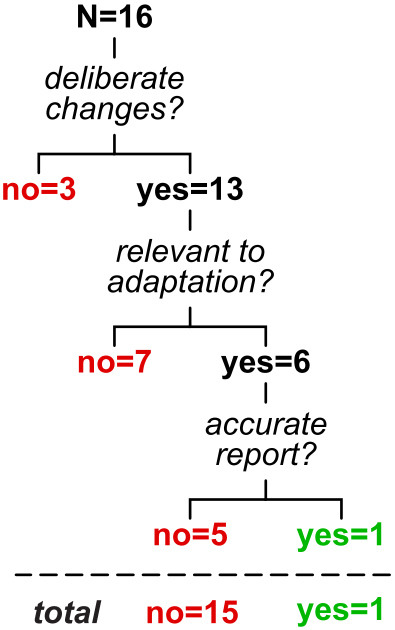

N=16
deliberate changes?
no=3
yes=13
relevant to adaptation?
no=7
yes=6
accurate report?
no=5
yes=1
total
no=15
yes=1

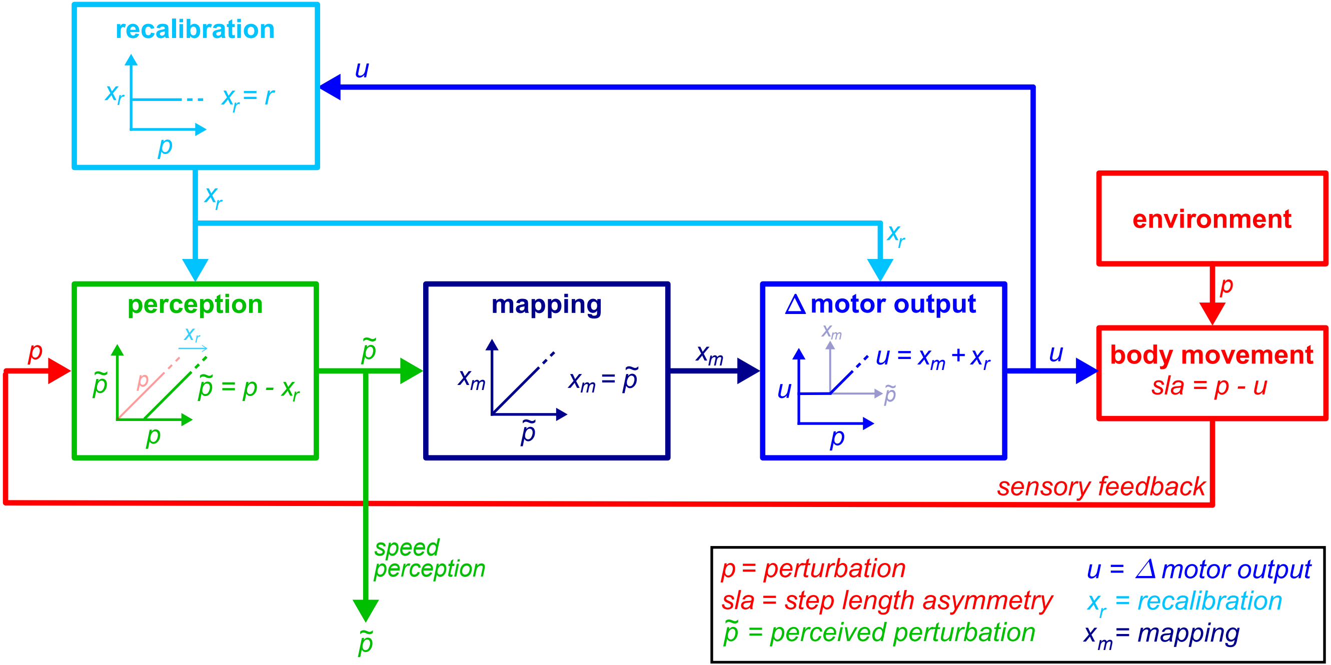

recalibration
$x_r$
$x_r = r$
p
u
$x_r$
$x_r$
environment
p
perception
$x_r$
$\tilde{p}$
p
$\tilde{p} = p - x_r$
p
p
$\tilde{p}$
mapping
$x_m$
$x_m = \tilde{p}$
$\tilde{p}$
$x_m$
Δ motor output
$x_m$
$u = x_m + x_r$
u
$\tilde{p}$
p
u
body movement
sla = p - u
sensory feedback
speed perception
$\tilde{p}$
p = perturbation
sla = step length asymmetry
$\tilde{p}$ = perceived perturbation
u = Δ motor output
$x_r$ = recalibration
$x_m$ = mapping

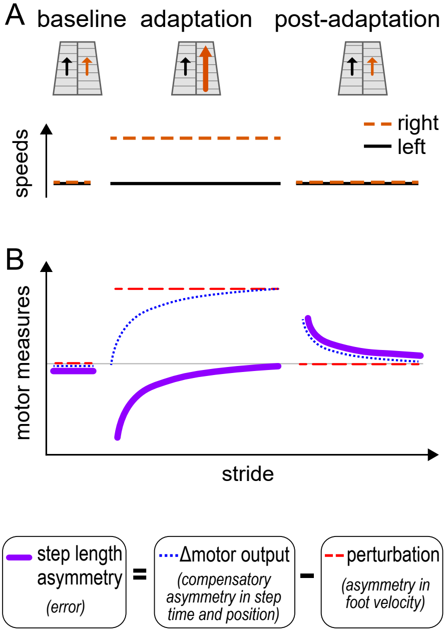

A
baseline
adaptation
post-adaptation
right
left
speeds
B
motor measures
stride
step length asymmetry
(error)
=
Δmotor output
(compensatory asymmetry in step time and position)
-
perturbation
(asymmetry in foot velocity)

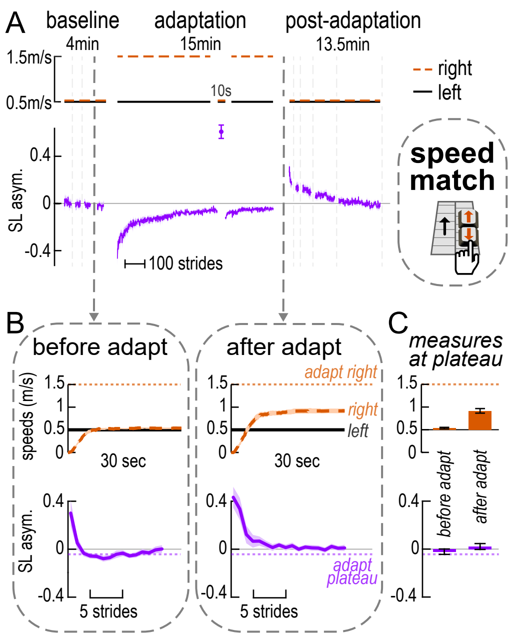

A
baseline
4min
adaptation
15min
post-adaptation
13.5min
1.5m/s
0.5m/s
10s
right
left
0.4
0
-0.4
SL asym.
100 strides
speed match
B
before adapt
speeds (m/s)
1.5
1
0.5
0
30 sec
SL asym.
0.4
0
-0.4
5 strides
after adapt
adapt right
right
left
30 sec
adapt plateau
5 strides
C
measures at plateau
before adapt
after adapt
