# Supplementary material for: Automatic learning mechanisms for flexible human locomotion
Source: bioRxiv. 2023 Sep 25:2023.09.25.559267. Preprint. [Version 1] doi: 10.1101/2023.09.25.559267 (PMC10557598; doi:10.1101/2023.09.25.559267)
Supplement: Supplementary file 1 [file media-1.pdf]

SI Appendix for: Automatic learning mechanisms for flexible human locomotion.

Authors: Cristina Rossi[a,b], Kristan A. Leech[c,d], Ryan T. Roemmich[b,e], Amy J. Bastian[a,b,*]

[a] Department of Neuroscience, The Johns Hopkins University School of Medicine, Baltimore, MD, 21205, USA; [b] Center for Movement Studies, Kennedy Krieger Institute, Baltimore, MD, 21205, USA; [c] Division of Biokinesiology and Physical Therapy, University of Southern California, Los Angeles, CA, 90033, USA; [d] Neuroscience Graduate Program, University of Southern California, Los Angeles, CA, 90007, USA; [e] Department of Physical Medicine and Rehabilitation, The Johns Hopkins University School of Medicine, Baltimore, MD, 21205, USA

*Corresponding author: Amy J. Bastian.

Email: bastian@kennedykrieger.org

This PDF file includes:

Supporting text:

Experiment 1 mechanisms characteristics (p. 2)

Control Experiments (p. 3)

Experiment 1 clustering analysis (p. 7)

Supplementary Methods (p. 8)

Supplementary Discussion (p. 10)

Pseudocode for the clustering analysis (p. 20)

Tables S1 to S10 (p. 21)

SI References (p. 31)

Figures S1 to S13 (p. 36)

Supporting Text

Experiment 1 mechanisms characteristics

A large body of work investigating forward model recalibration and stimulus-response mapping mechanisms of learning highlights key differences between these mechanisms and how they operate in adaptation. We illustrate how the mechanisms are thought to operate in Fig. S1A.

Adaptation is known to involve a mechanism of "*forward model recalibration",* characterized by predictive adjustments, where initially the *same* motor adjustment is used without regard of the current perturbation. Adjustments can only evolve gradually (i.e., over many stride-to-stride adjustments) (Bastian, 2008; Martin et al., 1996; Reisman et al., 2010; Wolpert et al., 2001), and the presence of post-adaptation aftereffects is a hallmark of forward model recalibration (Bastian, 2008; Martin et al., 1996; Reisman et al., 2010).

We asked whether adaptation may also involve a different *"stimulus-response mapping"* type of mechanism. Stimulus-response mapping mechanisms differ from forward model recalibration in several important ways:

1) They learn and store multiple motor adjustments for different perturbations.
2) They can readily switch between Δ motor outputs based on the current perturbation and can rapidly abandon the changes in motor output upon return to tied belts (in the case of split-belt treadmill adaptation).
3) The motor adjustments are mapped with the matching perturbation (accounting for the recalibration adjustments if both mechanisms are at play).
4) Because they can readily switch between Δ motor outputs, they do not result in lasting aftereffects post-adaptation.

Learning of a stimulus-response mapping may occur via different mechanisms (explicit strategies, structure learning…); we test these potential mechanisms directly in Experiment 2.

As mentioned, we expect that both recalibration and mapping mechanisms would contribute to adaptation, because this would account for the post-adaptation aftereffects that smaller than the initial adaptation errors (Kristan A. Leech et al., 2018; Rossi et al., 2021a, 2019; Statton et al., 2018). We considered the alternative hypothesis that only recalibration contributes to adaptation, and that the smaller aftereffects may be due to forgetting. We illustrate this in Fig. S1B; note that this hypothesis predicts that the aftereffects emerge immediately but grow more slowly.

Supporting Text

Control experiments

Results

The primary goal of the control experiments was to verify that the recalibration + mapping hypothesis demonstrated in Experiment 1 was robust to different paradigm manipulations. We tested 7 manipulations summarized in Fig. S6 (also see “protocols” below). We confirmed that $compensation_{\text{perceptual}}$ was significantly smaller than $compensation_{\text{motor total}}$ for all groups (Fig. S7A; statistical results in Table S3). We also replicated the expected pattern of step length asymmetry as a function of belt speed difference in the first post-adaptation speed match task (Fig. S7B and Table S2). Specifically, we found that step length asymmetry was ~0 at the end of the task (i.e. when the belt speeds felt equal) for all groups (statistical results reported in Table S2). Step length asymmetry was significantly positive (indicating aftereffects) at the start of the “ascend” tasks, when the right belt speed felt slower than the left (Short, Medium, and Long Ascend). Step length asymmetry was ~0 at the start of the “descend” task (i.e. when the right belt felt faster than the left) for the Medium Descend and Small Abrupt experiments. These results are consistent with what we observed in Experiment 1. Step length asymmetry was instead negative at the start of the “descend” task for the Short Descend and Small Gradual experiments. Note that, while this differs from the results of Experiment 1, a negative step length asymmetry is in the direction opposite to that of aftereffects (rather, it stems from incomplete adaptation), such that it does not contrast the recalibration + mapping hypothesis. In sum, the pattern of step length asymmetry versus belt speed in all controls experiments is consistent with the recalibration + mapping hypothesis.

Given different learning rates (Huberdeau et al., 2015; McDougle et al., 2016, 2015) of reaching adaptation mechanisms, we performed an exploratory analysis aimed at characterizing how the relative contribution of recalibration and mapping to adaptation evolves in time (Fig. S8). We specifically looked at this measure after 3, 15, or 30 minutes of adaptation (1.5:0.5m/s belt speeds), as we expected this window to capture the transition from incomplete to complete adaptation. We confirmed that Short and Medium Ascend had not yet fully adapted (CI of $compensation_{\text{motor total}}$ below 100%, statistics reported in Table S4) but Long Ascend did (CI overlapped 100%), and that the extent of adaptation was significantly larger for Medium Ascend than Short Ascend, and for Long Ascend than Medium Ascend (statistics in Table S5). We then compared the relative contribution of recalibration to adaptation between these experiments, and found that it did not change in time between 3, 15 and 30 minutes of adaptation (statistics in Table

S6). Hence, in contrast to reaching adaptation studies, our results suggest that the mechanisms of split belt adaptation may have similar learning rates. In particular, this suggests that the time course of mapping differs from that of explicit strategies found in reaching studies: strategies are acquired very fast upon exposure to the perturbation and their magnitude is later reduced once adaptation is complete, while mapping develops gradually and its magnitude is not reduced once fully adapted (as evidenced by the relative contribution of the mechanisms not differing between Medium and Long Ascend).

As reaching adaptation mechanisms are also modulated by error-size (Kagerer et al., 1997; Modchalingam et al., 2019; Neville and Cressman, 2018; Saijo and Gomi, 2010), we additionally explored how the relative contribution of recalibration and mapping is influenced by the magnitude and schedule of the belt speed perturbation (Fig. S8). We found that the relative contribution of recalibration was significantly *larger* when the belt speed difference perturbation was *smaller* (i.e. 0.4m/s instead of 1m/s; statistics in Table S6). While recalibration also appeared larger following a gradual instead of an abrupt adaptation schedule, this effect was not significant (Table S6). As a control, we finally confirmed that this measure did not differ between “ascend” and “descend” versions of the speed match task (Table S6).

In sum, the control experiments replicate the results of Experiment 1 for different testing methodologies and across a variety of paradigm manipulations, confirming that split-belt adaptation consistently involves both recalibration and mapping mechanisms. Our exploratory analysis further suggests that the relative contribution of these mechanisms to adaptation is affected by perturbation size, and does not change between 3 and 30 minutes of adaptation.

Protocols

All controls experiments were based on a similar general protocol based on previous work (Kristan A. Leech et al., 2018; Rossi et al., 2019; Statton et al., 2018; Vazquez et al., 2015), but varied in adaptation duration, belt speeds, and versions of the speed match task (Fig. S6). The left belt was always at the baseline speed throughout the entire experiment (Small Abrupt and Small Gradual: 0.4m/s; all other experiments: 0.5m/s). The right belt speed equaled the left during baseline, post-adaptation, and catch trial (which was administer two-third into adaptation to all experiments except for Small Gradual). Adaptation for the Small Gradual experiment lasted 15 minutes; the right speed was 0.4m/s for first 30 seconds, it was linearly increased till 0.8m/s for the next 14 minutes, and was 0.8m/s for the last 30 seconds. Adaptation for Small Abrupt lasted

15 minutes and the right speed was 0.8m/s. Adaptation lasted 3 minutes for Short Ascend & Descend, 15 minutes for Medium Ascend & Descend, and 30 minutes for Long Ascend, and the right speed was 1.5m/s. The treadmill was briefly stopped between every phase, including before/after the speed match tasks (with one exception: the treadmill was not stopped between the end of adaptation and first post-adaptation speed match task for the Small Gradual experiment only, as to avoid any abrupt exposure to the belt speed difference).

In the speed match tasks, the right belt speed was controlled by the participants, who were asked to "make the right belt move *as fast as* the left belt". Participants controlled the right speed with a keypad placed on the treadmill handrail; this had 4 buttons: large increment (+0.050, 0.055 or 0.065 m/s), small increment (+0.005 m/s), large decrement (-0.050 m/s), and small decrement (-0.005 m/s). The large increment varied with each task iteration (between the values reported above) as in previous work (Vazquez et al., 2015). The tasks lasted 30 seconds and a time-bar was displayed on a TV screen. Vision and sound of the treadmill were occluded as in Experiment 1. The "ascend" version of the speed match task started with the right belt stationary (0m/s); the "descend" version started with the right belt at the same speed as the end of adaptation (Fig. S6B). The "ascend" version was used in baseline for all experiments, and post-adaptation for Short Ascend, Medium Ascend and Long Ascend; the "descend" version was used for post-adaptation of the other experiments.

Data and statistical analysis

We focused on the first post-adaptation speed match task. PSE was computed as the belt speed difference at the end of the task. $compensation_{\text{perceptual}}$ and $compensation_{\text{motor total}}$ were computed and statistically compared within group (m=7) using the same analysis as in Experiment 1 (0.4m/s was used for Small Gradual PSE normalization). The same statistical procedure was used to compute the CI for $compensation_{\text{motor total}}$ within-group and compare it to 100% (m=7). We computed "recalibration contribution to adaptation" as $compensation_{\text{perceptual}}$ over $compensation_{\text{motor total}}$, and statistically evaluated the difference of the means between selected groups for this measure, using a bootstrap procedure analogous to that described for Experiment 2 (m=6). The same procedure was used to compare $compensation_{\text{motor total}}$ between selected groups (m=6), and compare PSE of Medium Ascend or Descend to that of Experiment 1 (m=2).

We formally evaluated step length asymmetry in the speed match task by evaluating the confidence interval for the group mean of the first (m=7) and last (m=7) strides, with a bootstrap

procedure analogous to that described for Experiment 1. For the time course plot of Fig. S7B, we *interpolated* step length asymmetry as a function of speed in the task (because speeds differ across participants). We used locally weighted linear regression (MATLAB '*smooth'*, method = 'lowess', span = 20), with query speeds ranging from minimum to maximum speed at which a stride was taken (average across participants) in steps of 5 mm/s. The speed of a stride refers to the average speed for the duration of that stride; similarly, when multiple strides were taken at the same speed, the average step length asymmetry over those strides was used for our analysis.

Supporting Text

Experiment 1 clustering analysis

The primary clustering analysis was designed to match that of Experiment 2 (see Supplementary Methods). For each participant, we evaluated the number of strides in the Ramp Down with step length asymmetry above their own baseline CI (2-min baseline block), indicating aftereffects (all CI reported in Table S8). Using the same density-based analysis as Experiment 2, we found no separate clusters in this measure (Fig. S10A). Nevertheless, the analysis detected two outlier participants (Fig. S10B). Because these participants showed aftereffects in *fewer* strides than the rest of the group, and walking without aftereffects for multiple speeds is evidence for mapping, we did not believe these outliers affected our main finding that adaptation involves mapping in addition to recalibration. We formally verified this by recomputing all statistical analyses of Experiment 1 without these outliers; this confirmed that none of the statistical results were affected by outliers (statistical results in Table S9).

As a further control, we repeated the clustering analysis using all the different measures evaluated in Experiment 1 (Fig. S11): BIC difference between the recalibration + mapping function, and each of the other functions (recalibration only, mapping only, dual state); “r” parameter; $compensation_{\text{motor total}}$; and PSE upper and lower bounds. We did not find evidence of separate clusters of participants using any of these measures.

Supporting Text

Supplementary Methods

Experimental setup

Participants walked with one foot on each belt of the treadmill; a thin wooden divider was placed between the belts to avoid stepping on the opposite belt. For safety, participants wore a non-weight-bearing safety harness, and held on to the treadmill handrail when the treadmill was started or stopped, but were instructed to let go and cross their arms as soon as they began walking. To ensure participants' comfort, the treadmill was briefly stopped between each phase of the paradigm (with the exception of the transitions from walking blocks to ramp tasks – i.e., the treadmill was not stopped before the ramp up or ramp down).

Infrared-emitting markers were placed bilaterally over the toe (fifth metatarsal head), ankle (lateral malleolus), knee (lateral femoral epicondyle), hip (greater trochanter), pelvis (iliac crest), and shoulder (acromion process).

Clustering analysis

We used the MATLAB "dbscan" algorithm (Ester et al., 1996) - a density-based clustering algorithm that clusters data points based on their relative Euclidean distance (here, the data points are the individual participants' measures described in the manuscript). The algorithm does not take number of clusters as an input, instead it takes the following two inputs: 1) Epsilon, i.e. the maximum distance between data points for them to be considered neighbors, 2) MinPts, i.e. the minimum number of data points in a neighborhood for the neighborhood to be labelled a cluster. A neighborhood is an ensample of data points where each data point is a neighbor of at least one other data point in the neighborhood (i.e. each data point has a distance < Epsilon to at least one other data point in the neighborhood). Data points in neighborhoods whose size is less than MinPts are labelled as outliers.

In order to ensure the clustering analysis would not depend on our choice of parameters, we adapted previously developed algorithms (Naik Gaonkar and Sawant, 2013; Rahmah and Sitanggang, 2016) to automate the selection of Epsilon and MinPts. The original algorithm takes one parameter 'k' as an input, where 'k' can be an integer in the range of 1 to number of participants. To be fully unbiased, we here run the algorithm for all potential values of 'k' (ranging from 1 to 20), and develop a methodology to automatically select the best set of parameters out of the 20 'k' iterations. We provide pseudocode for the full algorithm below – in the SI Appendix

section "Pseudocode for the clustering analysis" – and explain here the general steps involved in the algorithm.

The first step is to obtain a set of epsilon-MinPts pairs to be tested. Potential epsilons are estimated based on the average distance of each participant to their closest 'k' neighbors. The algorithm computes the average distance measure and sorts it in ascending order across participants. It then detects locations where there is a sharp increase in average distance measure (specifically, where slope change – or concavity - is greater than 1% of the average slope). The values of the average distance measure at these locations are taken as potential epsilons to be evaluated. The potential epsilons are sorted by the average distance concavity (so that those obtained from locations with the sharpest change in slope are evaluated first). For each potential epsilon, the associated MinPts parameter is computed as the average number of neighbors that participants would have if that epsilon was used.

The second step is to select the most appropriate epsilon-MinPts pair for the current 'k' from the shortlisted options. First, epsilon-MinPts pairs are eliminated if MinPts is less than 2 (i.e. single participants could be detected as a standalone group) or more than 10 (i.e. clusters would be forced to have more than 10 people, hence it would be impossible for the algorithm to detect more than one group). The algorithm then runs dbscan for each remaining pair of epsilon-MinPts. The output is taken to be the first parameter pair that results in the smallest number of outliers.

The third step occurs after steps 1 and 2 are repeated for all 'k' values, and its goal is to select the overall best epsilon-MinPts pair across all 'k' iterations. First, it shortlists epsilon-MinPts pairs that resulted in the smallest number of outliers. Among these, it selects the epsilon-MinPts pair that led to the smallest number of clusters. This provided unique solutions on our data.

Supporting Text

<u>Supplementary Discussion</u>

(1) Experiment 1, lack of correlation between motor recalibration and the second button press in the perceptual task

We found that the motor recalibration parameter $compensation_{\text{motor recalibration}}$ was correlated to the first button press in the Ramp Down perceptual task (pressed when the speeds first felt equal) but not to the second button press (pressed when they no longer felt equal). The task is based on the psychophysical method of limits (Gescheider, 1997), which is known to be prone to response biases where participants are more likely to repeat the same response or perceptual judgment if they are uncertain. In our task, participants may press the button the second time only when they feel *fairly* certain that the right leg is slower than the left (Camacho et al., 2015; Gescheider, 1997). In particular, the first button press may reflect the point of subjective equality (participants feel that there is a 50/50 chance of what leg is faster), while the second button press may additionally reflect the just noticeable difference (participants feel that the chance of the right leg being slower than the left is some percentage higher than 50%). This may explain the lack of correlation between the second button press and motor recalibration; however, future work is needed to fully dissect if this is the case.

(2) Mapping mechanism differs from explicit strategies

Additional evidence for the automaticity of the mapping mechanism comes from the Control experiments. In contrast to explicit strategies in reaching adaptation (Huberdeau et al., 2015; Taylor et al., 2014; Taylor and Ivry, 2011), the learning rate of mapping seems to be similar to that of recalibration: the relative contributions of recalibration and mapping do not change with adaptation duration (for 3, 15 versus 30 minutes). Hence, mapping takes time to learn, supporting that it is an acquired motor program rather than a voluntary ad-hoc adjustment.

Finally, we note that the questionnaire responses from Experiment 2 included 4 reports of pressure or weight. Previous work shows some kinetic measures may relate to step length asymmetry (Hirata et al., 2019; Lauzière et al., 2014), but we could not verify any relationship here as we did not collect kinetic data. Importantly, reports were not specific, and they were inconsistent across participants (half reported larger force on the left side and half on the right side). Therefore, we do not believe that kinetic-related reports affect our findings that mapping differs from explicit aiming strategies. Other measures that were not considered related to step

length asymmetry adaptation were stride length (which does not adapt, (Reisman et al., 2005)) and mediolateral balance.

Future work is needed to understand whether deliberate strategies only contribute to adaptation in paradigms that give participants explicit movement goals.

(3) What are the benefits of automaticity?

There are several benefits associated with automatic control of movement: First, it does not compete for cognitive resources that are often needed for simultaneous tasks (e.g. walking while talking, listening, manipulating objects…) (Al-Yahya et al., 2011; Clark, 2015; McFadyen et al., 2017). Second, it may process and respond to environmental stimuli faster than voluntary control (Clark, 2015; Pinto et al., 2011; Schneider and Chein, 2003; Wakatsuki and Yamada, 2020; Welchman et al., 2010). Third, in contrast to automatic control, deliberate control can be detrimental to motor performance across a variety of tasks, including walking (Uiga et al., 2020; Young et al., 2020), goal-directing hand movements (Masters et al., 1993; Wan and Huon, 2005), and real world sports (Hardy et al., 2001; Masters et al., 1993) (reviewed in (Masters and Maxwell, 2008)). For example, paying conscious attention to walking may be associated with walking slower, taking shorter steps and spending more time in double-support (Young et al., 2020). Finally, beyond performance, automaticity of walking is a matter of safety: reduced automaticity is consistently associated with increased risk of falls in older adults (Clark, 2015; Fasano et al., 2012; Hyndman and Ashburn, 2004; Paul et al., 2005; Wong et al., 2009).

Mapping may be safer than either recalibration or explicit control at responding to walking perturbations: its flexibility may reduce the time spent limping and hence in an unstable walking state (Buurke et al., 2018; Darter et al., 2018) and its automaticity may reduce the risk of falls (Clark, 2015; Paul et al., 2005; Wong et al., 2008). In line with this idea, we find that the relative contribution of mapping to walking adaptation is relatively larger when the split perturbation is larger, a condition that may be more unstable and feel more hazardous.

In sum, our finding that walking adaptation is an automatic process may be ecologically beneficial for both allocation of resources and movement accuracy. In support of this idea, the prefrontal cortex (traditionally associated with explicit motor control) (Goldman-Rakic, 1987; Miller and

Cohen, 2001) becomes less active with split-belt adaptation, and greater deactivation is associated with more symmetric strides (Hinton et al., 2019).

(4) Features of the mapping mechanism

As discussed in the main text, the mapping-related adjustment to motor output, x_m, is computed as a function of the *perceived* perturbation $\tilde{p}$ (Fig. 7, dark blue box). Hence, its coordinate system must be transformed to *actual* perturbation p in the Δ motor output box. This change in coordinate system is key to represent the sharp "corner" in the Δ motor output data of the Ramp Down (Fig. 3-4): Mapping (x_m) scales with the perturbation for the first part of the task, when the right leg *feels faster* than the left ($\tilde{p}$ > 0, compare to Fig. 4). Mapping is instead zero (and recalibration x_r is the sole contributor to the Δ motor output u) for the second part of the task, when the right leg *feels slower* than the left ($\tilde{p}$ < 0). This change in mapping behavior occurs when the perturbation *feels zero* but is *actually not* ($\tilde{p}$ = 0 but p > 0; recall the right leg is faster than the left throughout the Ramp Down). This is a strong indicator that mapping uses *recalibrated* perception signals $\tilde{p}$ instead of actual perturbation signals p.

Note that our findings can only be explained by a mapping mechanism that only generalizes across perturbations "of the same sign" – i.e. to perturbations that feel in the same direction as adaptation ($\tilde{p}$ > 0). It does not generalize to perturbations that feel in the opposite direction ($\tilde{p}$ < 0, right leg feels slower than left). This is evidenced by the zero mapping-related adjustment for these configurations. The "signed" generalization observed here is in line with previous observations of interference between opposite perturbations (Bock et al., 2001; Krakauer and Shadmehr, 2006).

Similarly, our findings indicate that mapping "competes" to account the same perturbation signal as recalibration, at least in part (discussed more in Supplementary Discussion 8). Specifically, our findings cannot be explained by recalibration and mapping separately learning from predefined sub-portions of the overall perturbation (e.g. controlled externally by gains, (Shadmehr and Krakauer, 2008)). If mapping was computed using a portion of the *actual* perturbation p, rather than *recalibrated* perception of the perturbation $\tilde{p}$, it would scale proportionally to the perturbation throughout the entire Ramp Down task. It would not show a sharp change in slope, because there

would no "sign change" of the input signal ($p > 0$ for entire Ramp Down task, and any predefined proportion of p would also be positive throughout).

Finally, we notice that mapping learns a relationship between perceived perturbation $\tilde{p}$ (*stimulus)* and appropriate motor adjustment x_m *(response)*, despite the fact that the stimulus differs between the time of memory storage and that of retrieval. That is, when people first experience Δ motor outputs in adaptation, their perception of the perturbation differs from that during the Ramp Down task (they perceive more of the perturbation as recalibration is not yet complete). This indicates that, even in people for whom mapping is memory-based, Δ motor outputs cannot be simply associated with the experienced stimulus (which is the simplest case of stimulus-response association mechanisms, (Dassonville et al., 2001; Poggio and Bizzi, 2004; Van Vugt and Ostry, 2018; Wolpert et al., 2001)). Rather, Δ motor outputs may be associated with the amount of perturbation they are able to correct, similar to previous reports of error-correction mappings (Van Vugt and Ostry, 2018).

(5) Cortical correlates of reaching adaptation in monkeys resemble the combined operation of recalibration-like and mapping-like mechanisms

Research in the cortical neural correlates of reaching adaptation offers insight into how mapping may be encoded (Chase et al., 2012; Sun et al., 2022). In particular, Chase et al. describes two adaptation mechanisms that are remarkably consistent with the framework proposed here (Chase et al., 2012). The first mechanism did not change the tuning functions of cortical motor cells, rather modulated an upstream input used by all these cells, hence modulating the output of all cells equally, which behaviorally leads to a fixed adjustment in motor output across environmental conditions. The second mechanism changed the weight or tuning direction of individual cortical cells, such that it could modulate Δ motor output differently across environmental conditions. Recalibration resembles the first mechanism – it is thought to learn outside of cortical areas and affect cortical motor control downstream in a fixed manner, while mapping resembles the second mechanism – it may learn in cortical motor areas, produce variable motor adjustments, and use inputs modulated upstream by the first mechanism.

(6) Usage of the term "generalization"

The term "generalization" has been used in adaptation literature to refer to a wide range of phenomena (e.g. transfer to different reaching locations (Berniker and Kording, 2008; Castro et al., 2011; Izawa and Shadmehr, 2011; Tanaka et al., 2012; Thoroughman and Shadmehr, 2000),

different movements (Morton and Bastian, 2004; Wang et al., 2011) or movement parameters (Joiner et al., 2011; Morton et al., 2001; Thoroughman and Shadmehr, 2000), opposite limb (Berniker and Kording, 2008; Morton et al., 2001), novel environments (Gregory et al., 2021; Hamzey et al., 2016; Kluzik et al., 2008; Mariscal et al., 2020; Torres-Oviedo and Bastian, 2010) …). Different generalization-like phenomena may involve separate mechanisms (Berniker and Kording, 2008; Tanaka et al., 2012). Here we use “generalization” to mean the capability to account for perturbations of different sizes than that experienced in adaptation. This phenomenon has at times been referred to as “meta-learning”. This type of generalization has been tested either immediately after adaptation in reaching (Abeele and Bock, 2001; Braun et al., 2009; Lazar and Van Laer, 1968), or after washout and re-exposure to perturbations (i.e. generalization related to savings) in adaptation of both walking (Kristan A Leech et al., 2018; Malone et al., 2011) and upper extremities movements (Bond and Taylor, 2017; Herzfeld et al., 2014; Seidler, 2004; Turnham et al., 2012).

(7) Potential interpolation computations of mapping

Memory-based mapping may also operate by storing in memory a limited number of Δ motor outputs – such as just the baseline and adaptation plateau output – and then interpolating between memories (Poggio and Bizzi, 2004). This process has at times been referred to as structure learning because of the computations involved in interpolation (Braun et al., 2009; Haruno et al., 2001), but it is different than structure-based mapping. Whether by direct memory retrieval or interpolation, the Δ motor output in memory-based mapping is produced entirely on internal motor memories, and external sensory stimuli are only used for selection of the appropriate Δ motor output. In contrast, structure-based mapping does not use memories of Δ motor outputs, rather computes Δ motor outputs anew from the external sensory stimuli.

(8) Errors driving recalibration and mapping may partially overlap and partially differ

As discussed in Supplementary Discussion 4, mapping competes at least in part for correcting the same error as recalibration. While there is competition for at least *some* of the error signals, this may not be the case for *all* signals driving mapping. We speculate that mapping may be competing with recalibration to correct for kinematic and kinetic parameters of the gait pattern. Instead, we speculate that there are signals primarily affecting mapping which may include energy optimization, as this has been suggested to be responsible for adaptation to step length

asymmetries larger than zero upon long exposures (Finley et al., 2013; Sánchez et al., 2021, 2019, 2017).

While our findings do not imply that the error signals driving recalibration must be in competition with what was corrected by mapping, the most parsimonious hypothesis is that recalibration is driven by at least some of the same errors. The source of errors driving recalibration has been long object of discussion, where some studies suggest recalibration is driven by movement errors (Smith et al., 2006), others by sensory prediction errors (Lee et al., 2018; Tseng et al., 2007), and yet others by a combination of the two (Haith and Krakauer, 2013; Shadmehr and Krakauer, 2008). While our findings could be explained using step length asymmetry (which is a movement error) as the sole error signal, we know from previous work that this is not the case. Indeed, recalibration in walking adaptation can proceed, at least in part, when the movement error is clamped (Long et al., 2016). More specifically, part of the recalibration can be affected by clamping manipulations, and some cannot (Gonzalez-Rubio et al., 2019). We therefore speculate that recalibration may be driven by a combination of movement errors - step length asymmetry and other kinematics and kinetics - and sensory prediction errors – related to leg speed difference but also kinematic and kinetic predictions.

The idea that forward model recalibration is composite of multiple signals is supported by animal work showing that different signals may be combined at different stages of the cerebellar computation. For example, work in the electric fish suggests the same Purkinje cell may implement cancellation of different sensory signals (Lai et al., 2021). Signals may also be combined in the cerebellar nuclei, which receives convergent input from multiple Purkinje cells (Ito, 1984; Tanaka et al., 2020). Finally, recalibration of different movement parameters may travel via cerebellar projections to different cortical premotor neurons (Herzfeld et al., 2018), and be combined at cortical level to affect the overall movement. Indeed, cerebellar circuits are anatomically homogeneous, and it is thought that they may perform analogous computations and still have differing functional roles depending on their inputs and downstream output structures (Ito, 1984; Schmahmann, 1996; Tanaka et al., 2020).

Similarly, individual error signals may either drive learning in respective recalibration submodules in isolation, or may first be combined to produce a composite error signal (Diedrichsen et al.,

2019; Tanaka et al., 2020). Indeed, individual climbing fibers in the inferior olive have been shown to relay composite, multisensory information to Purkinje cells (Ju et al., 2019).

(9) Mapping may underly savings upon re-exposure to the same or different perturbation

We suggest that mapping may underly the phenomenon of savings – the phenomenon of smaller initial errors, faster relearning, and smaller aftereffects when re-adapting to the same perturbation (Day et al., 2018; Krakauer and Shadmehr, 2006; Kristan A. Leech et al., 2018; Malone et al., 2011; Martin et al., 1996; Reisman et al., 2013; Roemmich and Bastian, 2015; Rossi et al., 2021b; Shadmehr and Brashers-Krug, 1997). Mapping is ideal for savings because, in contrast to recalibration, it can take a value of zero during post-adaptation without having to unlearn. This means that, upon readaptation, the mapping adjustment may be able to immediately "jump" to a positive value, rather than adapting de novo from zero like recalibration. This may give mapping a competitive advantage such that, with repeated exposure to split-belt walking, it may contribute relatively more than recalibration to the overall adaptation. Indeed, the recall of having experienced a larger perturbation in adaptation is associated with larger savings (Roemmich and Bastian, 2015); this supports the involvement of mapping to savings because mapping adjustments scale with the perceived perturbation. Furthermore, a similar observation was found for reaching adaptation, where savings has opposite effects on recalibration and explicit control (Avraham et al., 2021). Finally, the idea that savings relates to the mapping mechanism is supported by the fact that learning is facilitated when re-exposed not only to the same perturbation, but also to perturbations of different magnitudes (Bond and Taylor, 2017; Kristan A Leech et al., 2018).

We proposed that, after repeated adaptation exposures, mapping reduces the magnitude of motor aftereffects not only by "taking over" most of the adaptation (such that recalibration is less), but also by actively counteracting recalibration-related motor aftereffects during post-adaptation. This framework helps explain findings from Leech et al. that motor and perceptual aftereffects decay differently over multiple days of adaptation (Kristan A. Leech et al., 2018). The study showed that after 4 or 5 days of learning, participants still have perceptual but no motor aftereffects. We suggest that this is not indicative of different motor and perceptual recalibrations. Rather, recalibration may still be producing *positive* adjustments to both movement and perception. The mapping mechanism may have learnt to produce *negative* adjustments to the motor output such

that the overall motor output is zero and there are no visible motor aftereffects; mapping cannot affect perception such that perceptual aftereffects are still present.

The idea that a learning mechanism of locomotor adaptation may learn *in the opposite direction* than the experienced perturbation is not new: previous work has demonstrated savings following adaptation to the opposite split-belt perturbation (Malone et al., 2011). Mapping may be learning *negative* motor adjustments during post-adaptation because, as discussed in the main text, it depends on the *perceived* belt speed difference, rather than the actual. During post-adaptation, people *perceive* the opposite belt speed difference (right leg feels *slower* than the left on tied belts), and this exposure may drive learning of a mapping that corrects for the perceived *negative* perturbations. This learning may accumulate with repeated days of adaptation until mapping can fully counteract recalibration adjustments to the motor output.

Finally, together with the fact that mapping contributes to adaptation but not to the aftereffects, the idea that mapping can counteract recalibration-related aftereffects may explain why motor and perceptual measures are not always found to be related (Rossi et al., 2019; Statton et al., 2018). We suggest that the lack correlation between measures of adaptation (e.g. learning rate) and perceptual aftereffects may be due to the fact that different participants can adapt similar amounts and at a similar rate, while engaging recalibration versus mapping in different proportions. The lack of correlation between motor and perceptual aftereffects may be due to mapping counteracting recalibration-related aftereffects to different extents in different participants.

(10) Implications for models of adaptation

The properties of adaptation described here appear at odds with prominent computational frameworks of adaptation, and offer insights into how models may incorporate the operation of fundamentally different adaptive mechanisms. In line with previous suggestions (Taylor and Ivry, 2012), our results highlight the importance of accounting for adaptation mechanisms able to produce flexible motor adjustments. In particular, as shown for the dual state model (see Fig. 3; (Smith et al., 2006)), state space models (e.g. (Coltman et al., 2019; Diedrichsen et al., 2010; Inoue et al., 2015; Lee and Schweighofer, 2009; Roemmich et al., 2016; Tanaka et al., 2012)) may generally not capture the behavior of the mapping-like mechanisms: the states can only store a fixed motor adjustment at each given time, and may therefore only capture recalibration-like mechanisms. A model from Taylor and Ivry (Taylor and Ivry, 2011) incorporates strategic aiming

mechanisms seen in reaching adaptation into state space models in a simple and elegant way, and is able to capture the flexible motor adjustments seen in our Ramp Down. However, the model assumes that mapping is immediately available upon exposure to the adaptation perturbation (as is the case when an aiming strategy is given to the participant). It may not capture uninstructed aiming strategies – which are fast but not immediate (Bond and Taylor, 2015; McDougle et al., 2015; Taylor et al., 2014) - nor the mapping we see in walking adaptation – which learns at a similar rate to recalibration.

Optimal control theories combining state estimation with feedback control (e.g. (Izawa and Shadmehr, 2011; Shadmehr and Krakauer, 2008; Todorov, 2005, 2004)) offer an interesting perspective on how mapping-like mechanisms may be incorporated. In these frameworks, sensory estimates by a forward model are passed to a controller, which then computes the "optimal" motor command. Of note, these computations are respectively attributed to cerebellum + parietal lobe, and motor cortical areas (Shadmehr and Krakauer, 2008), in line with what discussed in a previous section. Optimal control appears to have the potential to account for mapping-like mechanisms seen in both walking and reaching, because the definition of "optimal" accounts for both implicit and explicit rewards and costs (Todorov, 2004; Todorov and Jordan, 2002). Mapping in walking adaptation may be explained by the optimization of implicit step length asymmetry costs, as suggested by our data, perhaps combined with energetics or other signals suggested in other work (e.g. (Finley et al., 2013; Sánchez et al., 2019, 2017)). Despite their potential, typical implementations of optimal control theory for adaptation do not account for the architecture uncovered in our study. This is because the feedback controller is typically assumed to only use information from the forward model state estimate (i.e. x_r in Fig. 7), without access to actual sensory information from the environment (i.e., without $\tilde{p}$ nor p as inputs) (Izawa and Shadmehr, 2011; Shadmehr and Krakauer, 2008; Todorov, 2005). The state estimate can only represent the fixed adjustment by the recalibration mechanism, because it is updated trial-by-trial in small increments (i.e. learning rate or Kalman gains typically much smaller than 1). This indicates that, in traditional implementations, the feedback controller does not have direct nor rapid access to environmental changes. If the input to the controller is fixed and does not scale with belt speed difference, then motor adjustments cannot flexibly scale with the perturbation like seen for the mapping mechanism (see computation in dark blue box, Fig. 7). While there have been some suggestions of controllers with access to both internal state estimates and sensory

feedback (e.g. (Cheng and Sabes, 2006; Honda et al., 2018)), a specific model accounting for mapping-like observations regardless of explicit control remains to be determined.

Another interesting implication of our findings is that the recalibration and mapping mechanisms are arranged in “tandem”, with intermediate features between parallel and serial arrangements (Honda et al., 2018): like serial arrangements, the recalibration is computed first, and it serves as an input to the mapping mechanism. Like parallel arrangements, the recalibration and mapping mechanisms learn from external errors rather than from each other. The serial-like arrangement is implied by the observation that the input to mapping is the *perceived* perturbation ($\tilde{p}$), and the parallel-like arrangement by the fact that mapping competes in part to correct the same error as recalibration (see previous Discussion sections). The tandem architecture is at odds with work from Lee & Schweighofer, who suggests that adaptation mechanisms are arranged in parallel with not access to each other’s output (Lee and Schweighofer, 2009). We suggest this may be because the study only tested an alternative serial architecture, where one mechanism learns from the output of the other with no access to environmental information. Future work should determine whether the tandem architecture observed here may also be better suited to capture adaptation of reaching and other movements.

Pseudocode

Pseudocode for the clustering analysis.

```
Notation (A x B) indicates matrix size. N = 20, K = 20 (i.e. the number of participants)

a.  Initialize BestEpsilon (1 x K), BestMinPts (1 x K), BestNumberOutliers (1 x K), BestNumberClusters (1 x K)

b.  Repeats steps 1-12 for k=1:K:
    1.  Dst (N x N) = distance of between each pair of participants:
        for n1=1:N
            for n2=1:N
                Dst (n1,n2) = abs (measure(n1) – measure(n2) )
    2.  AvgDst (N x 1) = mean distance to the first 'k' neighbors (for each participant):
        for n=1:N
            AvgDst(n) = mean ( Dst(n,1:k) )
    3.  SortAvgDst (N x 1) = AvgDst sorted in ascending order
    4.  SortAvgDstD (N-1 x 1) = first derivative of SortAvgDst
    5.  SortAvgDstDD (N-2 x 1) = second derivative of SortAvgDst
    6.  iEpsilon (M x 1) = indexes i where SortAvgDstDD (i) > 1/100 * mean(SortAvgDstD)
        (size M is automatic based on step 6)
    7.  Epsilon (M x 1) = SortAvgDst(iEpsilon+1)
    8.  SortEpsilon (M x 1) = Epsilon (j) where SortAvgDstDD (j) is sorted in descending order (i.e. sort Epsilon
        by descending SortAvgDstDD values)
    9.  MinPts (M x 1):
        for m=1:M
            initialize NumberNeighbors (N x 1)
            for n=1:N
                NumberNeighbors(n) = number of elements for which Dst (n, : ) ≤ SortEpsilon(m)
            MinPts(m) = mean (NumberNeighbors)
    10. Eliminate SortEpsilon (j) and MinPts (j) for which MinPts (j) < 2 or MinPts (j) > 10
    11. NumberOutliers (Q x 1): (Q is the size of SortEpsilon and MinPts after step 10)
        for q=1:Q
            clusters = dbscan (measure, SortEpsilon (q), MinPts(q) )
            NumberOutliers(q) = number of participants not assigned to any clusters
                (e.g. NumberOutliers(q) = number of elements for which clusters == -1,
                 with -1 = the label for outliers)
            NumberClusters(q) = number of different clusters
                (e.g. NumberCluster(q) = number of unique clusters different than -1,
                 with -1 = the label for outliers)
    12. BestEpsilon(k), BestMinPts(k), BestNumberOutliers(k), BestNumberClusters(k) = SortEpsilon(q),
        MinPts(q), NumberOutliers(q), NumberClusters(q), where q is the smallest value for which
        NumberOutliers(q) == min(NumberOutliers)

c.  OutlierBestEpsilon (J x 1), OutlierBestMinPts (J x 1), OutlierBestNumberClusters (J x 1) = BestEpsilon(j),
    BestMinPts(j), BestNumberClusters(j) for all j such that BestNumberOutliers(j) ==
    min(BestNumberOutliers) (size J is automatic based on step 12)

d.  OverallBestEspilon (1 x 1), OverallBestMinPts (1 x 1) = OutlierBestEpsilon(j), OutlierBestMinPts(j) for j
    such that OutlierBestNumberClusters(j) == min(OutlierBestNumberClusters)
    (note that in our dataset, we could choose any j in the last step and the resulting clustering is the same.
    This may not be true for different datasets)
```

Supplementary Tables

Table S1. Experiment 1, CI of step length asymmetry for each speed in the Ramp Up and Ramp Down tasks. 95% bootstrapped confidence interval was corrected for multiple comparisons using false discovery rate (Benjamini and Yekutieli, 2005), Ramp Up $\boldsymbol{\alpha}_{\text{corrected}} = \frac{\textbf{6 significant comparisons}}{\textbf{7 total comparisons}} * \mathbf{0.05} = \mathbf{0.0429}$, Ramp Down $\boldsymbol{\alpha}_{\text{corrected}} = \frac{\textbf{12 significant comparisons}}{\textbf{21 total comparisons}} * \mathbf{0.05} = \mathbf{0.0286}$. Corrected CIs significantly different from zero are highlighted. Left speed was constant at 0.5m/s.

	right speed (m/s)	mean	95% CI	Corrected CI
Baseline Ramp Up	0.35	0.203	[0.177, 0.231]	**{0.175, 0.232}**
	0.4	0.078	[0.049, 0.105]	**{0.048, 0.106}**
	0.45	0.006	[-0.017, 0.028]	-
	0.5	-0.042	[-0.064, -0.020]	**{-0.065, -0.019}**
	0.55	-0.088	[-0.126, -0.050]	**{-0.127, -0.049}**
	0.6	-0.123	[-0.149, -0.098]	**{-0.150, -0.097}**
	0.65	-0.144	[-0.171, -0.119]	**{-0.172, -0.118}**
Post-adaptation Ramp Down	1.5	-0.015	[-0.038, 0.009]	-
	1.45	-0.011	[-0.035, 0.014]	-
	1.4	-0.01	[-0.030, 0.010]	-
	1.35	0.002	[-0.025, 0.029]	-
	1.3	0	[-0.027, 0.031]	-
	1.25	0.015	[-0.013, 0.045]	-
	1.2	0.018	[-0.004, 0.042]	-
	1.15	0.005	[-0.019, 0.029]	-
	1.1	0.024	[0.000, 0.052]	{-0.002, 0.056}
	1.05	0.023	[0.002, 0.044]	{-0.001, 0.047}
	1	0.026	[-0.001, 0.052]	-
	0.95	0.038	[0.014, 0.059]	**{0.011, 0.062}**
	0.9	0.056	[0.026, 0.083]	**{0.023, 0.087}**
	0.85	0.067	[0.036, 0.097]	**{0.032, 0.100}**
	0.8	0.083	[0.056, 0.109]	**{0.053, 0.113}**
	0.75	0.112	[0.084, 0.138]	**{0.081, 0.142}**
	0.7	0.126	[0.091, 0.160]	**{0.087, 0.164}**
	0.65	0.154	[0.117, 0.192]	**{0.113, 0.196}**
	0.6	0.174	[0.143, 0.206]	**{0.139, 0.209}**
	0.55	0.204	[0.166, 0.243]	**{0.162, 0.248}**
	0.5	0.213	[0.172, 0.254]	**{0.168, 0.259}**

Supplementary Tables

Table S2. Control experiments, CI of step length asymmetry in the first and last stride of the speed match task (first post-adaptation task). 95% bootstrapped confidence interval was corrected for multiple comparisons using false discovery rate (Benjamini and Yekutieli, 2005), first stride $\boldsymbol{\alpha}_{\mathbf{corrected}} = \frac{\mathbf{5\ significant\ comparisons}}{\mathbf{7\ total\ comparisons}} * \mathbf{0.05} = \mathbf{0.0357}$, last stride $\boldsymbol{\alpha}_{\mathbf{corrected}} = \frac{\mathbf{2\ significant\ comparisons}}{\mathbf{7\ total\ comparisons}} * \mathbf{0.05} = \mathbf{0.0143}$. Corrected CIs significantly different from zero are highlighted.

	Group	mean	95% CI	Corrected CI
**First stride**	ShortAscend	0.4572	[0.3554, 0.5612]	**{0.3474, 0.5685}**
	MediumAscend	0.433	[0.2786, 0.5999]	**{0.2712, 0.6125}**
	LongAscend	0.3887	[0.1106, 0.6414]	**{0.0909, 0.6569}**
	ShortDescend	-0.1385	[-0.1846, -0.1001]	**{-0.1880, -0.0972}**
	MediumDescend	-0.0165	[-0.0691, 0.0393]	-
	SmallAbrupt	-0.0308	[-0.0693, 0.0030]	-
	SmallGradual	-0.0648	[-0.1210, -0.0063]	**{-0.1240, -0.0023}**
**Last stride**	ShortAscend	-0.014	[-0.0291, 0.0007]	-
	MediumAscend	0.0021	[-0.0396, 0.0445]	-
	LongAscend	0.0224	[0.0003, 0.0471]	{-0.0045, 0.0542}
	ShortDescend	-0.0352	[-0.0695, -0.0025]	{-0.0778, 0.0052}
	MediumDescend	0.0431	[-0.0004, 0.0899]	-
	SmallAbrupt	0.0306	[-0.0005, 0.0617]	-
	SmallGradual	-0.0041	[-0.0323, 0.0246]	-

Supplementary Tables

Table S3. Control experiments, within-group difference between $\boldsymbol{compensation}_{\text{motor total}}$ and $\boldsymbol{compensation}_{\text{perceptual}}$. 95% bootstrapped confidence interval was corrected for multiple comparisons using false discovery rate (Benjamini and Yekutieli, 2005), $\boldsymbol{\alpha}_{\text{corrected}} = \frac{\text{7 significant comparisons}}{\text{7 total comparisons}} * \mathbf{0.05} = \mathbf{0.05}$. Significant differences based on corrected CI (Efron and Tibshirani, 1994) are highlighted.

Group	Mean	95% CI	Corrected CI
Short Ascend	47.450	[36.082, 59.494]	**{36.082, 59.494}**
Medium Ascend	48.295	[38.795, 57.273]	**{38.795, 57.273}**
Long Ascend	55.565	[44.325, 66.551]	**{44.325, 66.551}**
Short Descend	36.252	[27.434, 44.096]	**{27.434, 44.096}**
Medium Descend	56.012	[42.462, 69.950]	**{42.462, 69.950}**
Small Abrupt	36.224	[17.254, 55.718]	**{17.254, 55.718}**
Small Gradual	15.824	[3.975, 27.015]	**{3.975, 27.015}**

Supplementary Tables

Table S4. Control experiments, within-group comparison of $\boldsymbol{compensation}_{\textbf{motor total}}$ to 100%. 95% bootstrapped confidence interval was corrected for multiple comparisons using false discovery rate (Benjamini and Yekutieli, 2005), $\alpha_{corrected} = \frac{5\ significant\ comparisons}{7\ total\ comparisons} * 0.05 = 0.0357$. Corrected CIs significantly different from 100% are highlighted.

Group	Mean	95% CI	Corrected CI
ShortAscend	73.200	[65.330, 81.111]	**{64.812, 81.563}**
MediumAscend	90.045	[86.579, 93.965]	**{86.336, 94.239}**
LongAscend	98.815	[93.457, 104.583]	-
ShortDescend	70.752	[63.513, 77.437]	**{63.013, 77.843}**
MediumDescend	91.012	[84.132, 98.274]	**{83.758, 98.773}**
SmallAbrupt	98.349	[86.146, 111.269]	-
SmallGradual	73.074	[61.812, 85.213]	**{61.141, 85.994}**

Supplementary Tables

Table S5. Control experiments, between-group differences in $\boldsymbol{compensation}_{\textbf{motor total}}$. 95% bootstrapped confidence interval was corrected for multiple comparisons using false discovery rate (Benjamini and Yekutieli, 2005), $\alpha_{corrected} = \frac{3\ significant\ comparisons}{6\ total\ comparisons} * 0.05 = 0.025$. Significant differences based on corrected CI (Efron and Tibshirani, 1994) are highlighted.

Groups	Mean	95% CI	Corrected CI
MediumAscend - ShortAscend	16.845	[7.981, 25.580]	**{7.029, 26.780}**
LongAscend - MediumAscend	8.770	[2.197, 15.529]	**{1.126, 16.418}**
ShortDescend - ShortAscend	-2.448	[-13.194, 7.983]	-
MediumDescend - MediumAscend	0.967	[-6.939, 9.079]	-
SmallAbrupt - MediumDescend	7.336	[-6.881, 21.821]	-
SmallGradual - SmallAbrupt	-25.275	[-42.137, -7.837]	**{-44.829, -5.784}**

Supplementary Tables

Table S6. Control experiments, between-group differences in recalibration contribution to total output ($compensation_{\text{perceptual}}$ / $compensation_{\text{motor total}}$). 95% bootstrapped confidence interval was corrected for multiple comparisons using false discovery rate (Benjamini and Yekutieli, 2005), $\alpha_{corrected} = \frac{1\ significant\ comparison}{6\ total\ comparisons} * 0.05 = 0.0083$. Significant differences based on corrected CI (Efron and Tibshirani, 1994) are highlighted.

Groups	Mean	95% CI	Corrected CI
Medium Ascend – Short Ascend	9.277	[-5.880, 24.373]	-
Long Ascend – Medium Ascend	-2.049	[-17.108, 12.192]	-
Short Descend – Short Ascend	12.343	[-3.167, 28.054]	-
Medium Descend – Medium Ascend	-7.303	[-24.239, 9.448]	-
Small Abrupt – Medium Descend	26.546	[7.235, 47.436]	**{0.488, 54.744}**
Small Gradual – Small Abrupt	16.238	[-5.608, 38.497]	-

Supplementary Tables

Table S7. Experiment 2, clustering analysis measures and results for individual participants. Left column: 95% confidence interval of each participant's baseline (second tied-belt block). Middle column: number of strides in the first portion of the ramp up and down (speeds larger than adaptation) with step length asymmetry below the participant's own baseline CI. Right column: clustering classification (cluster 1, memory; or cluster 2, structure).

Participant	BL mean [95% CI]	# strides < CI	Cluster
P1	0.027 [0.016, 0.039]	21	Cluster 2 (structure)
P2	0.009 [0.004, 0.015]	42	Cluster 1 (memory)
P3	-0.007 [-0.014, -0.000]	55	Cluster 1 (memory)
P4	-0.017 [-0.025, -0.008]	14	Cluster 2 (structure)
P5	0.094 [0.082, 0.107]	58	Cluster 1 (memory)
P6	0.072 [0.060, 0.084]	50	Cluster 1 (memory)
P7	0.004 [-0.002, 0.011]	21	Cluster 2 (structure)
P8	0.024 [0.015, 0.032]	51	Cluster 1 (memory)
P9	-0.045 [-0.053, -0.037]	44	Cluster 1 (memory)
P10	-0.020 [-0.028, -0.013]	3	Cluster 2 (structure)
P11	0.002 [-0.007, 0.010]	60	Cluster 1 (memory)
P12	-0.043 [-0.055, -0.032]	16	Cluster 2 (structure)
P13	-0.027 [-0.037, -0.016]	40	Cluster 1 (memory)
P14	0.007 [-0.009, 0.024]	58	Cluster 1 (memory)
P15	-0.055 [-0.068, -0.043]	4	Cluster 2 (structure)
P16	-0.008 [-0.018, 0.003]	40	Cluster 1 (memory)
P17	-0.068 [-0.081, -0.055]	16	Cluster 2 (structure)
P18	-0.034 [-0.042, -0.026]	11	Cluster 2 (structure)
P19	-0.009 [-0.017, -0.000]	56	Cluster 1 (memory)
P20	0.008 [-0.007, 0.022]	38	Cluster 1 (memory)

Supplementary Tables

Table S8. Experiment 1, clustering analysis measures and results for individual participants. Left column: 95% confidence interval of each participant's baseline (second tied-belt block). Middle column: number of strides in the ramp down with step length asymmetry above the participant's own baseline CI. Right column: clustering classification (cluster 1 or outlier).

Participant	BL mean [95% CI]	# strides > CI	Cluster
P1	0.013 [0.005, 0.021]	38	Cluster 1
P2	*-0.002 [-0.009, 0.006]*	*16*	*outlier*
P3	*0.106 [0.092, 0.121]*	*13*	*outlier*
P4	0.005 [-0.008, 0.018]	53	Cluster 1
P5	0.019 [0.007, 0.032]	47	Cluster 1
P6	-0.040 [-0.058, -0.024]	57	Cluster 1
P7	-0.009 [-0.019, 0.002]	58	Cluster 1
P8	0.027 [0.017, 0.037]	25	Cluster 1
P9	-0.019 [-0.030, -0.009]	37	Cluster 1
P10	-0.007 [-0.016, 0.003]	48	Cluster 1
P11	-0.038 [-0.045, -0.032]	29	Cluster 1
P12	-0.012 [-0.025, 0.000]	60	Cluster 1
P13	0.022 [0.013, 0.030]	40	Cluster 1
P14	-0.046 [-0.054, -0.039]	40	Cluster 1
P15	-0.001 [-0.010, 0.008]	33	Cluster 1
P16	-0.066 [-0.073, -0.059]	47	Cluster 1
P17	0.027 [0.016, 0.038]	59	Cluster 1
P18	0.011 [-0.001, 0.024]	38	Cluster 1
P19	-0.060 [-0.077, -0.043]	49	Cluster 1
P20	0.048 [0.039, 0.057]	30	Cluster 1

Supplementary Tables

Table S9. Experiment 1, replication of statistical analyses after removal of the 2 outliers detected with the primary clustering analysis. Measures are reported the same way as the original analysis (rows 1-7 report mean [95% CI] {corrected CI}; rows 8-9 report correlation coefficient and p value). Significant results are highlighted. Removing the outliers did not affect the statistical significance of any of the tests. UB = upper bound, LB = lower bound.

Measure	Statistical Results
"Recalibration + Mapping" – "Recalibration Only" BIC	34.046 [21.715, 47.012] **{21.715, 47.012}**
"Recalibration + Mapping" – "Mapping Only" BIC	59.509 [43.682, 76.348] **{43.682, 76.348}**
"Recalibration + Mapping" – "Dual State" BIC	8.471 [3.323, 13.934] **{3.323, 13.934}**
$compensation_{\text{motor total}} - compensation_{\text{perceptual UB}}$	30.574 [23.788, 37.855] **{23.788, 37.855}**
$compensation_{\text{motor total}} - compensation_{\text{perceptual LB}}$	58.074 [49.016, 67.602] **{49.016, 67.602}**
$compensation_{\text{motor recalibration}} - compensation_{\text{perceptual UB}}$	-7.606 [-14.425, -1.198] **{-14.425, -1.198}**
$compensation_{\text{motor recalibration}} - compensation_{\text{perceptual LB}}$	19.894 [9.259, 30.553] **{9.259, 30.553}**
Correlation between $compensation_{\text{motor recalibration}}$ & $compensation_{\text{perceptual UB}}$	r=0.59, **p=0.010**
Correlation between $compensation_{\text{motor recalibration}}$ & $compensation_{\text{perceptual LB}}$	r=0.30, p=0.226

Supplementary Tables

Table S10. Experiment 2, responses to questionnaire. Raw responses of individual participants (center column) sorted by subgroup (left column), and classification of responses (three rightmost columns) used for Fig. 6.

		Response to:<br>Did you deliberately change how you walked to account for how fast the belts were moving? If so, describe how.<br>*Note: deliberately means that you thought about and decided to move that way*	**deliberate?**	**relevant?**	**accurate?**
**Memory-Based**	P2	I noticed that I was making heavy steps on the right foot which caused shifting in my upper body so I tried to control that a little bit, but not much	Y	N	-
	P3	Yes, I tried to adjust how fast I changed foot while walking, and tried to shift more weight onto my left side which was walking at a slower pace.	Y	Y	N
	P5	Yes, I need to change how I walk because the belt moves in different speed	Y	N	-
	P6	Yes, I changed my pace to stay upright and not fall down	Y	N	-
	P8	No	N	-	-
	P9	Yes, I started to change how I walked when I realized the right belt was moving faster, and I had to compensate on my left to stabilize my body.	Y	N	-
	P11	No.	N	-	-
	P13	Yes. I tried to get into a rhythm so that I had consistently long right strides and short left strides.	Y	N	-
	P14	Yes, I tried to bow out my right leg(for the period of the time where the right belt was moving faster) so as to give me additional balance. I also tried to sync up my right foot moving up off the track at the same or close time as my left foot hit the track for the portion of the block where the right track was moving faster	Y	Y	N
	P20	Yes, as soon as I felt the right belt's speed increase, I tried to spend as much time leaning on my left leg as possible and wait for a little before switching to my right foot (and then spending less time on my right foot)	Y	Y	Y
**Structure-Based**	P1	Matched duration of standing on each foot by moving the right leg faster than the left leg and at a wider angle (range of motion).	Y	Y	N
	P4	YES, I put less pressure on my right leg so I wouldn't lose my balance as the belts were moving at different rates.	Y	N	-
	P7	Tried to even out the limp caused by the different leg speeds	Y	Y	N
	P10	Yes I deliberately put more weight on my left foot (on the belt that was slower) to provide more stability for my right foot that was moving faster.	Y	N	-
	P12	I felt like I had to adjust my speed to keep up with the way the belts were moving differently, I was thinking about how the right belt was moving faster but I didn't really feel like I had control over my own gait	N	-	-
	P15	Yes, I adjusted as if I was limping.	Y	Y	N

SI References

Abeele S, Bock O. 2001. Mechanisms for sensorimotor adaptation to rotated visual input. *Exp Brain Res* **139**:248–253. doi:10.1007/S002210100768

Al-Yahya E, Dawes H, Smith L, Dennis A, Howells K, Cockburn J. 2011. Cognitive motor interference while walking: A systematic review and meta-analysis. *Neurosci Biobehav Rev*. doi:10.1016/j.neubiorev.2010.08.008

Avraham G, Morehead JR, Kim HE, Ivry RB. 2021. Reexposure to a sensorimotor perturbation produces opposite effects on explicit and implicit learning processes. *PLoS Biol* **19**:e3001147. doi:10.1371/journal.pbio.3001147

Bastian AJ. 2008. Understanding sensorimotor adaptation and learning for rehabilitation. *Curr Opin Neurol* **21**:628–633. doi:10.1097/WCO.0b013e328315a293

Benjamini Y, Yekutieli D. 2005. False discovery rate-adjusted multiple confidence intervals for selected parameters. *J Am Stat Assoc* **100**:71–81. doi:10.1198/016214504000001907

Berniker M, Kording K. 2008. Estimating the sources of motor errors for adaptation and generalization. *Nat Neurosci* **11**:1454–1461. doi:10.1038/nn.2229

Bock O, Schneider S, Bloomberg J. 2001. Conditions for interference versus facilitation during sequential sensorimotor adaptation. *Exp Brain Res* **138**:359–365. doi:10.1007/s002210100704

Bond KM, Taylor JA. 2017. Structural learning in a visuomotor adaptation task is explicitly accessible. *eNeuro* **4**:e0122-17. doi:10.1523/ENEURO.0122-17.2017

Bond KM, Taylor JA. 2015. Flexible explicit but rigid implicit learning in a visuomotor adaptation task. *J Neurophysiol* **113**:3836–3849. doi:10.1152/jn.00009.2015

Braun DA, Aertsen A, Wolpert DM, Mehring C. 2009. Motor Task Variation Induces Structural Learning. *Curr Biol* **19**:352–7. doi:10.1016/j.cub.2009.01.036

Buurke TJW, Lamoth CJC, Vervoort D, Van Der Woude LHV, Otter R den. 2018. Adaptive control of dynamic balance in human gait on a split-belt treadmill. *J Exp Biol* **221**. doi:10.1242/jeb.174896

Camacho S, Dop M, de Graaf C, Stieger M. 2015. Just Noticeable Differences and Weber Fraction of Oral Thickness Perception of Model Beverages. *J Food Sci* **80**:S1583–S1588. doi:10.1111/1750-3841.12922

Castro LN, Monsen CB, Smith MA. 2011. The binding of learning to action in motor adaptation. *PLoS Comput Biol* **7**:e1002052. doi:10.1371/journal.pcbi.1002052

Chase SM, Kass RE, Schwartz AB. 2012. Behavioral and neural correlates of visuomotor adaptation observed through a brain-computer interface in primary motor cortex. *J Neurophysiol* **108**:624–644. doi:10.1152/jn.00371.2011

Cheng S, Sabes PN. 2006. Modeling Sensorimotor Learning with Linear Dynamical Systems. *Neural Comput* **18**:760–793. doi:10.1162/089976606775774651

Clark DJ. 2015. Automaticity of walking: Functional significance, mechanisms, measurement and rehabilitation strategies. *Front Hum Neurosci* **9**:246. doi:10.3389/fnhum.2015.00246

Coltman SK, Cashaback JGA, Gribble PL. 2019. Both fast and slow learning processes contribute to savings following sensorimotor adaptation. *J Neurophysiol* **121**:1575–1583. doi:10.1152/jn.00794.2018

Darter BJ, Labrecque BA, Perera RA. 2018. Dynamic stability during split-belt walking and the relationship with step length symmetry. *Gait Posture* **62**:86–91. doi:10.1016/j.gaitpost.2018.03.006

Dassonville P, Lewis SM, Zhu XH, Ugurbil K, Kim SG, Ashe J. 2001. The effect of stimulus-response compatibility on cortical motor activation. *Neuroimage* **13**:1–14. doi:10.1006/nimg.2000.0671

Day KA, Leech KA, Roemmich RT, Bastian AJ. 2018. Accelerating locomotor savings in learning: Compressing four training days to one. *J Neurophysiol* **119**:2100–2113. doi:10.1152/jn.00903.2017

Diedrichsen J, King M, Hernandez-Castillo C, Sereno M, Ivry RB. 2019. Universal Transform or Multiple Functionality? Understanding the Contribution of the Human Cerebellum across Task Domains. *Neuron* **102**:918–928. doi:10.1016/j.neuron.2019.04.021

Diedrichsen J, White O, Newman D, Lally N. 2010. Use-dependent and error-based learning of motor behaviors. *J Neurosci* **30**:5159–5166. doi:10.1523/JNEUROSCI.5406-09.2010

Efron B, Tibshirani RJ. 1994. An Introduction to the Bootstrap, An Introduction to the Bootstrap. doi:10.1201/9780429246593

Ester M, Kriegel H-P, Sander J, Xu X. 1996. A Density-Based Algorithm for Discovering Clusters in Large Spatial Databases with NoiseProceedings of the 2nd International Conference on Knowledge Discovery and Data Mining. pp. 226–231.

Fasano A, Plotnik M, Bove F, Berardelli A. 2012. The neurobiology of falls. *Neurol Sci* **33**:1215–1223.

doi:10.1007/s10072-012-1126-6

Finley JM, Bastian AJ, Gottschall JS. 2013. Learning to be economical: The energy cost of walking tracks motor adaptation. *J Physiol* **591**:1081–1095. doi:10.1113/jphysiol.2012.245506

Gescheider GA. 1997. The Classical Psychophysical MethodsPsychophysics: The Fundamentals. Mahwah, NJ, US: Lawrence Erlbaum Associates Publishers. pp. 45–72.

Goldman-Rakic PS. 1987. Motor control function of the prefrontal cortex. *Ciba Found Symp* **132**:187–200. doi:10.1002/9780470513545.ch12

Gonzalez-Rubio M, Velasquez NF, Torres-Oviedo G. 2019. Explicit control of step timing during split-belt walking reveals interdependent recalibration of movements in space and time. *Front Hum Neurosci* **13**:207. doi:10.3389/fnhum.2019.00207

Gregory DL, Sup FC, Choi JT. 2021. Contributions of spatial and temporal control of step length symmetry in the transfer of locomotor adaptation from a motorized to a non-motorized split-belt treadmill. *R Soc Open Sci* **8**:202084. doi:10.1098/rsos.202084

Haith AM, Krakauer JW. 2013. Model-based and model-free mechanisms of human motor learning. *Adv Exp Med Biol* **782**:1–21. doi:10.1007/978-1-4614-5465-6_1

Hamzey RJ, Kirk EM, Vasudevan EVLL. 2016. Gait speed influences aftereffect size following locomotor adaptation, but only in certain environments. *Exp Brain Res* **234**:1479–1490. doi:10.1007/S00221-015-4548-6

Hardy L, Mullen R, Martin N. 2001. Effect of task-relevant cues and state anxiety on motor performance. *Percept Mot Skills* **92**:943–946. doi:10.2466/pms.2001.92.3.943

Haruno M, Wolpert DM, Kawato M. 2001. MOSAIC Model for Sensorimotor Learning and Control. *Neural Comput* **13**:2201–2220. doi:10.1162/089976601750541778

Herzfeld DJ, Kojima Y, Soetedjo R, Shadmehr R. 2018. Encoding of error and learning to correct that error by the Purkinje cells of the cerebellum. *Nat Neurosci* **21**:736–743. doi:10.1038/s41593-018-0136-y

Herzfeld DJ, Vaswani PA, Marko MK, Shadmehr R. 2014. A memory of errors in sensorimotor learning. *Science (80-)* **345**:1349–1353. doi:10.1126/science.1253138

Hinton DC, Thiel A, Soucy JP, Bouyer L, Paquette C. 2019. Adjusting gait step-by-step: Brain activation during split-belt treadmill walking. *Neuroimage* **202**:116095. doi:10.1016/j.neuroimage.2019.116095

Hirata K, Kokubun T, Miyazawa T, Yokoyama H, Kubota K, Sonoo M, Hanawa H, Kanemura N. 2019. Contribution of Lower Limb Joint Movement in Adapting to Re-establish Step Length Symmetry During Split-Belt Treadmill Walking. *J Med Biol Eng* **39**:693–701. doi:10.1007/s40846-018-0456-0

Honda T, Nagao S, Hashimoto Y, Ishikawa K, Yokota T, Mizusawa H, Ito M. 2018. Tandem internal models execute motor learning in the cerebellum. *Proc Natl Acad Sci U S A* **115**:7428–7433. doi:10.1073/pnas.1716489115

Huberdeau DM, Krakauer JW, Haith AM. 2015. Dual-process decomposition in human sensorimotor adaptation. *Curr Opin Neurobiol* **33**:71–77. doi:10.1016/j.conb.2015.03.003

Hyndman D, Ashburn A. 2004. “Stops walking when talking” as a predictor of falls in people with stroke living in the community. *J Neurol Neurosurg Psychiatry* **75**:994–997. doi:10.1136/jnnp.2003.016014

Inoue M, Uchimura M, Karibe A, O’Shea J, Rossetti Y, Kitazawa S. 2015. Three timescales in prism adaptation. *J Neurophysiol* **113**:328–338. doi:10.1152/jn.00803.2013

Ito M. 1984. The cerebellum and neural control, Raven, New York. Raven Press. doi:10.1016/0090-3019(85)90320-9

Izawa J, Shadmehr R. 2011. Learning from sensory and reward prediction errors during motor adaptation. *PLoS Comput Biol* **7**:e1002012. doi:10.1371/journal.pcbi.1002012

Joiner WM, Ajayi O, Sing GC, Smith MA. 2011. Linear hypergeneralization of learned dynamics across movement speeds reveals anisotropic, gain-encoding primitives for motor adaptation. *J Neurophysiol* **105**:45–59. doi:10.1152/jn.00884.2009

Ju C, Bosman LWJ, Hoogland TM, Velauthapillai A, Murugesan P, Warnaar P, van Genderen RM, Negrello M, De Zeeuw CI. 2019. Neurons of the inferior olive respond to broad classes of sensory input while subject to homeostatic control. *J Physiol* **597**:2483–2514. doi:10.1113/JP277413

Kagerer FA, Contreras-Vidal JL, Stelmach GE. 1997. Adaptation to gradual as compared with sudden visuo-motor distortions. *Exp Brain Res* **115**:557–561. doi:10.1007/PL00005727

Kluzik JA, Diedrichsen J, Shadmehr R, Bastian AJ. 2008. Reach adaptation: What determines whether we learn an internal model of the tool or adapt the model of our arm? *J Neurophysiol* **100**:1455–1464. doi:10.1152/jn.90334.2008

SI References

Krakauer JW, Shadmehr R. 2006. Consolidation of motor memory. *Trends Neurosci* **29**:58–64. doi:10.1016/j.tins.2005.10.003

Lai NY, Bell JM, Bodznick D. 2021. Multiple behavior-specific cancellation signals contribute to suppressing predictable sensory reafference in a cerebellum-like structure. *J Exp Biol* **224**:240143. doi:10.1242/jeb.240143

Lauzière S, Miéville C, Betschart M, Duclos C, Aissaoui R, Nadeau S. 2014. Plantarflexion moment is a contributor to step length after-effect following walking on a split-belt treadmill in individuals with stroke and healthy individuals. *J Rehabil Med* **46**:849–857. doi:10.2340/16501977-1845

Lazar G, Van Laer J. 1968. Adaptation to displaced vision after experience with lesser displacements. *Percept Mot Skills* **26**:579–582. doi:10.2466/pms.1968.26.2.579

Lee JY, Schweighofer N. 2009. Dual adaptation supports a parallel architecture of motor memory. *J Neurosci* **29**:10396–10404. doi:10.1523/JNEUROSCI.1294-09.2009

Lee K, Oh Y, Izawa J, Schweighofer N. 2018. Sensory prediction errors, not performance errors, update memories in visuomotor adaptation. *Sci Rep* **8**:1–9. doi:10.1038/s41598-018-34598-y

Leech Kristan A., Day KA, Roemmich RT, Bastian AJ. 2018. Movement and perception recalibrate differently across multiple days of locomotor learning. *J Neurophysiol* **120**:2130–2137. doi:10.1152/jn.00355.2018.-Learning

Leech Kristan A, Roemmich RT, Bastian AJ. 2018. Creating flexible motor memories in human walking. *Sci Rep* **8**:94. doi:10.1038/s41598-017-18538-w

Long AW, Roemmich RT, Bastian AJ. 2016. Blocking trial-by-trial error correction does not interfere with motor learning in human walking. *J Neurophysiol* **115**:2341–2348. doi:10.1152/jn.00941.2015

Malone LA, Vasudevan EVL, Bastian AJ. 2011. Motor adaptation training for faster relearning. *J Neurosci* **31**:15136–15143. doi:10.1523/JNEUROSCI.1367-11.2011

Mariscal DM, Iturralde PA, Torres-Oviedo G. 2020. Altering attention to split-belt walking increases the generalization of motor memories across walking contexts. *J Neurophysiol* **123**:1838–1848. doi:10.1152/jn.00509.2019

Martin TA, Keating JG, Goodkin HP, Bastian AJ, Thach WT. 1996. Throwing while looking through prisms II. Specificity and storage of multiple gaze-throw calibrations. *Brain* **119**:1199–1211. doi:10.1093/brain/119.4.1183

Masters R, Maxwell J. 2008. The theory of reinvestment. *Int Rev Sport Exerc Psychol* **1**:160–183. doi:10.1080/17509840802287218

Masters RSW, Polman RCJ, Hammond N V. 1993. “Reinvestment”: A dimension of personality implicated in skill breakdown under pressure. *Pers Individ Dif* **14**:655–666. doi:10.1016/0191-8869(93)90113-H

McDougle SD, Bond KM, Taylor JA. 2015. Explicit and implicit processes constitute the fast and slow processes of sensorimotor learning. *J Neurosci* **35**:9568–9579. doi:10.1523/JNEUROSCI.5061-14.2015

McDougle SD, Ivry RB, Taylor JA. 2016. Taking Aim at the Cognitive Side of Learning in Sensorimotor Adaptation Tasks. *Trends Cogn Sci* **20**:535–544. doi:10.1016/j.tics.2016.05.002

McFadyen BJ, Gagné MÈ, Cossette I, Ouellet MC. 2017. Using dual task walking as an aid to assess executive dysfunction ecologically in neurological populations: A narrative review **27**:722–743. doi:10.1080/09602011.2015.1100125

Miller EK, Cohen JD. 2001. An integrative theory of prefrontal cortex function. *Annu Rev Neurosci* **24**:167–202. doi:10.1146/annurev.neuro.24.1.167

Modchalingam S, Vachon CM, ’t Hart BM, Henriques DYP. 2019. The effects of awareness of the perturbation during motor adaptation on hand localization. *PLoS One* **14**:e0220884. doi:10.17605/OSF.IO/MX5U2

Morton SM, Bastian AJ. 2004. Prism adaptation during walking generalizes to reaching and requires the cerebellum. *J Neurophysiol* **92**:2497–2509. doi:10.1152/jn.00129.2004

Morton SM, Lang CE, Bastian AJ. 2001. Inter- and intra-limb generalization of adaptation during catching. *Exp Brain Res* **141**:438–445. doi:10.1007/s002210100889

Naik Gaonkar M, Sawant K. 2013. AutoEpsDBSCAN : DBSCAN with Eps Automatic for Large Dataset. *Int J Adv Comput Theory Eng* **2**:2319–2526.

Neville K-MM, Cressman EK. 2018. The influence of awareness on explicit and implicit contributions to visuomotor adaptation over time. *Exp Brain Res* **236**:2047–2059. doi:10.1007/s00221-018-5282-7

Paul SS, Ada L, Canning CG. 2005. Automaticity of walking–implications for physiotherapy practice. *Phys Ther Rev* **10**:15–23. doi:10.1179/108331905X43463

SI References

Pinto Y, Otten M, Cohen MA, Wolfe JM, Horowitz TS. 2011. The boundary conditions for Bohr's law: When is reacting faster than acting? *Attention, Perception, Psychophys* **73**:613–620. doi:10.3758/s13414-010-0057-7

Poggio T, Bizzi E. 2004. Generalization in vision and motor control. *Nature* **431**:768–774. doi:10.1038/nature03014

Rahmah N, Sitanggang IS. 2016. Determination of Optimal Epsilon (Eps) Value on DBSCAN Algorithm to Clustering Data on Peatland Hotspots in SumatraIOP Conference Series: Earth and Environmental Science. Institute of Physics Publishing. p. 012012. doi:10.1088/1755-1315/31/1/012012

Reisman DS, Bastian AJ, Morton SM. 2010. Neurophysiologic and rehabilitation insights from the split-belt and other locomotor adaptation paradigms. *Phys Ther* **90**:187–195. doi:10.2522/ptj.20090073

Reisman DS, Block HJ, Bastian AJ. 2005. Interlimb coordination during locomotion: what can be adapted and stored? *J Neurophysiol* **94**:2403–2415. doi:10.1152/jn.00089.2005

Reisman DS, McLean H, Keller J, Danks KA, Bastian AJ. 2013. Repeated split-belt treadmill training improves poststroke step length asymmetry. *Neurorehabil Neural Repair* **27**:460–468. doi:10.1177/1545968312474118

Roemmich RT, Bastian AJ. 2015. Two ways to save a newly learned motor pattern. *J Neurophysiol* **113**:3519–3530. doi:10.1152/jn.00965.2014

Roemmich RT, Long AW, Bastian AJ, Roemmich RT, Long AW, Bastian AJ. 2016. Seeing the Errors You Feel Enhances Locomotor Performance but Not Learning. *Curr Biol* **26**:1–10. doi:10.1016/j.cub.2016.08.012

Rossi C, Bastian AJ, Therrien AS. 2021a. Mechanisms of proprioceptive realignment in human motor learning. *Curr Opin Physiol* **20**:186–197. doi:10.1016/j.cophys.2021.01.011

Rossi C, Chau CW, Leech KA, Statton MA, Gonzalez AJ, Bastian AJ. 2019. The capacity to learn new motor and perceptual calibrations develops concurrently in childhood. *Sci Rep* **9**:9322. doi:10.1038/s41598-019-45074-6

Rossi C, Roemmich RT, Schweighofer N, Bastian AJ, Leech KA. 2021b. Younger and Late Middle-Aged Adults Exhibit Different Patterns of Cognitive-Motor Interference During Locomotor Adaptation, With No Disruption of Savings. *Front Aging Neurosci* **13**:789. doi:10.3389/fnagi.2021.729284

Saijo N, Gomi H. 2010. Multiple motor learning strategies in visuomotor rotation. *PLoS One* **5**:e9399. doi:10.1371/journal.pone.0009399

Sánchez N, Park S, Finley JM. 2017. Evidence of Energetic Optimization during Adaptation Differs for Metabolic, Mechanical, and Perceptual Estimates of Energetic Cost OPEN. *Sci Rep* **7**:7682. doi:10.1038/s41598-017-08147-y

Sánchez N, Simha SN, Donelan JM, Finley JM. 2021. Using asymmetry to your advantage: Learning to acquire and accept external assistance during prolonged split-belt walking. *J Neurophysiol* **125**:344–357. doi:10.1152/jn.00416.2020

Sánchez N, Simha SN, Donelan JM, Finley JM. 2019. Taking advantage of external mechanical work to reduce metabolic cost: the mechanics and energetics of split-belt treadmill walking. *J Physiol* **597**:4053–4068. doi:10.1113/JP277725

Schmahmann JD. 1996. From movement to thought: Anatomic substrates of the cerebellar contribution to cognitive processing. *Hum Brain Mapp* **4**:174–198. doi:10.1002/(SICI)1097-0193(1996)4:3<174::AID-HBM3>3.0.CO;2-0

Schneider W, Chein JM. 2003. Controlled & automatic processing: Behavior, theory, and biological mechanisms. *Cogn Sci* **27**:525–559. doi:10.1016/S0364-0213(03)00011-9

Seidler RD. 2004. Multiple Motor Learning Experiences Enhance Motor Adaptability. *J Cogn Neurosci* **16**:65–73. doi:10.1162/089892904322755566

Shadmehr R, Brashers-Krug T. 1997. Functional stages in the formation of human long-term motor memory. *J Neurosci* **17**:409–419. doi:10.1523/jneurosci.17-01-00409.1997

Shadmehr R, Krakauer JW. 2008. A computational neuroanatomy for motor control. *Exp Brain Res* **185**:359–381. doi:10.1007/S00221-008-1280-5/FIGURES/9

Smith MA, Ghazizadeh A, Shadmehr R. 2006. Interacting adaptive processes with different timescales underlie short-term motor learning. *PLoS Biol* **4**:e179. doi:10.1371/journal.pbio.0040179

Statton MA, Vazquez A, Morton SM, Vasudevan EVL, Bastian AJ. 2018. Making sense of cerebellar contributions to perceptual and motor adaptation. *Cerebellum* **17**:111–121. doi:10.1007/s12311-017-0879-0

Sun X, O'Shea DJ, Golub MD, Trautmann EM, Vyas S, Ryu SI, Shenoy K V. 2022. Cortical preparatory

activity indexes learned motor memories. *Nature* **602**:274–279. doi:10.1038/s41586-021-04329-x

Tanaka H, Ishikawa T, Lee J, Kakei S. 2020. The Cerebro-Cerebellum as a Locus of Forward Model: A Review. *Front Syst Neurosci* **14**:19. doi:10.3389/FNSYS.2020.00019/BIBTEX

Tanaka H, Krakauer JW, Sejnowski TJ. 2012. Generalization and multirate models of motor adaptation. *Neural Comput* **24**:939–966. doi:10.1162/NECO_a_00262

Taylor JA, Ivry RB. 2012. The role of strategies in motor learning. *Ann N Y Acad Sci* **1251**:1–12. doi:10.1111/j.1749-6632.2011.06430.x

Taylor JA, Ivry RB. 2011. Flexible cognitive strategies during motor learning. *PLoS Comput Biol* **7**:e1001096. doi:10.1371/journal.pcbi.1001096

Taylor JA, Krakauer JW, Ivry RB. 2014. Explicit and implicit contributions to learning in a sensorimotor adaptation task. *J Neurosci* **34**:3023–3032. doi:10.1523/JNEUROSCI.3619-13.2014

Thoroughman KA, Shadmehr R. 2000. Learning of action through adaptive combination of motor primitives. *Nature* **407**:742–747. doi:10.1038/35037588

Todorov E. 2005. Stochastic Optimal Control and Estimation Methods Adapted to the Noise Characteristics of the Sensorimotor System. *Neural Comput* **17**:1084–108.

Todorov E. 2004. Optimality principles in sensorimotor control. *Nat Neurosci*. doi:10.1038/nn1309

Todorov E, Jordan MI. 2002. Optimal feedback control as a theory of motor coordination. *Nat Neurosci* **5**:1226–1235. doi:10.1038/nn963

Torres-Oviedo G, Bastian AJ. 2010. Seeing is believing: Effects of visual contextual cues on learning and transfer of locomotor adaptation. *J Neurosci* **30**:17015–17022. doi:10.1523/JNEUROSCI.4205-10.2010

Tseng YW, Diedrichsen J, Krakauer JW, Shadmehr R, Bastian AJ. 2007. Sensory prediction errors drive cerebellum-dependent adaptation of reaching. *J Neurophysiol* **98**:54–62. doi:10.1152/jn.00266.2007

Turnham EJA, Braun DA, Wolpert DM. 2012. Facilitation of learning induced by both random and gradual visuomotor task variation. *J Neurophysiol* **107**:1111–1122. doi:10.1152/jn.00635.2011

Uiga L, Capio CM, Ryu D, Young WR, Wilson MR, Wong TWL, Tse ACY, Masters RSW. 2020. The Role of Movement-Specific Reinvestment in Visuomotor Control of Walking by Older Adults. *Journals Gerontol - Ser B Psychol Sci Soc Sci* **75**:282–292. doi:10.1093/geronb/gby078

Van Vugt FT, Ostry DJ. 2018. The structure and acquisition of sensorimotor maps. *J Cogn Neurosci* **30**:290–306. doi:10.1162/jocn_a_01204

Vazquez A, Statton MA, Busgang SA, Bastian AJ. 2015. Split-belt walking adaptation recalibrates sensorimotor estimates of leg speed but not position or force. *J Neurophysiol* **114**:3255–3267. doi:10.1152/jn.00302.2015

Wakatsuki T, Yamada N. 2020. Difference Between Intentional and Reactive Movement in Side-Steps: Patterns of Temporal Structure and Force Exertion. *Front Psychol* **11**:2186. doi:10.3389/fpsyg.2020.02186

Wan CY, Huon GF. 2005. Performance degradation under pressure in music: an examination of attentional processes. *Psychol Music* **33**:155–172. doi:10.1177/0305735605050649

Wang TY, Bhatt T, Yang F, Pai YC. 2011. Generalization of motor adaptation to repeated-slip perturbation across tasks. *Neuroscience* **180**:85–95. doi:10.1016/j.neuroscience.2011.02.039

Welchman AE, Stanley J, Schomers MR, Miall RC, Bülthoff HH. 2010. The quick and the dead: When reaction beats intentionProceedings of the Royal Society B: Biological Sciences. The Royal Society. pp. 1667–1674. doi:10.1098/rspb.2009.2123

Wolpert DM, Ghahramani Z, Flanagan JR. 2001. Perspectives and problems in motor learning. *Trends Cogn Sci*. doi:10.1016/S1364-6613(00)01773-3

Wong WL, Masters RSW, Maxwell JP, Abernethy AB. 2008. Reinvestment and falls in community-dwelling older adults. *Neurorehabil Neural Repair* **22**:410–414. doi:10.1177/1545968307313510

Wong WL, Masters RSW, Maxwell JP, Abernethy B. 2009. The role of reinvestment in walking and falling in community-dwelling older adults: Letters to the editor. *J Am Geriatr Soc*. doi:10.1111/j.1532-5415.2009.02228.x

Young WR, Ellmers TJ, Kinrade NP, Cossar J, Cocks AJ. 2020. Re-evaluating the measurement and influence of conscious movement processing on gait performance in older adults: Development of the Gait-Specific Attentional Profile. *Gait Posture* **81**:73–77. doi:10.1016/J.GAITPOST.2020.07.008

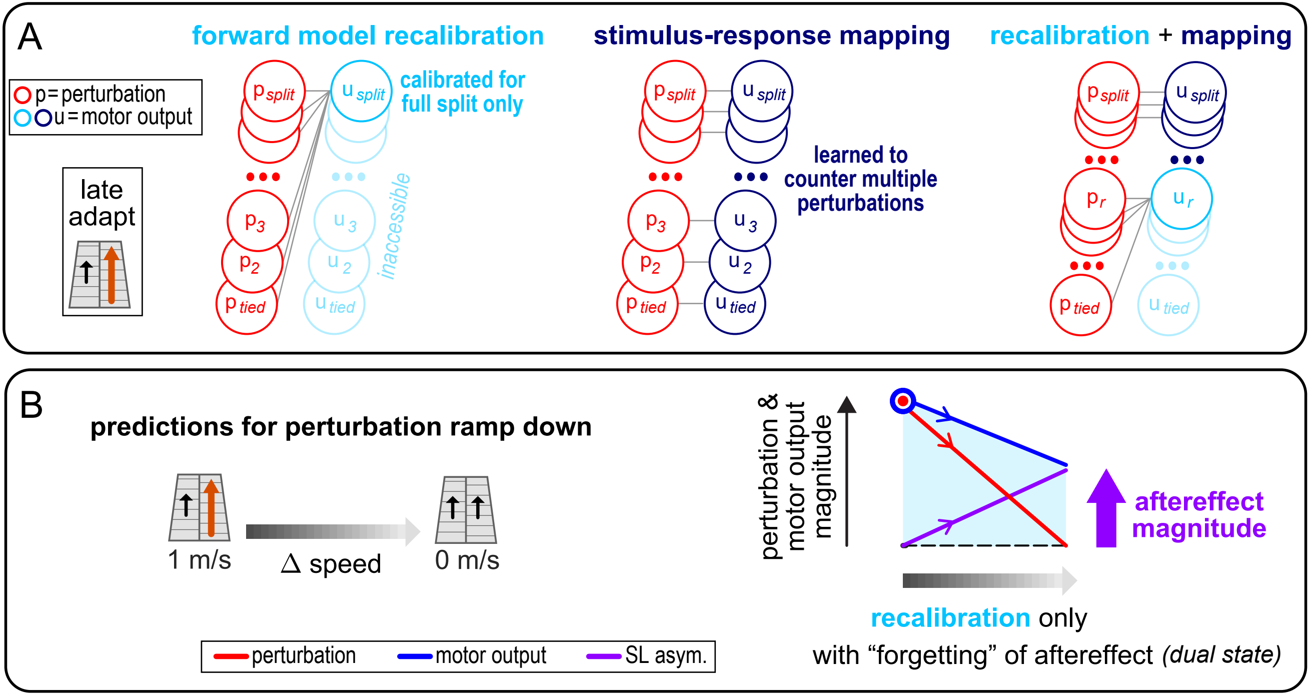

Figure S1. Experiment 1, mechanisms and additional predictions. (A) Illustration of how recalibration and mapping operate. Left: By the end of adaptation, recalibration can only access the last learnt motor output (u_{split}) and uses it regardless of the perturbation. Center: Mapping can access all motor outputs learnt in adaptation (dark blue) and can select the one matching the perturbation (red). Right: Mapping counters the perturbation not accounted for by recalibration if both mechanisms are at play. **(B)** Models such as the dual state incorporate stride-to-stride forgetting of the motor output, such that adapting by recalibration only may also explain the smaller aftereffect magnitude on tied belts.

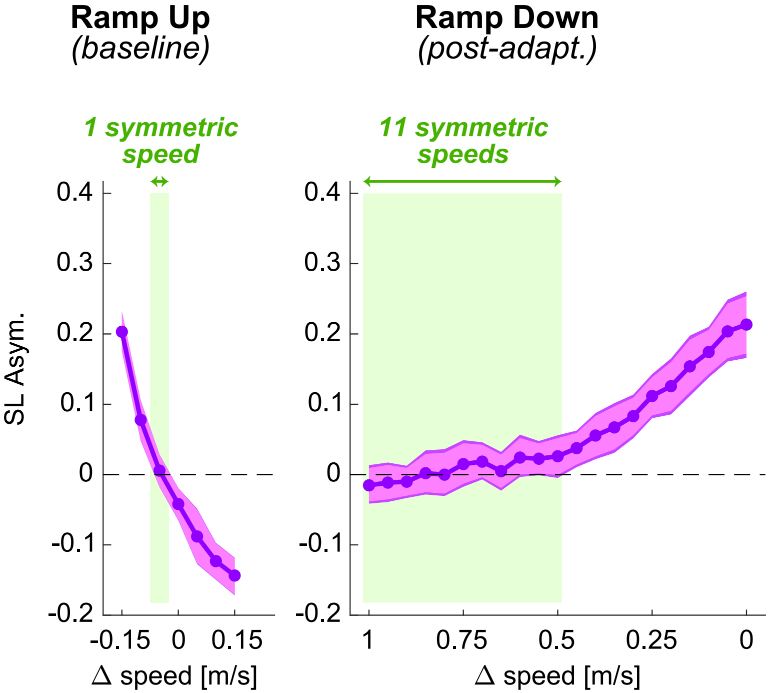

Figure S2. Experiment 1, step length asymmetry in ramp tasks. Step length asymmetry (pre-averaged within participant for strides taken at the same speed) as a function of speed (mean, purple curve and dots; 95% CI, light shade; and CI corrected for multiple comparisons, darker shade). Green shaded area represents speeds for which step length asymmetry is not significantly different than zero.

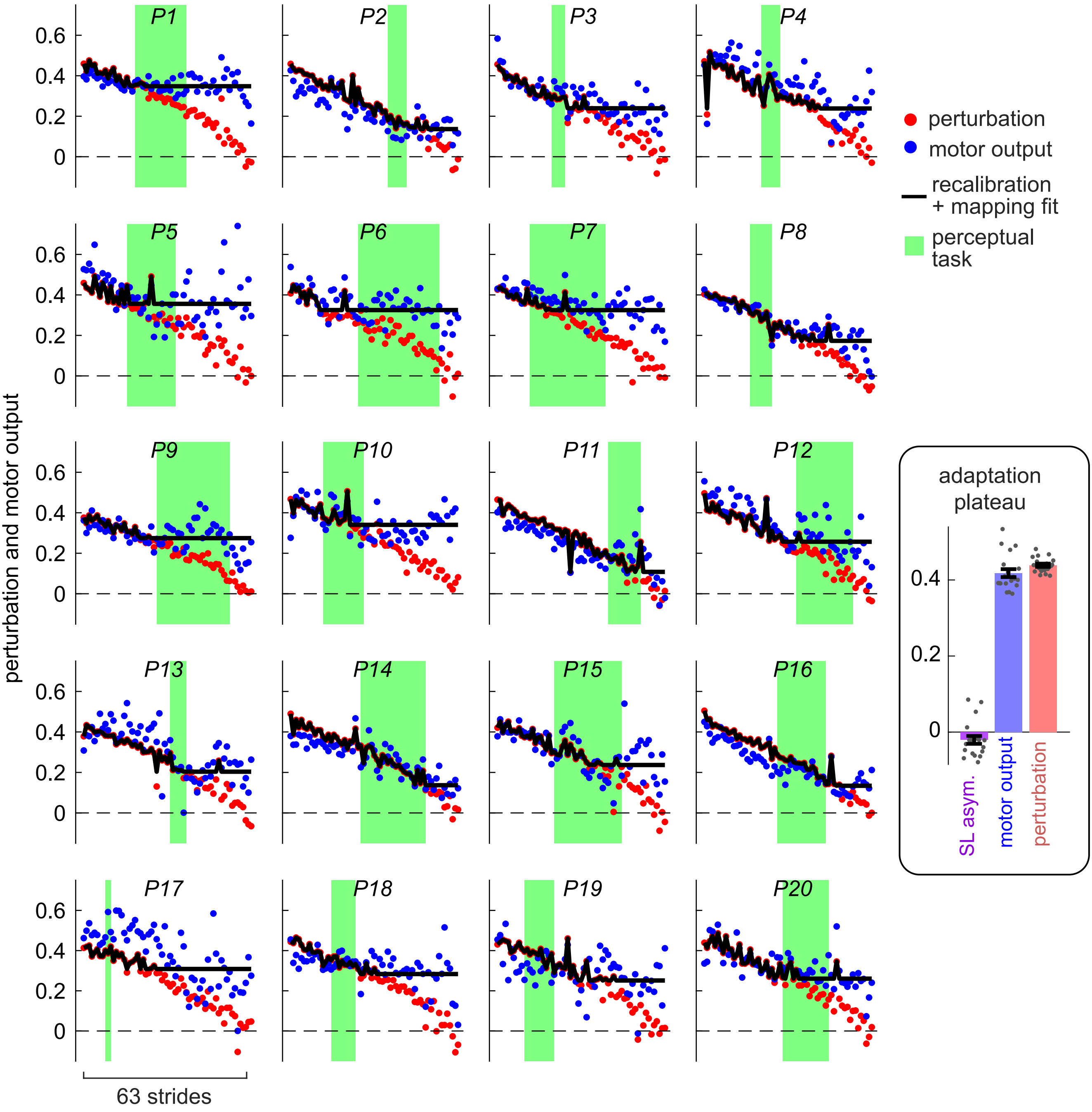

Figure S3. Experiment 1, individual participants' recalibration + mapping fits and perceptual results. Perturbation (red circles), motor output (blue circles) and recalibration + mapping model fitted to the motor output (black line), for the Ramp Down task. Green shaded area represents strides between button presses of the perceptual task. The insets show step length asymmetry, motor output, and perturbation at adaptation plateau (mean of the last 30 strides; circles are individual participants and error bars depict group mean ± SE).

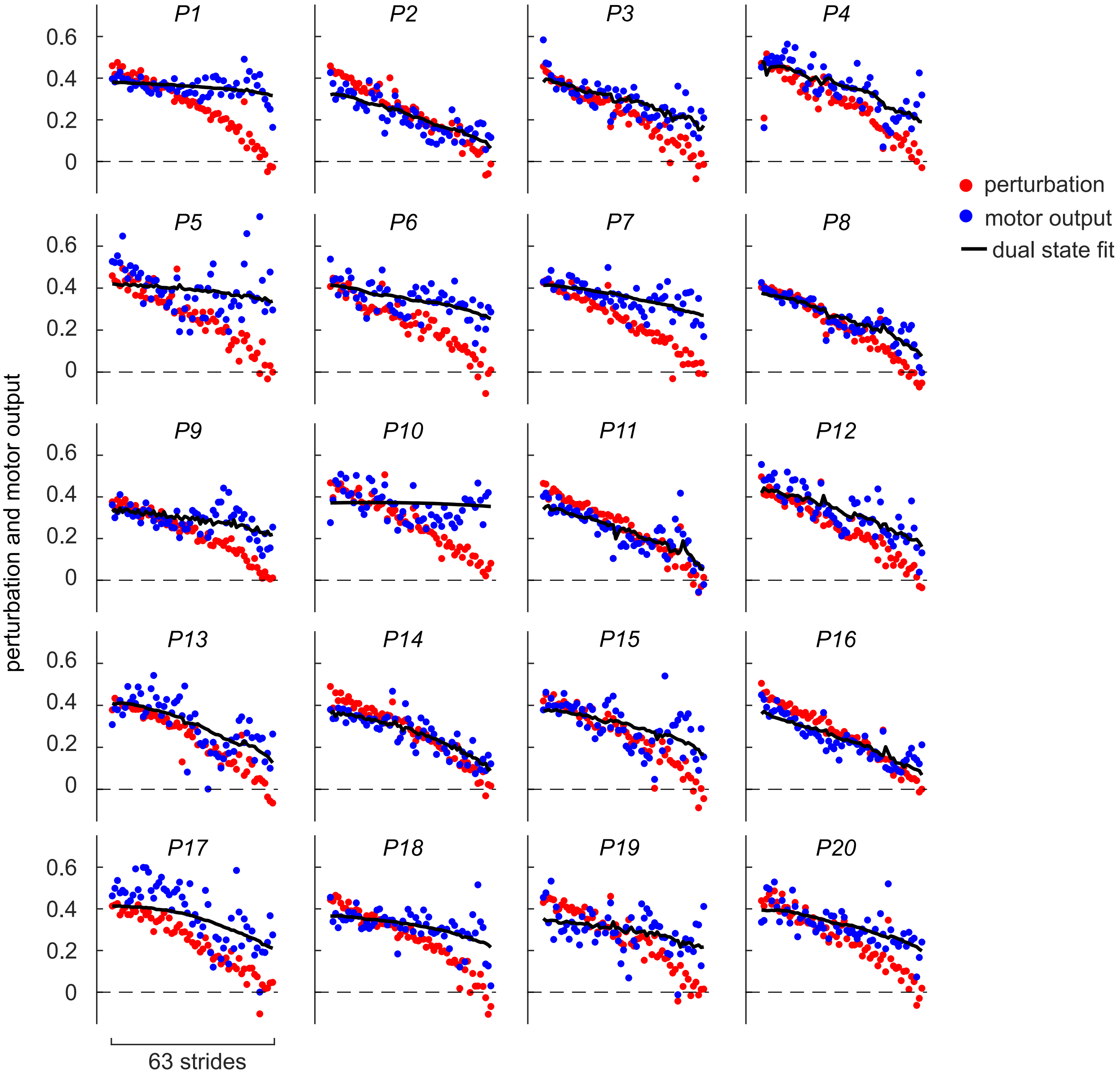

Figure S4. Experiment 1, individual participants' dual state fits. Perturbation (red circles), motor output (blue circles) and dual state model fitted to the motor output (black line), for the Ramp Down task.

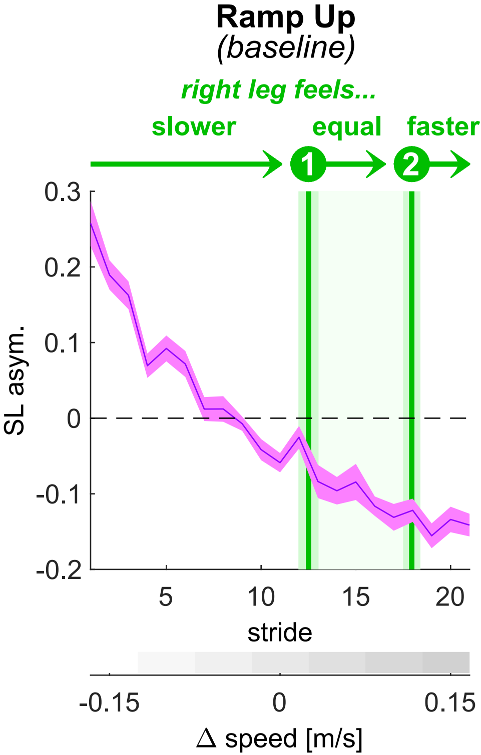

Figure S5. Experiment 1, baseline Ramp Up perceptual results (button presses mean ± SE, green vertical lines), overlayed on step length asymmetry (purple line and shade, mean ± SE).

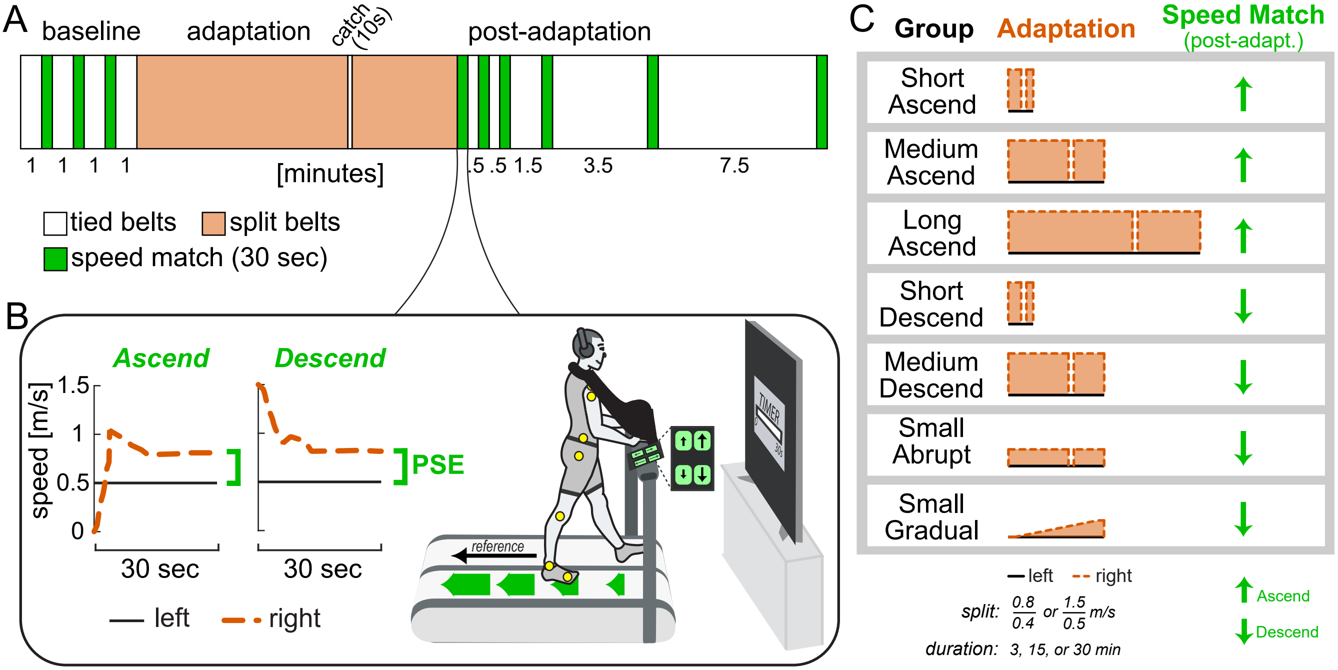

Figure S6. Control experiments, protocols. (A) General protocol for all Control experiments (tied belts = at the same speed, split belts = right faster than left). **(B)** Sample participants performing the ascend and descend versions of the speed match task (first iteration after adaptation). Participants respectively increased or decreased the speed of the right belt (dashed orange) using up/down buttons with the goal of matching it to the reference speed of the left belt (solid black). Vision and sound were occluded as shown. The PSE is the belt speed difference at the end of the task (green). **(C)** Adaptation belt speeds and post-adaptation speed match task version for all Control experiments. All groups except for Small Gradual experienced a catch-trial (tied belts) after two-thirds of the adaptation phase.

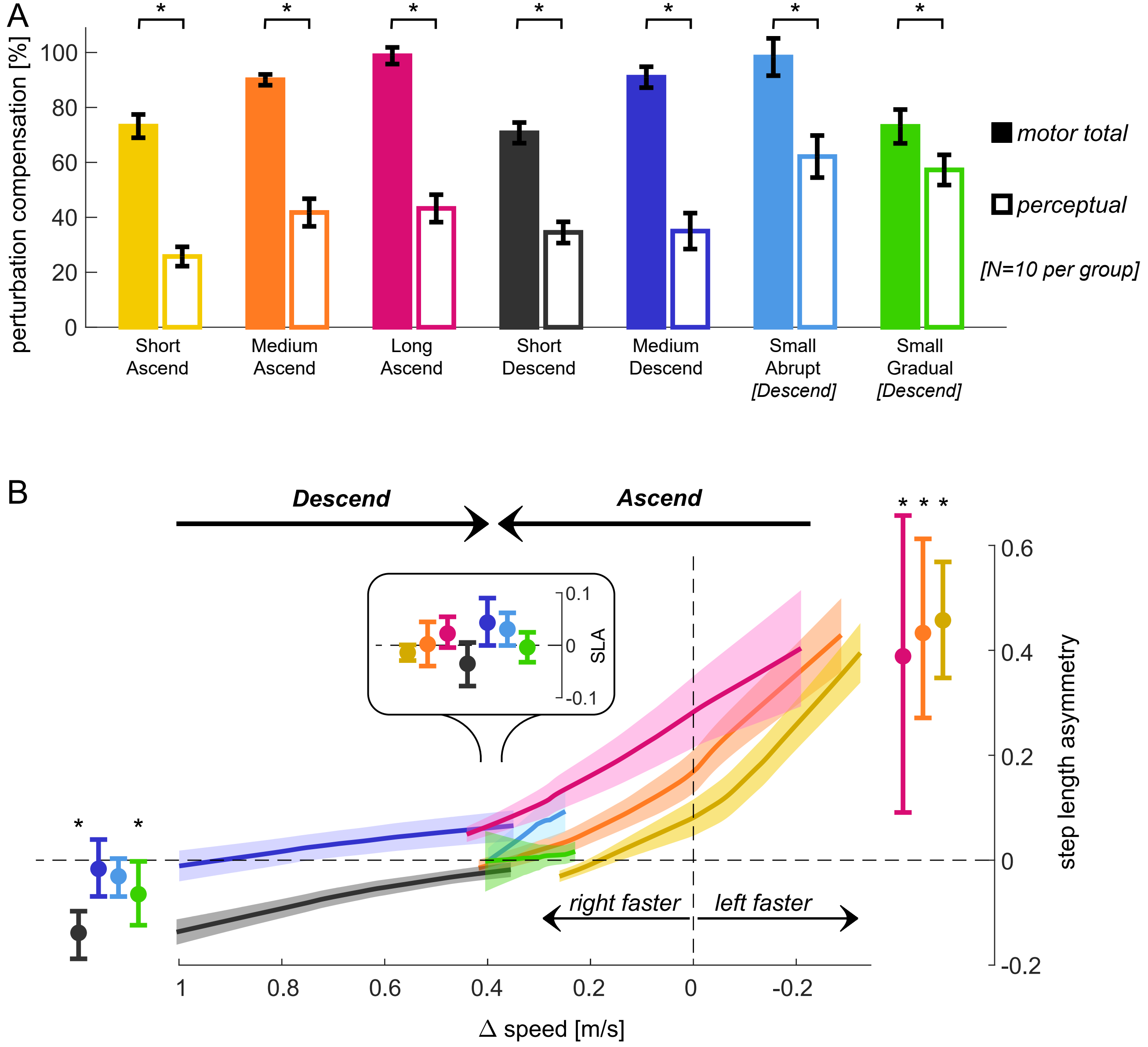

Figure S7. Control experiments, validation of Experiment 1. (A) compensation*motor total* (filled bars) and compensation*perceptual* (open bars) at the end of adaptation for all groups (group mean ± SE). **(B)** Step length asymmetry as a function of belt speed difference during the first post-adaptation speed match task (interpolated, group mean ± SE). Error bars depict step length asymmetry during the first (depicted to the right or left) and last (depicted in inset) strides in the task (actual data, group mean ± CI). Asterisks represent significant differences.

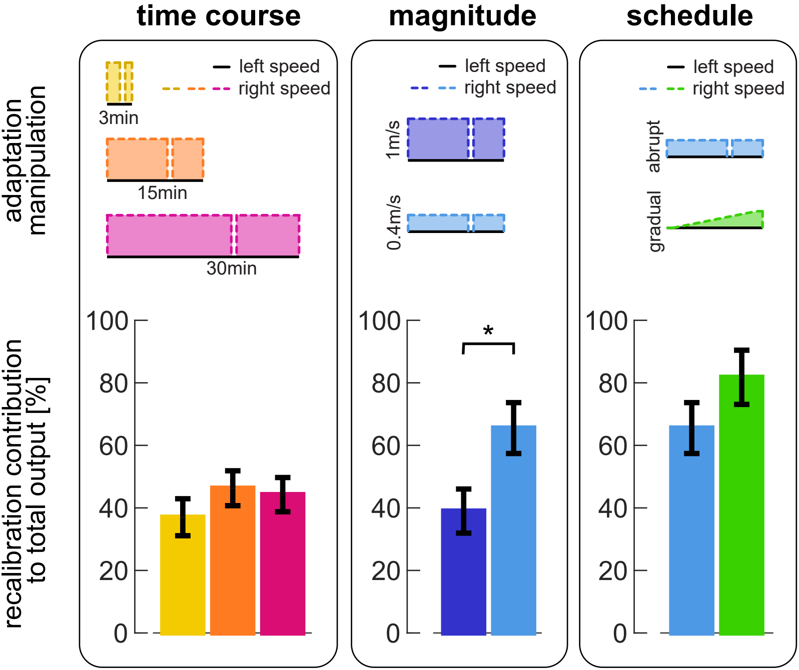

Figure S8. Control experiments, effect of paradigm manipulation on relative recalibration contribution. Top: Belt speeds in adaptation for selected experiments (left: Short, Medium, and Long Ascend; middle: Medium Descend and Small Abrupt; right: Small Abrupt and Gradual; see Fig. S5 for additional protocol details). **Bottom:** Comparison of the recalibration contribution to adaptation (compensation$_{perceptual}$ / compensation$_{motor\ total}$, group mean ± SE); asterisks represent significant differences.

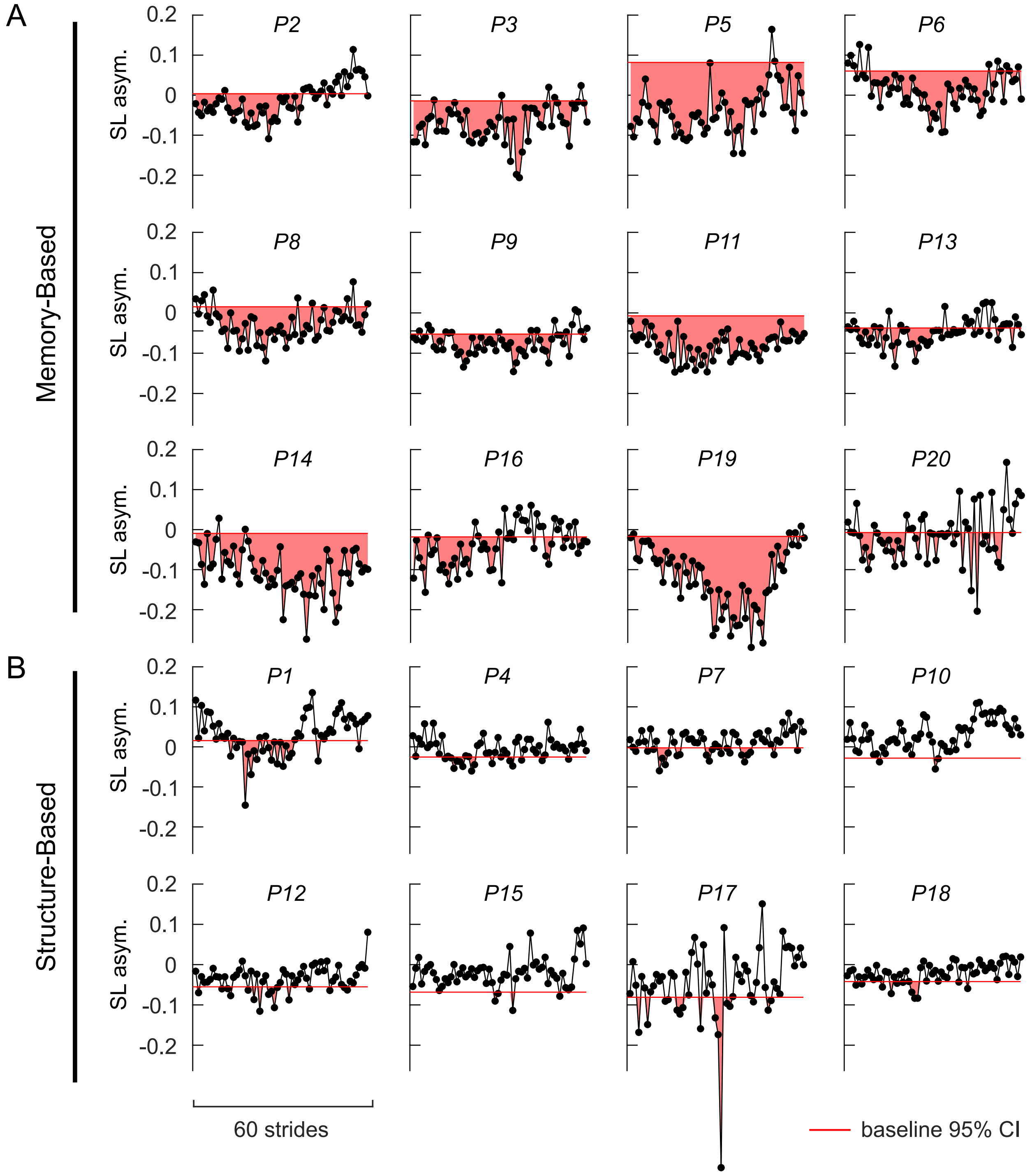

Figure S9. Experiment 2, individual participants' step length asymmetry for speed differences larger than adaptation. Step length asymmetry during the first (teal) portion of the Ramp Up and Down task (black circles and line), and participant's baseline 95% CI (lower bound, red line). Red shaded area represents the difference between task and baseline asymmetry for strides that had a more negative step length asymmetry than baseline.

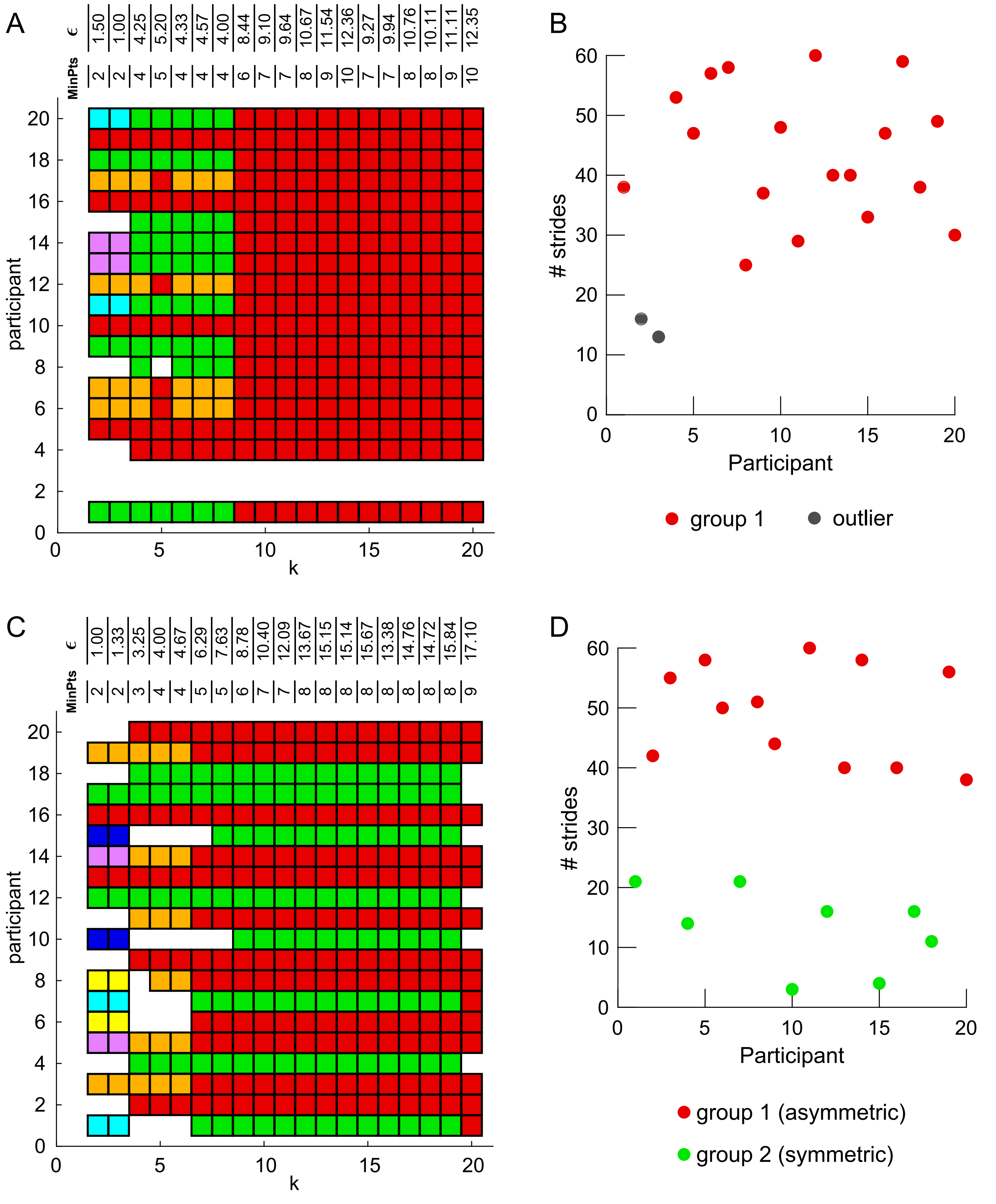

Figure S10. Primary clustering analysis for Experiment 1 (A&B) and 2 (C&D). (A&C) Cluster assignment (color of square) for each participant (y axis) computed with each 'k' iteration of the algorithm (x axis). The MinPts and Epsilon parameters computed and used for each 'k' iteration are reported on top of the graph. Different colors represent different clusters, and white spaces represent outliers. The algorithm selected the final cluster to be that for k=9 for both experiments; of note, results were identical for all 'k' iterations from 9 to 20 (A) or 9 to 19 (C). **(B&D)** Measure used for clustering (y-axis, # strides in ramp below/above baseline, see Methods) for each participant (x-axis), color-coded by cluster assignment.

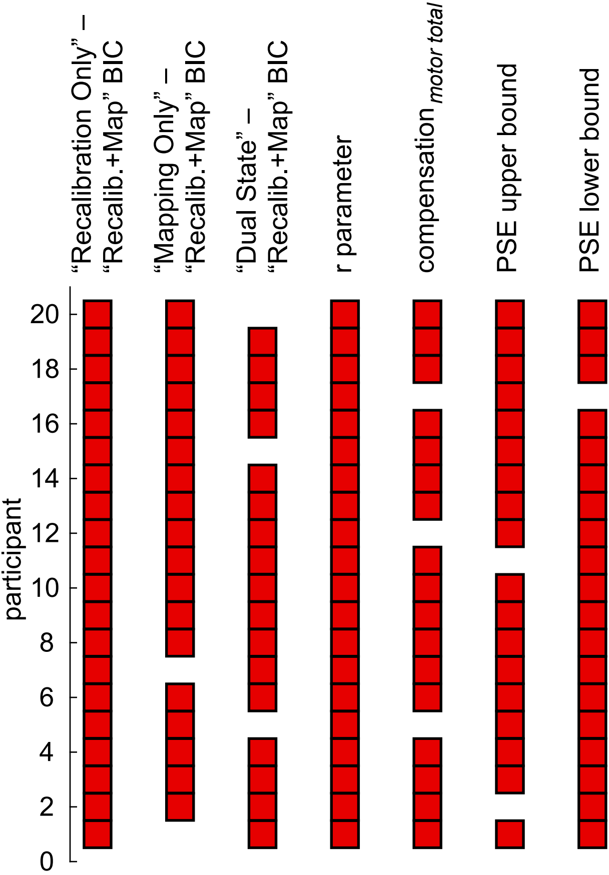

Figure S11. Secondary clustering analysis for Experiment 1. Participants clusters (after selection of the best k iteration) computed using different measures (reported above graph). Red boxes indicate participants assigned to cluster 1 and white spaces indicate outliers. For all measures, only one cluster was detected.

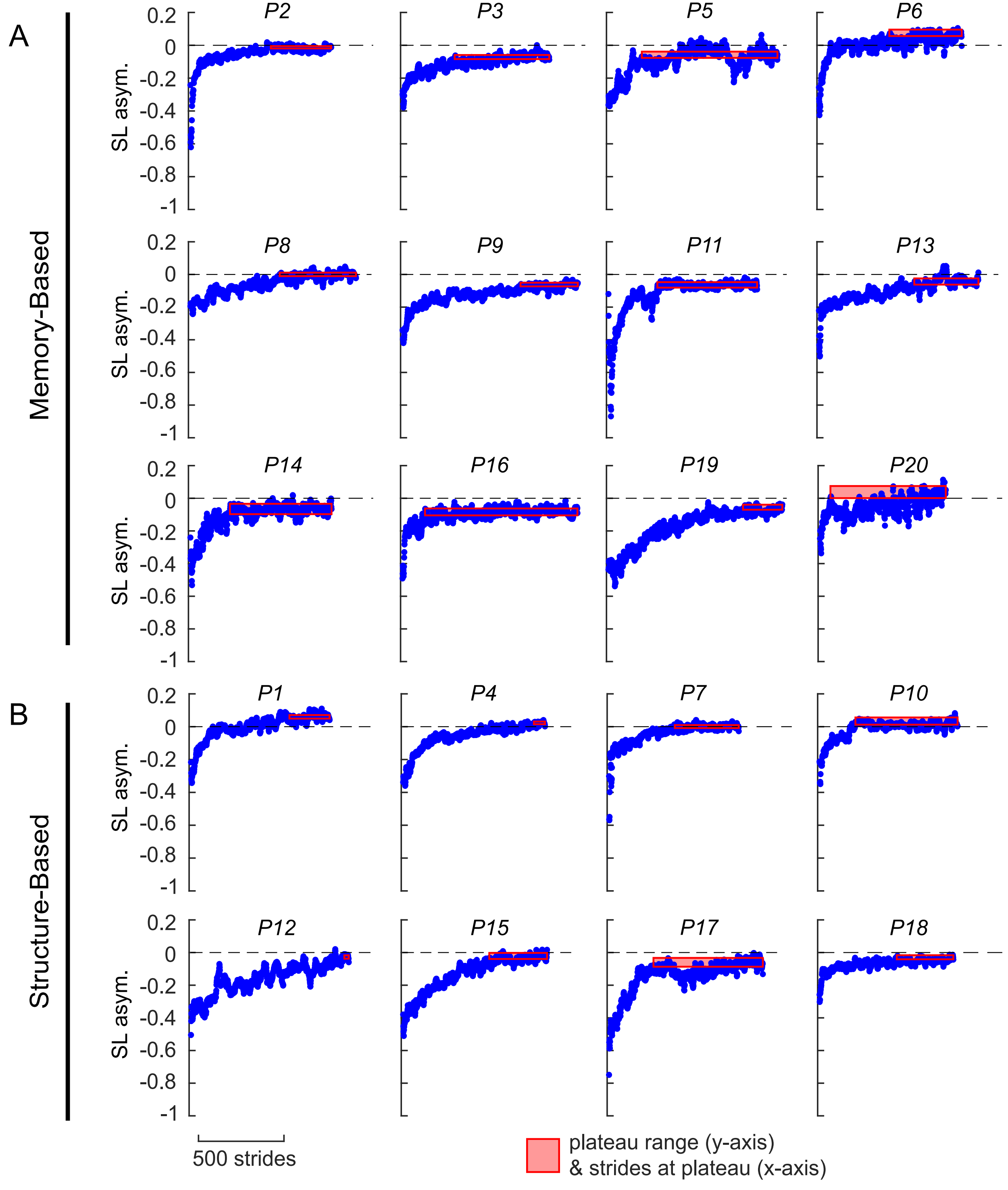

Figure S12. Experiment 2, individual participants' strides to plateau computation. Step length asymmetry in adaptation (blue circles), and plateau range (red rectangle; y-axis: plateau mean ± SD, x-axis: strides at plateau).

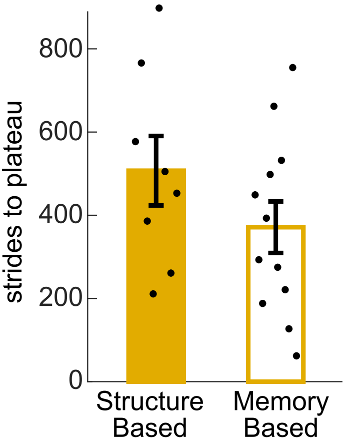

Figure S13. Experiment 2, comparison between subgroups of strides to plateau measure. Individual (black circles) and group mean ± SE (bars and error bars) strides to plateau measure divided by subgroup.
